# Supplementary material for: Intra-Species Genomic Variation in the Pine Pathogen Fusarium circinatum
Source: J Fungi (Basel). 2022 Jun 23;8(7):657. doi: 10.3390/jof8070657 (PMC9316270; doi:10.3390/jof8070657)
Supplement: Supplementary file 1 [file jof-08-00657-s001.zip › Supplementary Figure S1.pdf]

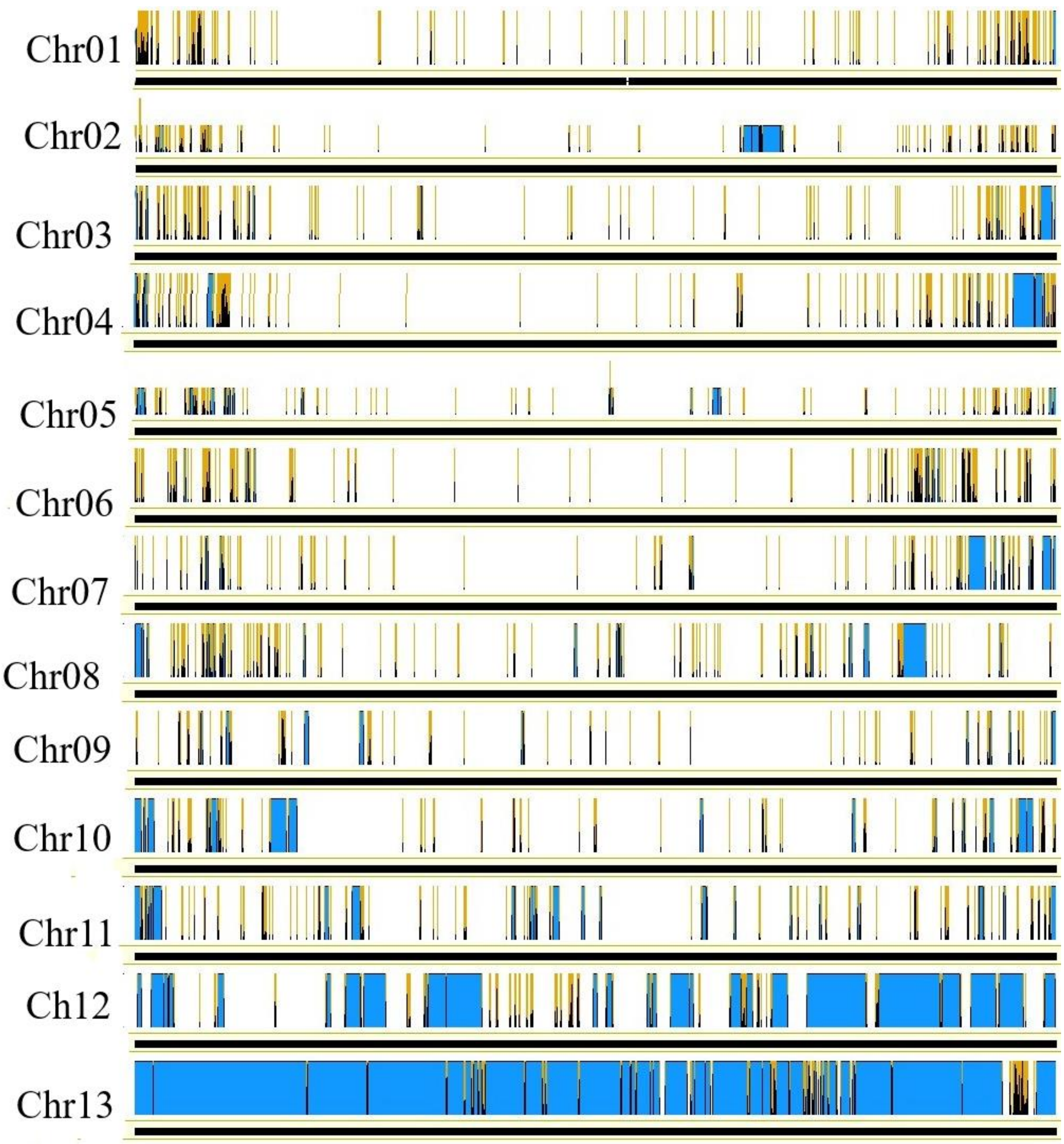

Supplementary Figure S1 Distribution of accessory genome elements across chromosomes of isolate CMW1803. Accessory genome elements are generally sparsely distributed within the middle parts of the chromosomes while the chromosome arms show a high density.
