# Supplementary material for: Intra-Species Genomic Variation in the Pine Pathogen Fusarium circinatum
Source: J Fungi (Basel). 2022 Jun 23;8(7):657. doi: 10.3390/jof8070657 (PMC9316270; doi:10.3390/jof8070657)
Supplement: Supplementary file 1 [file jof-08-00657-s001.zip › Supplementary S2.html]

### SnpEff: Variant analysis

|  |
| --- |
| **Contents** Summary   Variant rate by chromosome  Variants by type   Number of variants by impact    Number of variants by functional class    Number of variants by effect   Quality histogram  InDel length histogram  Base variant table  Transition vs transversions (ts/tv)   Allele frequency    Allele Count    Codon change table    Amino acid change table    Chromosome variants plots    Details by gene |


---


**Summary**

|  |  |
| --- | --- |
| **Genome** | fsp34 |
| **Date** | 2019-10-28 12:12 |
| **SnpEff version** | ``` SnpEff 4.3t (build 2017-11-24 10:18), by Pablo Cingolani ``` |
| **Command line arguments** | ``` SnpEff  fsp34 population_sniffles.vcf ``` |
| **Warnings** | 2 |
| **Errors** | 1 |
| **Number of lines (input file)** | 2,489 |
| **Number of variants (before filter)** | 1,770 |
| **Number of not variants  (i.e. reference equals alternative)** | 0 |
| **Number of variants processed   (i.e. after filter and non-variants)** | 1,770 |
| **Number of known variants  (i.e. non-empty ID)** | 1,109 ( 62.655% ) |
| **Number of multi-allelic VCF entries  (i.e. more than two alleles)** | 0 |
| **Number of effects** | 476,229 |
| **Genome total length** | 44,945,052 |
| **Genome effective length** | 44,943,885 |
| **Variant rate** | 1 variant every 25,392 bases |


---


 **Variants rate details** 

| Chromosome | Length | Variants | Variants rate |
| --- | --- | --- | --- |
| FSP34\_Chr01 | 6,407,589 | 214 | 29,942 |
| FSP34\_Chr02 | 5,065,897 | 198 | 25,585 |
| FSP34\_Chr03 | 5,081,688 | 188 | 27,030 |
| FSP34\_Chr04 | 4,313,068 | 147 | 29,340 |
| FSP34\_Chr05 | 4,432,453 | 183 | 24,221 |
| FSP34\_Chr06 | 4,301,695 | 197 | 21,836 |
| FSP34\_Chr07 | 3,540,654 | 144 | 24,587 |
| FSP34\_Chr08 | 3,172,515 | 159 | 19,952 |
| FSP34\_Chr09 | 2,981,544 | 107 | 27,864 |
| FSP34\_Chr10 | 2,698,620 | 103 | 26,200 |
| FSP34\_Chr11 | 2,228,220 | 83 | 26,846 |
| FSP34\_Chr12 | 525,065 | 35 | 15,001 |
| FSP34\_Contig01 | 85,668 | 3 | 28,556 |
| FSP34\_Contig02 | 27,708 | 3 | 9,236 |
| FSP34\_Mitochondrion | 81,501 | 6 | 13,583 |
| Total | 44,943,885 | 1,770 | 25,392 |


---


 **Number variants by type**

| **Type** | **Total** |
| --- | --- |
| **SNP** | 0 |
| **MNP** | 0 |
| **INS** | 0 |
| **DEL** | 990 |
| **MIXED** | 0 |
| **INV** | 111 |
| **DUP** | 8 |
| **BND** | 661 |
| **INTERVAL** | 0 |
| **Total** | 1,770 |
| --- | --- |


---


 **Number of effects by impact** 

| **Type (alphabetical order)** |  | Count | Percent |
| --- | --- | --- | --- |
| **HIGH** |  | 420,017 | 88.196% |
| **LOW** |  | 1 | 0% |
| **MODERATE** |  | 49,895 | 10.477% |
| **MODIFIER** |  | 6,316 | 1.326% |


---


 **Number of effects by functional class** 

| **Type (alphabetical order)** |  | Count | Percent |
| --- | --- | --- | --- |

  

Missense / Silent ratio: 0


---


 **Number of effects by type and region** 

| Type | Region |
| --- | --- |
| | **Type (alphabetical order)** |  | Count | Percent | | --- | --- | --- | --- | | **bidirectional\_gene\_fusion** |  | 222,121 | 46.62% | | **chromosome\_number\_variation** |  | 10 | 0.002% | | **conservative\_inframe\_deletion** |  | 103 | 0.022% | | **disruptive\_inframe\_deletion** |  | 57 | 0.012% | | **downstream\_gene\_variant** |  | 2,499 | 0.525% | | **duplication** |  | 1 | 0% | | **exon\_loss\_variant** |  | 100 | 0.021% | | **exon\_region** |  | 2 | 0% | | **feature\_ablation** |  | 7,724 | 1.621% | | **frameshift\_variant** |  | 149 | 0.031% | | **gene\_fusion** |  | 180,468 | 37.878% | | **intergenic\_region** |  | 608 | 0.128% | | **intragenic\_variant** |  | 367 | 0.077% | | **intron\_variant** |  | 49 | 0.01% | | **inversion** |  | 49,863 | 10.466% | | **non\_coding\_transcript\_variant** |  | 310 | 0.065% | | **splice\_acceptor\_variant** |  | 16 | 0.003% | | **splice\_donor\_variant** |  | 19 | 0.004% | | **splice\_region\_variant** |  | 68 | 0.014% | | **start\_lost** |  | 45 | 0.009% | | **stop\_gained** |  | 1 | 0% | | **stop\_lost** |  | 39 | 0.008% | | **transcript\_ablation** |  | 9,316 | 1.955% | | **upstream\_gene\_variant** |  | 2,515 | 0.528% | | | **Type (alphabetical order)** |  | Count | Percent | | --- | --- | --- | --- | | **CHROMOSOME** |  | 49 | 0.01% | | **DOWNSTREAM** |  | 2,499 | 0.525% | | **EXON** |  | 449 | 0.094% | | **GENE** |  | 438,343 | 92.045% | | **INTERGENIC** |  | 608 | 0.128% | | **INTRON** |  | 15 | 0.003% | | **SPLICE\_SITE\_ACCEPTOR** |  | 5 | 0.001% | | **SPLICE\_SITE\_DONOR** |  | 6 | 0.001% | | **SPLICE\_SITE\_REGION** |  | 1 | 0% | | **TRANSCRIPT** |  | 31,739 | 6.665% | | **UPSTREAM** |  | 2,515 | 0.528% | |


---


 **Quality:**

```
	
```


---


 **Insertions and deletions length:**

```
|  |  |
| --- | --- |
| Min | 1 |
| Max | 1 |
| Mean | 1 |
| Median | 1 |
| Standard deviation | 0 |
| Values | 1 |
| Count | 990 |
```


---


 **Base changes (SNPs)** 

|  |  |  |  |  |
| --- | --- | --- | --- | --- |
|  | **A** | **C** | **G** | **T** |
| **A** | 0 | 0 | 0 | 0 |
| **C** | 0 | 0 | 0 | 0 |
| **G** | 0 | 0 | 0 | 0 |
| **T** | 0 | 0 | 0 | 0 |

---


  **Ts/Tv (transitions / transversions)** 

**Note:** Only SNPs are used for this statistic.  
**Note:** This Ts/Tv ratio is a 'raw' ratio (ratio of observed events).

|  |  |
| --- | --- |
| Transitions | 0 |
| Transversions | 0 |
| Ts/Tv ratio | 0 |

**All variants:**

```
No results available (empty input?)
```

**Only known variants** (i.e. the ones having a non-empty ID field):

```
No results available (empty input?)
```

---


  **Allele frequency** 
  

|  |  |
| --- | --- |
| Min | 0 |
| Max | 100 |
| Mean | 39.452 |
| Median | 33 |
| Standard deviation | 30.477 |
| Values | 0,8,16,20,25,33,40,41,50,58,60,66,70,75,80,83,91,100 |
| Count | 195,94,777,3,45,409,2,44,229,39,5,189,2,35,1,156,26,238 |

---


  **Allele Count** 
  

|  |  |
| --- | --- |
| Min | 0 |
| Max | 12 |
| Mean | 4.727 |
| Median | 4 |
| Standard deviation | 3.61 |
| Values | 0,1,2,3,4,5,6,7,8,9,10,11,12 |
| Count | 195,94,790,44,413,44,234,41,191,35,158,26,224 |

---


  **Hom/Het per sample** 
  
  
  

```
Sample_names , ../cmw1803/cmw1803_fsp34ngmlr.bam, ../cmw560/cmw560_fsp34ngmlr.bam, ../cmw567/cmw567_fsp34ngmlr.bam, ../ks17/ks17_fsp34ngmlr.bam, ../ug10/ug10_fsp34ngmlr.bam, ../ug27/ug27_fsp34ngmlr.bam
Reference , 1440, 1331, 1364, 1587, 1509, 1400
Het , 88, 124, 82, 79, 113, 138
Hom , 943, 1016, 1025, 805, 849, 933
Missing , 18, 18, 18, 18, 18, 18
```

---


 **Codon changes**

How to read this table:   
- Rows are reference codons and columns are changed codons. E.g. Row 'AAA' column 'TAA' indicates how many 'AAA' codons have been replaced by 'TAA' codons.  
- Red background colors indicate that more changes happened (heat-map).  
- Diagonals are indicated using grey background color   
- WARNING: This table may include different translation codon tables (e.g. mamalian DNA and mitochondrial DNA).

|  | - | AAA | AAC | AAG | AAT | ACA | ACC | ACG | ACT | AGA | AGC | AGG | AGT | ATA | ATC | ATG | ATT | CAA | CAC | CAG | CAT | CCA | CCC | CCG | CCT | CGA | CGC | CGG | CGT | CTA | CTC | CTG | CTT | GAA | GAC | GAG | GAT | GCA | GCC | GCG | GCT | GGA | GGC | GGG | GGT | GTA | GTC | GTG | GTT | TAA | TAC | TAG | TAT | TCA | TCC | TCG | TCT | TGA | TGC | TGG | TGT | TTA | TTC | TTG | TTT |
| --- | --- | --- | --- | --- | --- | --- | --- | --- | --- | --- | --- | --- | --- | --- | --- | --- | --- | --- | --- | --- | --- | --- | --- | --- | --- | --- | --- | --- | --- | --- | --- | --- | --- | --- | --- | --- | --- | --- | --- | --- | --- | --- | --- | --- | --- | --- | --- | --- | --- | --- | --- | --- | --- | --- | --- | --- | --- | --- | --- | --- | --- | --- | --- | --- | --- |
| - |  | 44 | 58 | 71 | 41 | 39 | 27 | 36 | 52 | 24 | 42 | 19 | 34 | 20 | 62 | 46 | 39 | 36 | 20 | 47 | 22 | 49 | 18 | 29 | 34 | 24 | 18 | 11 | 17 | 31 | 46 | 49 | 52 | 49 | 87 | 84 | 104 | 44 | 47 | 33 | 54 | 44 | 65 | 36 | 49 | 19 | 47 | 30 | 47 | 3 | 47 | 6 | 40 | 41 | 32 | 33 | 46 | 3 | 20 | 51 | 15 | 21 | 57 | 43 | 37 |
| AAA | 369 | 4 | 6 | 8 |  | 2 | 4 | 4 | 3 | 6 | 6 | 5 | 2 | 2 | 3 | 6 | 5 | 4 | 2 | 2 | 3 | 3 | 1 | 2 | 3 | 2 |  | 3 | 3 | 5 | 2 | 2 | 4 | 2 |  | 4 | 6 | 2 | 4 | 2 | 2 | 2 | 1 | 6 | 1 | 1 | 3 | 3 | 2 | 2 | 3 | 2 | 1 | 5 | 3 | 1 | 6 | 3 | 4 | 3 |  | 1 | 4 | 8 | 2 |
| AAC | 559 | 3 | 6 | 3 | 7 | 5 | 10 | 2 | 7 | 4 | 3 | 1 | 7 | 5 | 6 | 6 | 3 | 9 | 5 | 4 | 8 | 5 | 3 | 3 | 6 | 3 | 5 | 5 | 1 | 4 | 5 | 5 | 2 | 4 | 5 | 7 | 2 | 4 | 4 | 2 | 7 | 7 | 5 | 4 | 2 | 1 | 7 | 4 | 1 | 5 | 4 | 1 | 5 | 10 | 5 | 3 | 8 | 2 |  | 3 | 2 | 2 | 8 | 6 | 2 |
| AAG | 803 | 3 | 7 | 9 | 5 | 2 | 4 | 5 | 11 | 5 | 8 | 5 |  | 6 | 13 | 8 | 5 | 5 | 5 | 7 | 6 | 9 | 5 | 6 | 6 | 13 | 4 | 5 | 2 | 2 | 4 | 9 | 9 | 7 | 3 | 5 | 5 | 6 | 6 | 6 | 9 | 6 | 5 | 1 | 1 | 1 | 3 | 10 | 6 | 1 | 7 | 3 | 7 | 7 | 6 | 5 | 4 | 3 | 7 | 11 | 5 | 4 | 11 | 9 | 5 |
| AAT | 385 | 2 | 3 | 8 | 7 | 6 | 5 | 5 | 3 | 8 | 2 | 3 | 1 | 3 | 3 | 6 | 3 | 2 | 2 | 4 |  | 3 | 5 | 2 | 4 | 2 |  | 3 | 3 |  | 3 | 6 | 7 | 5 | 4 |  | 2 | 4 | 3 | 3 | 2 | 3 | 6 | 5 | 1 | 3 | 3 | 2 | 5 | 2 |  | 4 | 2 | 4 | 4 | 2 | 2 | 4 | 1 | 7 |  | 2 | 4 |  | 4 |
| ACA | 474 | 4 | 4 | 9 | 5 | 7 | 3 | 1 | 3 | 5 | 9 | 5 | 3 | 4 | 5 | 5 |  | 7 | 3 | 7 | 1 | 2 | 1 | 5 | 4 | 8 | 3 | 3 |  | 3 | 3 | 4 | 5 | 2 |  | 2 | 2 | 3 | 3 | 7 | 4 | 5 | 5 | 3 | 2 | 2 | 3 | 4 | 2 | 2 | 3 |  | 2 | 3 |  | 1 | 4 | 4 | 1 | 6 | 3 |  | 2 | 4 | 1 |
| ACC | 561 | 4 |  | 4 | 2 | 5 | 3 | 2 | 3 | 4 | 3 | 4 | 4 | 7 | 2 | 6 | 4 | 2 | 4 | 2 | 1 |  | 4 | 4 | 5 | 2 | 1 | 6 | 1 |  | 6 | 5 | 4 | 4 | 1 | 2 |  | 3 | 3 | 2 | 3 | 4 | 4 | 3 |  | 5 | 4 | 2 | 2 |  | 2 | 4 | 3 | 6 | 2 | 4 | 5 | 4 | 5 | 1 | 2 | 4 | 3 | 3 | 1 |
| ACG | 332 | 2 | 1 | 5 | 3 | 2 |  | 3 | 4 | 1 | 4 | 2 |  | 2 | 1 | 3 | 2 | 2 | 2 | 4 | 4 | 4 | 2 |  | 1 | 1 | 1 | 1 | 1 | 1 |  | 3 | 1 | 3 | 1 | 4 |  | 2 | 3 | 2 | 2 | 4 |  | 2 | 2 | 3 |  | 1 | 2 |  | 3 | 1 | 1 | 7 | 3 | 4 | 6 | 3 | 2 | 3 | 3 | 2 | 4 | 5 | 2 |
| ACT | 603 | 4 | 3 | 3 | 6 | 7 | 6 | 5 | 10 | 4 | 6 | 6 | 4 | 5 | 6 | 10 | 1 | 4 | 5 | 3 | 5 | 7 | 2 | 7 | 3 | 4 | 3 | 3 | 3 | 5 | 4 | 9 | 2 | 1 | 1 |  | 6 | 7 | 2 |  | 4 | 2 | 4 |  | 4 | 4 | 4 | 1 | 5 |  | 7 | 2 | 2 | 3 | 4 | 3 | 2 | 3 | 2 | 2 | 3 | 1 | 6 | 4 | 4 |
| AGA | 309 | 2 | 1 | 1 | 4 | 2 | 2 | 1 | 1 | 4 | 2 | 3 | 2 | 2 | 2 | 1 | 4 | 4 | 2 | 2 | 2 | 5 | 4 | 4 | 5 | 4 | 4 | 1 | 1 | 1 |  | 3 |  | 1 | 6 | 2 | 2 | 4 | 2 |  | 4 | 3 | 4 | 2 | 2 | 3 | 3 | 2 | 3 | 2 | 1 | 2 |  | 5 | 3 | 6 | 3 | 3 | 4 | 5 |  | 1 | 7 | 3 |  |
| AGC | 404 | 3 | 7 | 2 | 2 | 4 | 6 | 1 | 4 | 4 | 6 | 4 | 3 | 3 | 3 | 2 | 6 | 6 | 3 | 1 | 2 | 6 | 3 | 1 | 2 | 1 | 2 | 3 | 3 | 2 | 4 | 2 | 2 | 4 | 3 | 4 | 1 | 1 | 2 |  | 1 | 1 | 3 | 3 | 2 | 1 | 2 | 3 | 3 |  | 3 | 1 | 8 | 4 | 4 | 3 | 6 | 5 | 1 | 2 | 2 | 2 | 2 | 3 | 2 |
| AGG | 218 | 2 | 2 | 2 |  | 3 |  | 2 | 1 | 2 |  | 1 | 1 | 3 | 1 | 4 | 1 | 3 | 3 | 2 | 2 | 2 |  |  | 2 | 3 | 2 | 1 | 1 | 3 | 1 | 3 | 1 | 2 |  | 5 | 1 | 2 |  | 2 | 1 | 2 |  |  | 3 | 5 |  |  | 3 |  | 3 | 2 | 3 | 1 | 3 | 1 | 3 | 2 | 3 | 2 |  | 3 | 2 | 2 | 4 |
| AGT | 317 | 1 | 5 | 1 | 2 | 4 | 1 | 2 | 1 |  | 4 | 8 | 1 |  | 2 | 3 | 2 | 5 | 3 | 4 | 5 | 2 |  |  | 5 | 1 | 1 |  |  | 1 | 1 | 7 | 5 | 1 | 2 | 3 | 2 | 4 | 5 | 1 | 6 | 2 | 3 |  | 1 |  | 1 | 2 | 2 |  |  | 2 | 2 | 6 | 1 | 5 | 2 | 1 | 5 | 3 | 5 | 1 | 3 | 3 | 1 |
| ATA | 263 | 3 | 2 | 5 | 3 | 1 | 2 | 1 | 4 | 2 | 2 | 3 | 2 | 1 | 1 | 4 | 2 | 3 | 3 | 2 | 1 | 2 | 2 | 4 | 1 |  |  |  |  | 1 | 3 | 2 | 1 | 2 | 2 | 5 |  | 1 | 3 | 1 | 1 | 2 | 4 | 2 |  | 2 | 1 |  | 2 | 1 | 1 | 2 | 2 | 3 | 5 | 1 | 2 | 5 | 1 | 2 | 2 |  | 1 | 8 | 2 |
| ATC | 673 | 3 | 5 | 8 | 3 | 5 | 5 | 14 | 8 | 8 | 3 | 4 | 3 | 6 | 7 | 6 | 6 | 9 | 6 | 5 | 3 | 4 | 6 | 4 | 4 | 6 | 3 | 7 | 2 | 6 | 7 | 4 | 4 | 5 | 7 | 5 | 5 | 5 | 3 | 8 | 5 | 4 | 3 | 2 | 8 | 6 | 4 | 7 | 7 | 1 | 9 | 2 | 3 | 9 | 6 | 4 | 4 | 1 | 7 | 8 | 3 | 4 | 8 | 7 | 1 |
| ATG | 538 | 7 | 4 | 6 | 7 | 4 | 2 | 6 | 2 | 3 | 7 | 4 | 8 | 3 | 6 | 5 | 3 | 6 | 5 | 7 | 3 | 3 | 4 | 2 | 2 | 3 | 1 | 3 | 3 | 5 | 9 | 5 | 4 | 1 | 4 | 3 | 2 | 3 | 1 | 1 | 2 | 4 | 2 | 2 | 6 | 4 |  | 1 | 2 | 2 | 7 | 2 | 5 | 4 | 7 | 6 | 10 | 7 | 3 | 4 | 5 | 3 | 6 | 6 | 1 |
| ATT | 490 | 6 | 9 | 5 | 1 | 4 | 9 | 5 | 1 | 11 | 6 | 7 | 2 | 5 | 1 | 8 |  | 3 | 3 | 4 | 2 | 6 | 1 | 5 | 2 | 8 | 4 | 4 | 1 | 6 | 7 | 6 | 1 | 2 | 4 | 5 | 2 | 1 | 3 | 2 | 3 | 2 | 3 | 4 | 2 | 1 | 2 | 3 | 8 | 2 | 4 | 1 | 5 | 8 | 4 | 3 | 6 | 7 | 1 | 9 | 3 | 3 | 5 | 9 | 2 |
| CAA | 500 | 7 | 2 | 2 | 4 | 5 | 4 | 5 | 1 | 8 | 5 | 3 | 1 | 5 | 7 | 7 | 3 | 5 | 4 | 10 | 3 | 6 | 2 |  | 6 | 3 | 1 | 6 | 2 | 4 | 4 | 5 | 3 | 2 | 3 | 4 | 4 | 4 | 7 | 3 | 3 | 7 | 5 | 2 | 3 | 2 | 3 | 6 | 1 | 2 | 2 |  | 3 | 7 | 6 | 4 | 6 | 5 | 4 | 6 | 3 | 5 | 7 | 3 | 4 |
| CAC | 265 | 2 | 2 | 2 | 3 | 1 | 2 | 2 | 4 | 3 | 1 | 3 | 2 | 3 | 8 | 2 | 1 | 1 | 2 | 3 | 1 | 4 | 3 | 4 | 2 | 3 | 2 | 1 | 1 | 4 | 1 | 8 | 3 |  |  | 4 | 5 | 2 | 4 | 1 | 3 | 4 |  | 1 |  | 2 | 4 |  | 1 | 1 | 3 |  | 3 | 2 | 1 | 2 | 2 | 2 | 2 | 5 | 2 | 1 | 8 | 8 | 2 |
| CAG | 567 | 4 | 2 | 3 | 2 | 4 | 6 | 3 | 5 | 2 | 3 | 7 | 2 | 3 | 5 | 6 | 2 | 8 | 6 | 4 | 4 | 3 | 4 | 2 | 4 | 2 | 5 | 2 | 3 | 4 | 6 | 3 | 4 | 6 | 2 | 5 | 1 | 2 | 2 |  | 5 | 3 | 5 | 4 | 3 | 2 | 2 | 2 | 4 | 2 | 4 | 4 | 6 | 9 | 2 | 2 | 3 | 9 | 1 | 7 | 4 | 2 | 5 | 10 | 8 |
| CAT | 324 | 4 | 3 | 2 | 4 | 4 |  | 4 | 3 | 4 | 4 | 4 | 2 | 3 | 4 | 3 |  | 3 | 4 | 1 | 3 | 2 | 1 | 1 | 5 | 1 | 1 | 2 | 5 | 3 | 2 | 4 | 3 | 2 | 1 | 4 | 4 | 3 | 4 |  | 2 | 4 | 2 |  |  | 3 | 1 | 3 | 1 | 1 | 3 | 4 | 1 | 7 | 4 | 6 | 3 | 2 | 1 | 3 | 3 | 2 | 5 |  | 4 |
| CCA | 424 | 1 | 3 | 5 |  | 3 |  | 5 | 1 | 6 | 4 | 2 |  | 3 | 2 | 6 | 1 | 6 | 5 | 4 | 1 | 3 | 3 | 3 | 7 | 2 | 5 | 2 | 5 |  | 4 | 5 | 4 | 2 | 4 | 2 | 3 | 5 | 3 | 1 | 3 | 2 | 3 | 2 | 2 | 2 | 1 | 2 | 2 | 1 | 4 |  | 4 | 7 | 1 | 6 | 4 | 1 | 7 | 7 | 4 | 2 | 5 | 6 | 5 |
| CCC | 353 | 1 | 1 | 3 | 1 | 2 | 4 | 4 | 1 | 3 | 1 | 2 |  | 5 | 3 | 4 | 4 | 2 | 6 | 4 | 4 | 10 | 4 |  | 4 | 1 | 3 | 3 |  | 5 | 2 | 5 | 3 | 2 | 3 | 2 | 6 | 1 | 2 | 1 | 2 | 3 | 1 | 4 | 4 | 1 |  | 1 | 1 | 1 | 5 | 2 | 1 | 3 | 1 | 2 | 3 | 3 | 2 | 4 | 6 | 3 | 3 | 4 | 2 |
| CCG | 200 | 3 | 5 | 3 | 1 | 4 | 1 | 3 | 3 | 2 | 1 |  | 1 |  | 1 | 2 | 3 | 2 |  |  | 3 | 4 | 1 | 2 | 1 | 2 | 1 |  | 1 | 1 | 4 | 3 | 1 | 4 | 2 | 2 | 3 | 2 |  | 1 |  | 1 | 3 | 2 |  | 2 |  |  | 1 | 1 |  | 2 | 2 | 2 | 2 | 3 | 1 | 3 | 2 | 1 | 2 | 1 | 3 | 1 | 2 |
| CCT | 538 | 4 | 2 | 10 | 3 | 4 | 2 | 1 | 4 | 3 | 2 | 4 | 1 | 2 | 8 | 9 | 2 | 5 | 6 | 4 | 3 | 5 | 2 |  | 10 | 3 |  | 2 | 2 | 4 | 3 | 6 | 1 | 4 | 2 | 3 | 2 | 3 | 2 | 1 | 4 | 4 | 4 | 2 | 6 | 3 | 3 | 2 | 4 | 3 | 3 |  | 3 | 5 | 4 | 2 | 5 | 3 | 3 | 3 | 2 | 4 | 8 | 1 | 2 |
| CGA | 285 | 3 | 2 | 1 | 2 | 2 | 2 | 4 | 1 | 3 | 2 | 3 | 1 | 3 | 5 | 2 |  | 7 | 3 | 3 | 5 | 2 | 1 | 2 | 2 | 4 | 2 |  | 2 | 3 | 3 | 2 | 3 | 2 | 2 | 1 | 2 | 2 |  | 3 | 2 | 4 |  | 5 |  | 2 | 2 | 2 | 2 | 1 | 3 | 1 | 1 | 1 | 1 | 2 | 5 | 4 | 3 | 3 | 4 | 2 |  | 1 | 4 |
| CGC | 266 | 1 | 2 | 4 |  | 1 | 1 | 4 | 2 | 3 | 4 | 3 | 3 | 3 | 3 | 4 | 3 | 3 | 2 | 1 | 1 | 1 | 2 | 1 | 1 | 2 | 1 | 2 | 2 | 2 | 2 | 2 | 1 | 5 | 5 | 1 | 1 | 3 | 2 |  |  | 1 | 6 |  |  | 1 | 1 | 3 | 1 | 1 | 1 |  |  | 3 | 2 | 2 | 3 | 3 | 3 | 2 |  |  | 1 | 4 | 1 |
| CGG | 156 | 1 |  | 1 | 1 | 2 |  | 1 | 3 | 2 | 3 | 2 | 1 | 1 | 3 |  | 1 | 2 | 1 | 4 | 3 | 1 | 1 | 1 | 3 | 1 |  |  |  |  |  | 1 |  |  | 3 | 2 | 1 |  | 1 |  |  | 1 | 1 |  | 1 | 1 | 2 | 1 |  |  | 2 | 1 |  | 5 | 2 | 1 | 1 | 3 | 3 | 1 | 1 | 3 |  | 4 | 1 |
| CGT | 211 | 1 | 1 | 2 |  | 1 | 1 | 1 |  | 3 | 3 | 2 |  |  | 4 | 4 | 1 |  |  | 3 | 1 | 3 | 2 | 1 | 2 |  | 1 | 1 | 1 |  | 2 |  | 2 | 2 | 1 | 4 | 1 |  | 1 | 2 | 1 | 2 | 1 |  | 1 | 1 | 2 | 1 |  | 1 | 2 | 1 |  | 4 | 1 | 2 | 3 | 1 | 2 | 2 | 1 | 2 | 3 | 3 | 1 |
| CTA | 253 |  | 4 | 2 | 1 | 2 | 2 | 2 | 2 | 5 | 3 | 5 | 2 | 2 | 6 | 7 | 1 | 2 | 1 | 4 | 4 | 3 | 1 | 2 | 2 | 5 | 3 | 3 | 1 | 2 | 2 | 1 | 1 |  | 1 |  | 1 | 1 |  | 3 | 1 | 5 | 3 | 3 | 2 | 2 | 3 | 3 | 2 | 2 | 2 | 4 | 1 | 3 | 2 | 1 | 2 | 6 | 2 | 2 | 2 | 1 | 6 | 2 | 2 |
| CTC | 577 | 4 | 3 | 13 | 3 | 4 | 1 | 1 | 4 | 8 | 7 | 2 | 7 | 4 | 8 | 9 | 5 | 4 | 6 | 8 | 9 | 2 | 2 | 7 | 5 | 5 | 3 | 3 | 1 | 4 | 5 | 2 | 2 | 3 |  | 3 | 3 | 7 | 4 | 2 | 6 | 6 | 1 | 1 | 2 | 2 | 6 | 5 | 2 | 1 | 7 | 2 | 1 | 4 | 4 | 5 | 7 | 6 | 2 | 4 | 2 | 7 | 6 | 5 | 3 |
| CTG | 412 | 3 | 1 | 8 |  | 6 | 5 | 1 | 5 | 2 | 5 | 3 | 1 | 4 | 4 | 5 | 1 | 3 | 2 | 2 |  | 2 | 3 | 6 | 2 | 7 | 4 | 1 | 1 | 5 | 6 | 3 | 1 | 4 | 3 | 4 | 1 | 2 | 2 | 3 | 2 | 5 | 5 | 1 | 4 | 2 | 7 | 3 | 3 |  | 3 | 2 | 4 | 3 | 6 | 5 | 8 | 8 | 4 | 3 | 4 | 2 | 6 | 4 | 3 |
| CTT | 560 | 4 | 5 | 9 | 4 | 5 | 3 | 4 | 2 | 2 | 9 | 3 | 4 | 1 | 6 | 6 | 1 | 9 | 4 | 2 | 6 | 9 | 6 | 3 | 2 | 4 | 3 | 4 | 4 | 5 | 2 | 5 | 5 | 5 | 7 | 2 | 1 | 4 | 5 | 5 | 7 | 3 | 6 | 3 | 7 | 4 | 5 | 4 | 1 | 2 | 2 | 1 | 3 | 8 | 2 | 7 | 5 | 3 | 5 | 7 | 5 | 2 | 9 | 14 | 5 |
| GAA | 772 | 7 | 8 | 12 | 5 | 5 | 5 | 6 | 4 | 3 | 4 | 7 | 2 | 4 | 10 | 12 | 2 | 4 | 4 | 7 | 5 | 6 | 4 | 9 | 4 | 6 | 4 | 5 | 5 | 4 | 6 | 5 | 8 | 5 | 4 | 11 | 7 | 5 | 3 | 7 | 3 | 6 | 2 | 4 | 7 | 2 | 8 | 5 | 8 | 3 | 3 | 2 | 6 | 10 | 9 | 2 | 8 | 6 | 6 | 12 | 7 | 4 | 7 | 4 | 5 |
| GAC | 729 | 4 | 6 | 6 | 4 | 7 | 8 | 8 | 3 | 6 | 11 | 6 | 4 | 7 | 7 | 6 | 3 | 9 | 2 | 8 | 4 | 4 | 2 | 6 | 4 | 4 | 5 | 4 | 3 | 3 | 7 | 10 | 6 | 7 | 3 | 4 | 8 | 2 | 7 | 8 | 11 | 7 | 2 | 6 | 6 | 1 | 2 | 6 | 4 | 1 | 1 | 2 | 6 | 14 | 8 | 8 | 6 | 4 | 4 | 5 | 2 | 3 | 7 | 7 | 3 |
| GAG | 901 | 4 | 6 | 9 | 4 | 5 | 7 | 3 | 7 | 11 | 6 | 8 | 6 | 12 | 10 | 3 | 6 | 5 | 5 | 12 | 4 | 17 | 4 | 6 | 4 | 8 | 5 | 6 | 2 | 10 | 8 | 7 | 4 | 6 | 9 | 4 | 8 | 4 | 3 | 9 | 6 | 8 | 5 | 7 | 8 | 2 | 7 | 6 | 8 | 4 | 6 | 3 | 3 | 13 | 6 | 11 | 4 | 3 | 2 | 12 | 8 | 1 | 9 | 10 | 3 |
| GAT | 850 | 5 | 5 | 18 | 5 | 6 | 11 | 5 | 7 | 6 | 5 | 5 | 3 | 7 | 8 | 12 | 6 | 7 | 4 | 1 | 6 | 13 | 3 | 7 | 4 | 7 | 3 | 4 | 6 | 9 | 4 | 11 | 4 | 4 | 13 | 3 | 9 | 5 | 5 | 3 | 6 | 4 | 3 | 6 | 4 | 6 | 14 | 5 | 4 | 2 | 9 | 7 | 4 | 7 | 9 | 8 | 6 | 5 | 8 | 9 | 3 | 2 | 7 | 5 | 8 |
| GCA | 525 | 4 | 2 | 3 | 2 | 5 | 11 | 4 | 7 | 8 | 1 | 5 | 3 | 3 | 2 | 6 | 5 | 3 | 4 | 9 | 7 | 2 | 3 | 2 | 3 | 8 | 5 | 3 | 2 | 2 | 2 | 5 | 2 | 5 | 3 | 5 | 3 | 2 | 1 | 6 | 6 | 5 | 5 | 3 | 3 | 3 | 3 | 4 | 1 | 5 | 2 | 5 | 4 | 6 | 4 | 6 | 5 | 5 |  | 8 | 1 | 5 | 5 | 4 | 1 |
| GCC | 615 | 4 | 2 | 8 | 2 | 3 | 1 | 3 | 3 | 5 | 8 | 4 | 4 | 5 | 7 | 10 | 3 | 6 | 6 | 2 | 4 | 8 | 2 | 5 | 4 | 4 | 2 | 5 |  | 4 | 5 | 6 | 1 | 4 | 5 | 3 | 6 | 5 | 5 | 1 | 6 | 5 | 3 | 4 | 5 | 2 | 1 | 4 | 3 |  | 4 | 2 | 2 | 4 | 4 | 6 | 6 | 3 | 3 | 5 | 3 | 3 | 6 | 1 | 4 |
| GCG | 322 | 2 | 1 | 1 | 1 | 5 | 4 | 3 | 1 | 4 | 5 | 3 |  | 5 | 3 | 7 | 2 | 5 | 5 | 5 | 2 |  | 2 | 2 | 1 | 4 | 1 | 2 | 1 | 2 | 4 | 2 | 2 | 8 | 4 | 4 | 1 |  | 4 | 1 | 2 | 1 | 4 | 2 | 1 | 3 | 2 | 5 | 1 | 1 | 1 | 2 | 2 | 4 | 1 | 4 |  | 2 | 4 | 2 | 5 | 2 | 1 | 4 |  |
| GCT | 725 | 2 | 8 | 6 | 2 | 5 | 7 | 4 | 4 | 8 | 8 | 2 | 6 | 4 | 7 | 10 | 2 | 10 | 6 | 7 | 10 | 6 | 1 | 8 | 4 | 2 | 4 | 10 | 2 | 4 | 7 | 9 | 8 | 9 | 3 | 6 | 7 | 3 | 6 | 3 | 4 | 5 | 4 | 2 | 4 | 4 | 2 | 4 | 1 |  | 6 | 4 | 2 | 8 | 5 | 4 | 8 | 3 | 4 | 11 | 3 | 3 | 9 | 12 | 6 |
| GGA | 442 | 4 | 4 | 7 |  | 2 | 3 | 1 | 3 | 9 | 7 | 3 | 2 | 3 | 2 | 4 | 1 | 7 | 4 | 3 | 5 | 2 | 3 | 8 | 1 |  | 1 | 1 | 4 |  | 6 | 4 | 5 | 4 | 6 | 3 | 1 | 4 | 2 | 2 | 2 | 3 | 10 | 3 | 4 | 3 | 3 | 3 | 1 | 1 | 2 |  | 2 | 4 | 5 | 1 | 1 | 1 | 1 | 2 | 5 | 2 | 3 | 3 | 2 |
| GGC | 593 | 7 | 3 | 5 | 2 | 6 | 4 | 4 | 3 | 6 | 4 | 5 | 6 | 4 | 7 | 5 | 1 | 2 | 2 | 5 | 3 | 2 | 2 | 6 | 2 | 1 | 3 | 3 |  | 4 | 5 | 4 | 5 | 2 | 6 | 6 | 8 | 5 | 3 | 6 | 2 | 11 | 5 | 1 | 3 | 1 | 6 | 6 | 2 |  | 1 | 4 | 4 | 2 | 5 | 2 | 3 | 4 | 4 | 3 | 4 | 2 | 7 | 7 | 2 |
| GGG | 269 | 2 |  | 3 |  | 6 | 1 | 4 | 3 | 9 | 3 | 3 | 1 | 2 | 1 | 1 |  | 1 | 1 | 4 | 2 | 3 | 3 | 1 | 1 | 2 | 1 | 1 | 1 | 2 | 2 | 7 |  | 1 | 2 |  | 1 | 1 |  | 3 | 1 | 3 | 1 | 4 | 3 |  | 1 | 1 | 1 | 2 | 3 |  | 3 | 4 | 1 | 3 |  | 4 | 3 | 2 | 3 | 2 | 5 | 4 | 2 |
| GGT | 499 | 3 | 4 | 3 | 1 | 2 | 4 | 2 | 2 | 4 | 2 | 7 | 4 | 2 | 6 | 5 | 2 | 3 | 4 | 6 | 4 | 5 | 5 | 2 | 5 | 4 | 1 | 3 | 1 | 1 | 8 | 3 | 3 | 3 | 4 | 3 | 3 | 4 | 3 | 2 |  | 3 | 2 | 1 | 1 |  | 2 | 3 | 1 |  | 4 | 1 | 3 | 5 | 6 | 2 | 3 | 1 | 5 | 7 | 3 |  | 2 | 3 | 2 |
| GTA | 243 | 1 |  |  |  | 5 | 1 | 2 | 3 | 5 | 1 | 1 |  | 1 | 2 | 4 | 4 | 2 | 2 | 3 | 1 | 3 | 1 | 1 |  | 1 | 2 | 1 | 7 | 2 | 2 | 3 | 3 | 2 | 1 | 4 | 2 | 2 | 1 | 1 |  | 1 | 1 | 1 | 2 |  | 1 | 4 |  | 1 | 2 | 1 | 3 | 1 |  | 1 | 2 | 2 | 3 | 2 | 1 | 1 |  | 1 | 2 |
| GTC | 579 | 2 | 6 | 8 | 2 | 3 | 4 | 1 | 7 | 10 | 5 | 1 | 1 | 4 | 6 | 5 | 4 | 8 | 3 | 7 |  | 2 | 1 | 3 | 3 | 2 | 4 |  | 3 | 5 | 5 | 7 | 6 | 3 | 7 | 4 | 6 | 6 | 4 | 6 | 3 | 8 | 9 | 4 | 6 | 3 | 1 | 2 | 3 | 2 | 3 | 2 | 5 | 4 | 10 | 7 | 5 | 5 | 5 | 3 | 5 | 2 | 5 | 9 | 4 |
| GTG | 335 |  | 2 | 7 | 2 | 2 |  | 3 | 1 | 4 | 3 | 3 | 1 | 1 | 2 | 6 | 2 | 2 | 4 | 1 | 3 | 1 | 2 | 4 | 2 | 2 |  | 4 | 1 | 4 | 3 | 1 | 1 | 1 | 1 |  | 2 |  | 4 |  | 2 | 1 | 3 | 1 |  | 2 | 1 | 2 | 1 | 1 | 4 | 2 | 1 | 5 | 3 | 2 | 1 | 2 |  | 3 | 2 | 1 | 1 | 3 | 3 |
| GTT | 466 | 1 | 6 | 9 | 3 | 4 | 5 | 3 | 3 | 4 | 5 | 5 | 2 | 6 | 2 | 8 | 4 | 2 | 1 | 7 | 1 | 2 | 4 | 1 | 2 | 5 | 2 | 2 | 2 | 4 | 4 | 2 | 6 | 3 | 1 | 2 | 1 | 2 | 1 | 1 | 2 | 4 | 4 | 1 |  | 1 | 3 | 4 | 4 | 1 | 5 |  | 2 | 4 | 5 | 5 | 4 | 2 | 4 | 7 | 2 | 5 | 4 | 7 | 4 |
| TAA | 25 |  |  |  |  |  |  |  |  |  |  |  |  |  |  |  |  |  |  |  |  |  |  |  |  |  |  |  |  |  |  |  |  |  |  |  |  |  |  |  |  |  |  | 2 |  |  |  |  |  |  |  |  |  |  |  |  |  |  |  |  |  |  |  |  |  |
| TAC | 384 | 2 | 5 | 7 | 3 | 7 | 5 | 5 | 2 | 5 | 5 | 2 | 3 | 4 | 7 | 3 | 3 | 7 | 4 | 2 | 3 | 1 | 2 | 6 | 3 | 2 |  | 4 | 3 | 2 | 4 | 5 | 1 | 7 | 6 | 1 | 2 | 3 | 4 | 1 |  | 3 | 6 | 5 | 2 | 4 | 3 | 1 | 2 | 4 | 1 | 3 | 4 | 6 | 3 | 3 | 5 | 4 | 3 | 5 | 4 |  | 4 | 9 | 1 |
| TAG | 23 |  |  |  |  |  |  |  |  |  |  |  |  |  |  |  |  |  |  |  |  |  |  |  |  |  |  |  |  |  |  |  |  |  |  |  |  |  |  |  |  |  |  |  |  |  |  |  |  |  |  |  |  |  |  |  |  |  |  |  |  |  |  |  |  |
| TAT | 351 | 4 | 3 | 5 | 2 | 2 | 2 | 1 | 1 | 5 | 1 | 9 | 1 | 7 | 2 | 3 | 3 | 3 | 3 | 1 | 3 | 3 | 1 | 2 | 1 | 4 | 1 | 3 | 2 | 3 | 3 | 3 | 1 | 5 | 5 | 3 | 3 | 2 | 2 | 2 | 1 | 2 | 2 | 1 | 3 | 1 | 1 | 2 | 5 |  |  | 2 |  | 5 | 1 | 6 |  | 2 | 3 | 5 | 1 | 1 | 3 | 5 | 6 |
| TCA | 404 | 3 | 5 | 8 |  | 3 | 4 | 3 | 2 | 4 | 5 | 3 | 1 |  | 5 | 2 | 1 | 3 | 3 | 5 | 1 | 3 | 1 | 1 | 1 | 5 | 4 | 4 | 2 | 2 | 4 | 3 | 2 | 6 | 1 | 3 | 2 | 2 | 2 |  | 5 | 5 | 1 | 3 | 1 | 2 | 1 | 4 | 5 | 1 | 3 | 1 | 3 | 4 | 4 | 2 | 5 | 2 | 1 | 4 | 3 | 4 | 3 | 7 | 3 |
| TCC | 369 | 1 | 1 | 8 | 1 | 2 | 4 | 3 | 6 | 7 | 2 | 7 | 2 | 2 | 5 | 3 | 2 | 5 | 2 | 4 | 7 | 1 | 1 | 1 | 2 |  | 4 | 1 |  | 1 | 1 | 6 | 4 | 3 | 2 |  | 2 | 3 | 5 | 4 |  | 5 | 2 | 2 |  | 2 | 2 | 3 | 1 | 2 | 4 | 4 | 1 | 4 | 4 | 5 | 2 | 4 | 4 | 1 | 4 | 2 | 1 | 5 | 2 |
| TCG | 290 | 5 | 1 | 5 | 2 |  |  | 1 | 2 | 3 | 4 | 1 | 1 | 3 | 2 | 2 | 1 | 4 | 2 |  | 4 | 5 | 2 | 1 | 2 | 4 | 1 | 2 | 2 | 2 | 2 | 5 |  |  | 3 |  | 2 | 1 |  | 4 | 4 | 2 | 2 | 2 | 1 | 3 | 1 |  | 1 |  | 1 | 2 | 1 | 2 | 3 | 4 | 4 | 2 | 2 | 5 | 3 | 2 | 1 | 4 | 3 |
| TCT | 496 | 2 | 5 | 6 |  | 4 | 7 | 3 | 5 | 2 | 4 | 5 | 3 | 2 | 2 | 4 | 2 | 9 | 5 | 6 | 4 | 5 | 4 | 1 | 1 | 4 | 2 |  |  | 2 | 4 | 3 | 2 | 7 | 6 | 1 | 4 | 3 | 3 | 1 | 5 | 1 | 9 | 1 | 4 | 1 | 3 | 4 | 4 |  | 6 |  | 3 | 3 | 3 | 8 | 7 | 3 | 7 | 4 | 4 | 3 | 3 | 3 | 3 |
| TGA | 20 |  |  |  |  |  |  |  | 1 |  |  |  |  |  |  |  |  |  |  |  |  |  |  |  |  |  |  |  |  |  |  |  |  |  |  |  |  |  |  |  |  |  |  |  |  |  |  |  |  |  |  |  |  |  |  |  |  |  |  |  |  |  |  |  |  |
| TGC | 246 | 1 | 1 | 3 | 2 | 1 | 2 | 2 | 2 | 5 | 3 | 2 | 2 |  | 3 | 5 |  | 1 | 2 |  | 3 | 5 | 1 |  | 2 | 3 | 1 |  | 1 |  | 2 | 5 |  | 2 | 1 | 2 | 1 | 1 | 1 |  | 2 | 1 |  |  | 3 |  | 4 | 1 | 1 |  | 2 | 2 |  | 2 |  | 1 | 4 | 4 | 2 | 5 | 1 | 2 | 3 | 2 | 2 |
| TGG | 404 | 3 | 6 | 2 | 5 | 3 | 1 | 2 | 6 | 3 | 3 | 4 | 2 |  | 3 | 5 | 2 | 5 | 2 | 5 | 4 | 6 | 3 | 1 | 3 | 4 | 3 |  | 4 | 5 | 2 | 3 | 5 | 4 | 3 | 1 | 6 | 3 | 3 | 2 | 3 | 4 | 3 | 4 | 3 | 2 | 3 | 3 | 2 | 2 | 4 | 3 | 3 | 10 | 2 | 4 | 4 | 3 | 7 | 8 | 1 | 4 | 5 | 4 | 1 |
| TGT | 174 |  | 1 | 2 | 1 |  | 1 | 3 | 5 |  |  |  | 1 |  | 4 | 2 | 1 |  | 2 |  | 1 | 1 |  | 1 |  | 2 | 2 | 1 |  |  | 1 | 3 | 3 | 1 | 1 | 2 | 1 | 2 | 3 | 5 |  |  |  |  | 2 |  |  | 1 | 1 | 1 | 1 | 2 | 2 | 4 | 1 | 2 | 1 | 1 | 1 | 2 |  |  | 3 | 4 |  |
| TTA | 184 | 3 | 2 | 1 | 2 | 1 | 2 |  | 1 | 2 | 1 |  |  | 1 | 2 | 1 | 1 | 2 | 1 | 1 | 1 | 4 | 3 | 1 | 2 | 3 | 1 |  | 2 | 3 | 3 |  | 1 | 2 | 3 | 1 | 2 | 3 |  | 3 | 2 | 2 | 1 | 1 |  | 2 | 1 |  | 2 | 1 | 1 | 2 | 2 | 2 | 1 | 1 | 2 | 2 | 1 | 1 |  | 1 | 3 | 4 |  |
| TTC | 532 | 6 | 2 | 7 |  | 5 | 5 | 8 | 2 | 3 | 4 | 7 | 3 | 3 | 6 | 7 | 4 | 4 | 3 | 4 | 1 | 4 | 3 | 6 | 6 | 4 | 4 | 1 | 1 | 2 | 3 | 8 | 4 | 4 | 6 | 3 | 3 | 3 | 5 | 1 | 3 | 4 | 4 | 4 | 1 | 3 | 3 | 5 | 4 | 1 | 2 | 1 | 2 | 7 | 6 | 5 | 5 | 9 | 6 | 6 | 2 | 1 | 4 | 11 | 3 |
| TTG | 436 | 2 | 3 | 5 | 2 | 4 | 3 |  |  | 3 | 2 |  | 1 | 8 | 6 | 2 | 2 | 4 | 3 | 2 | 3 | 5 | 2 | 3 | 3 | 1 | 2 | 3 | 1 | 2 | 4 | 5 | 2 | 2 | 5 | 1 | 5 |  | 5 | 4 | 3 | 1 | 6 | 3 | 2 |  | 3 | 2 | 2 | 3 | 1 | 2 | 1 | 5 | 3 | 5 | 5 | 5 | 2 | 5 | 2 | 1 | 6 | 6 | 2 |
| TTT | 419 | 2 | 1 | 6 | 1 | 6 | 2 | 4 |  | 5 | 2 | 5 | 4 | 4 | 7 | 10 | 2 | 2 | 5 | 6 | 4 | 4 | 4 | 2 | 3 | 3 | 2 | 5 | 2 | 7 | 3 | 8 | 3 | 6 | 1 | 3 | 5 | 4 | 4 | 2 | 8 | 3 | 5 | 2 | 2 | 2 | 2 | 3 | 5 |  | 5 |  | 3 | 10 | 7 | 3 | 7 | 6 | 2 | 6 | 4 | 1 | 5 | 4 | 4 |


---


 **Amino acid changes**

How to read this table:   
- Rows are reference amino acids and columns are changed amino acids. E.g. Row 'A' column 'E' indicates how many 'A' amino acids have been replaced by 'E' amino acids.  
- Red background colors indicate that more changes happened (heat-map).  
- Diagonals are indicated using grey background color   
- WARNING: This table may include different translation codon tables (e.g. mamalian DNA and mitochondrial DNA).

|  | \* | - | ? | A | C | D | E | F | G | H | I | K | L | M | N | P | Q | R | S | T | V | W | Y |
| --- | --- | --- | --- | --- | --- | --- | --- | --- | --- | --- | --- | --- | --- | --- | --- | --- | --- | --- | --- | --- | --- | --- | --- |
| \* |  | 68 |  |  |  |  |  |  | 2 |  |  |  |  |  |  |  |  |  |  | 1 |  |  |  |
| - | 12 |  | 2 | 178 | 35 | 191 | 133 | 94 | 194 | 42 | 121 | 115 | 242 | 46 | 99 | 130 | 83 | 113 | 228 | 154 | 143 | 51 | 87 |
| ? |  |  |  |  |  |  |  |  |  |  |  |  |  |  |  |  |  |  |  |  |  |  |  |
| A | 32 | 2,186 | 1 | 55 | 23 | 32 | 44 | 32 | 56 | 44 | 48 | 30 | 99 | 33 | 20 | 53 | 47 | 94 | 110 | 70 | 43 | 26 | 23 |
| C | 10 | 419 | 1 | 14 | 4 | 4 | 7 | 8 | 6 | 8 | 8 | 6 | 22 | 7 | 5 | 10 | 1 | 17 | 21 | 16 | 8 | 7 | 5 |
| D | 21 | 1,578 | 1 | 47 | 17 | 33 | 18 | 25 | 38 | 16 | 38 | 33 | 71 | 18 | 20 | 43 | 25 | 59 | 89 | 55 | 42 | 14 | 20 |
| E | 21 | 1,672 | 1 | 40 | 23 | 28 | 26 | 24 | 47 | 18 | 44 | 32 | 71 | 15 | 23 | 54 | 28 | 70 | 81 | 42 | 46 | 24 | 18 |
| F | 17 | 948 | 3 | 30 | 14 | 15 | 16 | 16 | 25 | 13 | 26 | 21 | 55 | 17 | 4 | 32 | 16 | 42 | 63 | 32 | 27 | 12 | 12 |
| G | 18 | 1,801 | 2 | 40 | 28 | 31 | 22 | 25 | 58 | 25 | 31 | 34 | 82 | 15 | 14 | 51 | 31 | 73 | 76 | 50 | 34 | 14 | 22 |
| H | 10 | 588 | 1 | 19 | 8 | 10 | 10 | 19 | 11 | 10 | 19 | 10 | 39 | 5 | 12 | 22 | 8 | 30 | 36 | 20 | 15 | 8 | 10 |
| I | 22 | 1,420 | 6 | 36 | 17 | 20 | 24 | 19 | 36 | 18 | 29 | 30 | 79 | 18 | 23 | 41 | 26 | 70 | 73 | 59 | 43 | 19 | 24 |
| K | 14 | 1,168 | 4 | 37 | 16 | 14 | 18 | 22 | 23 | 16 | 34 | 24 | 59 | 14 | 18 | 35 | 18 | 53 | 53 | 35 | 29 | 14 | 18 |
| L | 52 | 2,420 | 2 | 74 | 31 | 32 | 27 | 51 | 73 | 40 | 63 | 54 | 120 | 30 | 30 | 80 | 43 | 100 | 138 | 60 | 66 | 22 | 28 |
| M | 11 | 537 | 1 | 7 | 8 | 6 | 4 | 7 | 14 | 8 | 12 | 13 | 32 | 5 | 11 | 11 | 13 | 17 | 42 | 14 | 7 | 4 | 12 |
| N | 18 | 944 |  | 29 | 3 | 13 | 16 | 18 | 33 | 15 | 23 | 16 | 42 | 12 | 23 | 31 | 19 | 38 | 51 | 43 | 26 | 10 | 11 |
| P | 20 | 1,514 | 1 | 31 | 28 | 25 | 21 | 30 | 43 | 28 | 34 | 30 | 73 | 21 | 16 | 59 | 27 | 54 | 61 | 42 | 25 | 15 | 22 |
| Q | 22 | 1,065 | 2 | 26 | 12 | 10 | 17 | 24 | 32 | 17 | 25 | 16 | 53 | 13 | 10 | 27 | 27 | 44 | 50 | 33 | 22 | 13 | 15 |
| R | 28 | 1,444 | 1 | 32 | 24 | 25 | 27 | 24 | 39 | 25 | 40 | 21 | 63 | 15 | 15 | 48 | 34 | 67 | 85 | 38 | 41 | 15 | 16 |
| S | 30 | 2,280 |  | 62 | 41 | 30 | 32 | 27 | 56 | 41 | 43 | 45 | 106 | 16 | 31 | 51 | 52 | 94 | 131 | 72 | 51 | 19 | 35 |
| T | 23 | 1,969 | 1 | 50 | 21 | 11 | 18 | 23 | 44 | 25 | 39 | 35 | 78 | 24 | 24 | 51 | 31 | 72 | 90 | 64 | 44 | 12 | 23 |
| V | 21 | 1,621 | 2 | 35 | 22 | 21 | 19 | 23 | 46 | 15 | 38 | 28 | 87 | 23 | 21 | 32 | 32 | 71 | 77 | 47 | 32 | 15 | 25 |
| W | 8 | 404 |  | 11 | 8 | 9 | 5 | 6 | 14 | 6 | 5 | 5 | 23 | 5 | 11 | 13 | 10 | 18 | 25 | 12 | 10 | 8 | 7 |
| Y | 15 | 732 | 3 | 15 | 11 | 16 | 16 | 14 | 24 | 13 | 26 | 18 | 37 | 6 | 13 | 19 | 13 | 40 | 39 | 25 | 19 | 10 | 5 |


---


 **Variants by chromosome**

```
		  

		FSP34_Chr01, Position,0,100000,200000,300000,400000,500000,600000,700000,800000,900000,1000000,1100000,1200000,1300000,1400000,1500000,1600000,1700000,1800000,1900000,2000000,2100000,2200000,2300000,2400000,2500000,2600000,2700000,2800000,2900000,3000000,3100000,3200000,3300000,3400000,3500000,3600000,3700000,3800000,3900000,4000000,4100000,4200000,4300000,4400000,4500000,4600000,4700000,4800000,4900000,5000000,5100000,5200000,5300000,5400000,5500000,5600000,5700000,5800000,5900000,6000000,6100000,6200000,6300000,6400000
FSP34_Chr01,Count,52,4,12,11,6,5,2,3,3,2,1,0,1,3,0,0,4,0,0,0,2,1,1,2,5,0,1,1,5,0,0,0,0,1,4,4,1,2,3,3,1,3,2,0,2,4,0,2,0,2,1,1,0,2,0,2,1,2,2,4,8,10,6,13,1

	
```

```
		  

		FSP34_Chr02, Position,0,100000,200000,300000,400000,500000,600000,700000,800000,900000,1000000,1100000,1200000,1300000,1400000,1500000,1600000,1700000,1800000,1900000,2000000,2100000,2200000,2300000,2400000,2500000,2600000,2700000,2800000,2900000,3000000,3100000,3200000,3300000,3400000,3500000,3600000,3700000,3800000,3900000,4000000,4100000,4200000,4300000,4400000,4500000,4600000,4700000,4800000,4900000,5000000
FSP34_Chr02,Count,2,12,7,3,9,5,3,1,3,1,0,0,3,4,1,1,2,8,1,1,0,0,8,0,2,7,0,0,1,1,4,2,0,3,3,1,2,3,3,3,1,3,7,6,7,3,4,4,10,7,36

	
```

```
		  

		FSP34_Chr03, Position,0,100000,200000,300000,400000,500000,600000,700000,800000,900000,1000000,1100000,1200000,1300000,1400000,1500000,1600000,1700000,1800000,1900000,2000000,2100000,2200000,2300000,2400000,2500000,2600000,2700000,2800000,2900000,3000000,3100000,3200000,3300000,3400000,3500000,3600000,3700000,3800000,3900000,4000000,4100000,4200000,4300000,4400000,4500000,4600000,4700000,4800000,4900000,5000000
FSP34_Chr03,Count,4,3,31,19,5,2,4,3,3,8,2,4,2,2,2,2,2,1,1,0,2,2,1,4,2,0,6,4,1,2,2,1,0,0,0,1,1,2,2,1,3,1,7,0,1,4,4,6,6,7,15

	
```

```
		  

		FSP34_Chr04, Position,0,100000,200000,300000,400000,500000,600000,700000,800000,900000,1000000,1100000,1200000,1300000,1400000,1500000,1600000,1700000,1800000,1900000,2000000,2100000,2200000,2300000,2400000,2500000,2600000,2700000,2800000,2900000,3000000,3100000,3200000,3300000,3400000,3500000,3600000,3700000,3800000,3900000,4000000,4100000,4200000,4300000
FSP34_Chr04,Count,18,5,2,7,5,5,1,2,0,1,2,0,1,0,0,1,1,0,1,0,0,3,0,2,3,0,7,0,1,3,3,1,3,0,12,4,8,4,9,4,8,14,6,0

	
```

```
		  

		FSP34_Chr05, Position,0,100000,200000,300000,400000,500000,600000,700000,800000,900000,1000000,1100000,1200000,1300000,1400000,1500000,1600000,1700000,1800000,1900000,2000000,2100000,2200000,2300000,2400000,2500000,2600000,2700000,2800000,2900000,3000000,3100000,3200000,3300000,3400000,3500000,3600000,3700000,3800000,3900000,4000000,4100000,4200000,4300000,4400000
FSP34_Chr05,Count,8,6,7,12,2,6,4,5,0,1,2,4,0,0,0,1,1,4,0,2,2,1,3,1,0,1,0,4,0,1,1,1,1,0,0,1,2,2,3,0,4,3,8,60,19

	
```

```
		  

		FSP34_Chr06, Position,0,100000,200000,300000,400000,500000,600000,700000,800000,900000,1000000,1100000,1200000,1300000,1400000,1500000,1600000,1700000,1800000,1900000,2000000,2100000,2200000,2300000,2400000,2500000,2600000,2700000,2800000,2900000,3000000,3100000,3200000,3300000,3400000,3500000,3600000,3700000,3800000,3900000,4000000,4100000,4200000,4300000
FSP34_Chr06,Count,46,2,9,5,7,11,1,7,1,2,1,0,2,0,1,2,0,1,3,2,1,3,2,1,1,2,2,0,3,0,2,1,2,2,6,7,9,12,4,11,11,7,5,0

	
```

```
		  

		FSP34_Chr07, Position,0,100000,200000,300000,400000,500000,600000,700000,800000,900000,1000000,1100000,1200000,1300000,1400000,1500000,1600000,1700000,1800000,1900000,2000000,2100000,2200000,2300000,2400000,2500000,2600000,2700000,2800000,2900000,3000000,3100000,3200000,3300000,3400000,3500000
FSP34_Chr07,Count,7,0,8,17,6,2,2,5,4,3,0,1,0,2,1,2,1,3,1,0,5,2,0,1,2,2,6,4,3,3,1,10,7,4,15,14

	
```

```
		  

		FSP34_Chr08, Position,0,100000,200000,300000,400000,500000,600000,700000,800000,900000,1000000,1100000,1200000,1300000,1400000,1500000,1600000,1700000,1800000,1900000,2000000,2100000,2200000,2300000,2400000,2500000,2600000,2700000,2800000,2900000,3000000,3100000
FSP34_Chr08,Count,48,6,10,10,10,5,3,1,1,3,2,0,0,2,1,1,3,1,1,9,5,2,1,7,2,3,4,7,2,2,3,4

	
```

```
		  

		FSP34_Chr09, Position,0,10000,20000,30000,40000,50000,60000,70000,80000,90000,100000,110000,120000,130000,140000,150000,160000,170000,180000,190000,200000,210000,220000,230000,240000,250000,260000,270000,280000,290000,300000,310000,320000,330000,340000,350000,360000,370000,380000,390000,400000,410000,420000,430000,440000,450000,460000,470000,480000,490000,500000,510000,520000,530000,540000,550000,560000,570000,580000,590000,600000,610000,620000,630000,640000,650000,660000,670000,680000,690000,700000,710000,720000,730000,740000,750000,760000,770000,780000,790000,800000,810000,820000,830000,840000,850000,860000,870000,880000,890000,900000,910000,920000,930000,940000,950000,960000,970000,980000,990000,1000000,1010000,1020000,1030000,1040000,1050000,1060000,1070000,1080000,1090000,1100000,1110000,1120000,1130000,1140000,1150000,1160000,1170000,1180000,1190000,1200000,1210000,1220000,1230000,1240000,1250000,1260000,1270000,1280000,1290000,1300000,1310000,1320000,1330000,1340000,1350000,1360000,1370000,1380000,1390000,1400000,1410000,1420000,1430000,1440000,1450000,1460000,1470000,1480000,1490000,1500000,1510000,1520000,1530000,1540000,1550000,1560000,1570000,1580000,1590000,1600000,1610000,1620000,1630000,1640000,1650000,1660000,1670000,1680000,1690000,1700000,1710000,1720000,1730000,1740000,1750000,1760000,1770000,1780000,1790000,1800000,1810000,1820000,1830000,1840000,1850000,1860000,1870000,1880000,1890000,1900000,1910000,1920000,1930000,1940000,1950000,1960000,1970000,1980000,1990000,2000000,2010000,2020000,2030000,2040000,2050000,2060000,2070000,2080000,2090000,2100000,2110000,2120000,2130000,2140000,2150000,2160000,2170000,2180000,2190000,2200000,2210000,2220000,2230000,2240000,2250000,2260000,2270000,2280000,2290000,2300000,2310000,2320000,2330000,2340000,2350000,2360000,2370000,2380000,2390000,2400000,2410000,2420000,2430000,2440000,2450000,2460000,2470000,2480000,2490000,2500000,2510000,2520000,2530000,2540000,2550000,2560000,2570000,2580000,2590000,2600000,2610000,2620000,2630000,2640000,2650000,2660000,2670000,2680000,2690000,2700000,2710000,2720000,2730000,2740000,2750000,2760000,2770000,2780000,2790000,2800000,2810000,2820000,2830000,2840000,2850000,2860000,2870000,2880000,2890000,2900000,2910000,2920000,2930000,2940000,2950000,2960000,2970000,2980000
FSP34_Chr09,Count,3,12,0,0,0,1,0,0,0,2,0,0,0,0,0,0,0,0,0,0,0,0,0,0,0,1,2,1,0,0,0,0,0,0,1,0,0,2,0,0,0,0,0,0,1,1,0,0,0,1,0,0,0,0,0,1,1,1,0,1,0,0,0,0,0,0,0,0,0,0,0,0,0,0,0,1,0,0,0,0,1,0,0,1,0,0,1,1,0,0,1,0,0,0,0,0,0,0,0,0,1,0,0,1,0,0,0,0,1,0,1,2,0,0,0,0,0,1,1,0,0,0,0,0,0,0,0,0,0,0,0,0,0,0,0,0,5,1,2,2,0,0,0,0,0,0,0,2,0,0,0,0,0,0,0,0,0,0,0,0,0,0,0,0,1,1,1,0,0,0,0,0,0,1,0,1,0,0,0,0,0,0,0,0,0,0,0,0,0,0,0,0,0,0,0,1,0,0,0,0,0,0,0,0,0,0,0,0,1,1,1,0,0,0,0,0,0,0,0,1,0,0,0,0,0,0,0,0,1,0,0,0,2,0,0,0,1,0,0,0,0,0,0,0,0,0,0,0,0,0,1,0,0,1,0,0,0,0,2,1,0,0,0,1,1,0,0,0,1,0,0,0,0,0,0,0,0,2,1,0,0,0,1,1,0,1,2,1,0,0,0,0,0,1,6,5,5,1,0

	
```

```
		  

		FSP34_Chr10, Position,0,10000,20000,30000,40000,50000,60000,70000,80000,90000,100000,110000,120000,130000,140000,150000,160000,170000,180000,190000,200000,210000,220000,230000,240000,250000,260000,270000,280000,290000,300000,310000,320000,330000,340000,350000,360000,370000,380000,390000,400000,410000,420000,430000,440000,450000,460000,470000,480000,490000,500000,510000,520000,530000,540000,550000,560000,570000,580000,590000,600000,610000,620000,630000,640000,650000,660000,670000,680000,690000,700000,710000,720000,730000,740000,750000,760000,770000,780000,790000,800000,810000,820000,830000,840000,850000,860000,870000,880000,890000,900000,910000,920000,930000,940000,950000,960000,970000,980000,990000,1000000,1010000,1020000,1030000,1040000,1050000,1060000,1070000,1080000,1090000,1100000,1110000,1120000,1130000,1140000,1150000,1160000,1170000,1180000,1190000,1200000,1210000,1220000,1230000,1240000,1250000,1260000,1270000,1280000,1290000,1300000,1310000,1320000,1330000,1340000,1350000,1360000,1370000,1380000,1390000,1400000,1410000,1420000,1430000,1440000,1450000,1460000,1470000,1480000,1490000,1500000,1510000,1520000,1530000,1540000,1550000,1560000,1570000,1580000,1590000,1600000,1610000,1620000,1630000,1640000,1650000,1660000,1670000,1680000,1690000,1700000,1710000,1720000,1730000,1740000,1750000,1760000,1770000,1780000,1790000,1800000,1810000,1820000,1830000,1840000,1850000,1860000,1870000,1880000,1890000,1900000,1910000,1920000,1930000,1940000,1950000,1960000,1970000,1980000,1990000,2000000,2010000,2020000,2030000,2040000,2050000,2060000,2070000,2080000,2090000,2100000,2110000,2120000,2130000,2140000,2150000,2160000,2170000,2180000,2190000,2200000,2210000,2220000,2230000,2240000,2250000,2260000,2270000,2280000,2290000,2300000,2310000,2320000,2330000,2340000,2350000,2360000,2370000,2380000,2390000,2400000,2410000,2420000,2430000,2440000,2450000,2460000,2470000,2480000,2490000,2500000,2510000,2520000,2530000,2540000,2550000,2560000,2570000,2580000,2590000,2600000,2610000,2620000,2630000,2640000,2650000,2660000,2670000,2680000,2690000
FSP34_Chr10,Count,0,0,0,2,2,3,1,0,0,0,0,2,0,1,1,1,0,1,2,4,1,1,0,0,1,1,1,0,0,0,2,0,0,1,2,0,1,1,1,0,2,0,0,0,0,0,0,0,0,0,0,0,0,0,0,0,0,0,1,0,0,2,0,0,0,0,0,1,0,0,0,0,0,0,0,0,2,1,0,0,1,1,0,0,1,0,0,0,0,0,0,0,0,1,0,0,0,0,0,0,0,1,0,0,0,1,0,0,0,0,1,1,1,0,0,1,1,0,0,0,0,0,1,0,0,0,0,0,0,0,1,0,0,0,0,1,0,0,0,0,0,0,0,0,0,0,0,0,0,0,0,0,0,1,0,0,0,0,0,0,0,0,0,0,0,0,0,0,0,0,0,0,0,0,0,0,0,0,1,2,0,0,1,1,0,0,0,1,0,0,0,0,0,0,0,0,1,0,0,0,1,0,0,0,0,1,0,1,1,1,0,1,1,0,1,0,0,0,1,1,2,0,0,1,0,0,0,0,1,2,0,0,1,0,0,0,1,0,1,0,0,0,1,1,0,1,0,0,1,0,1,0,0,0,1,0,1,0,0,1,4,2,1,0,0,0,0,2,2,1

	
```

```
		  

		FSP34_Chr11, Position,0,10000,20000,30000,40000,50000,60000,70000,80000,90000,100000,110000,120000,130000,140000,150000,160000,170000,180000,190000,200000,210000,220000,230000,240000,250000,260000,270000,280000,290000,300000,310000,320000,330000,340000,350000,360000,370000,380000,390000,400000,410000,420000,430000,440000,450000,460000,470000,480000,490000,500000,510000,520000,530000,540000,550000,560000,570000,580000,590000,600000,610000,620000,630000,640000,650000,660000,670000,680000,690000,700000,710000,720000,730000,740000,750000,760000,770000,780000,790000,800000,810000,820000,830000,840000,850000,860000,870000,880000,890000,900000,910000,920000,930000,940000,950000,960000,970000,980000,990000,1000000,1010000,1020000,1030000,1040000,1050000,1060000,1070000,1080000,1090000,1100000,1110000,1120000,1130000,1140000,1150000,1160000,1170000,1180000,1190000,1200000,1210000,1220000,1230000,1240000,1250000,1260000,1270000,1280000,1290000,1300000,1310000,1320000,1330000,1340000,1350000,1360000,1370000,1380000,1390000,1400000,1410000,1420000,1430000,1440000,1450000,1460000,1470000,1480000,1490000,1500000,1510000,1520000,1530000,1540000,1550000,1560000,1570000,1580000,1590000,1600000,1610000,1620000,1630000,1640000,1650000,1660000,1670000,1680000,1690000,1700000,1710000,1720000,1730000,1740000,1750000,1760000,1770000,1780000,1790000,1800000,1810000,1820000,1830000,1840000,1850000,1860000,1870000,1880000,1890000,1900000,1910000,1920000,1930000,1940000,1950000,1960000,1970000,1980000,1990000,2000000,2010000,2020000,2030000,2040000,2050000,2060000,2070000,2080000,2090000,2100000,2110000,2120000,2130000,2140000,2150000,2160000,2170000,2180000,2190000,2200000,2210000,2220000
FSP34_Chr11,Count,0,2,1,1,1,0,0,0,1,0,0,0,1,0,0,0,0,1,0,0,0,1,0,1,1,0,0,2,1,2,1,3,0,2,0,1,0,0,1,0,0,0,2,1,1,0,0,2,0,0,1,0,0,0,0,0,0,0,0,1,1,0,0,0,1,0,0,2,2,0,0,0,0,1,0,2,0,1,3,1,1,0,0,0,0,0,0,1,0,1,0,0,0,0,0,2,0,1,0,1,3,0,0,0,2,0,0,0,0,0,0,0,0,1,0,0,0,0,0,0,0,0,0,0,0,0,0,0,0,0,0,0,0,1,0,0,0,0,1,1,1,0,0,1,1,0,0,0,0,0,0,0,0,0,0,0,1,0,0,1,0,0,0,0,0,0,0,1,0,0,0,2,0,0,1,1,1,0,0,0,0,0,1,0,0,0,0,0,0,0,0,1,1,1,0,0,0,0,0,1,0,2,0,0,0,0,0,1,0,0,0,0,1,0,0,0,0,0,0,0,1,0,0

	
```

```
		  

		FSP34_Chr12, Position,0,10000,20000,30000,40000,50000,60000,70000,80000,90000,100000,110000,120000,130000,140000,150000,160000,170000,180000,190000,200000,210000,220000,230000,240000,250000,260000,270000,280000,290000,300000,310000,320000,330000,340000,350000,360000,370000,380000,390000,400000,410000,420000,430000,440000,450000,460000,470000,480000,490000,500000,510000,520000
FSP34_Chr12,Count,0,0,0,0,1,1,0,0,0,0,0,1,0,0,1,0,1,1,3,1,1,3,1,0,0,0,0,0,4,1,2,0,2,2,1,1,0,0,0,0,1,0,1,1,0,0,0,1,1,2,0,0,0

	
```

```
		  

		FSP34_Contig01, Position,0,1000,2000,3000,4000,5000,6000,7000,8000,9000,10000,11000,12000,13000,14000,15000,16000,17000,18000,19000,20000,21000,22000,23000,24000,25000,26000,27000,28000,29000,30000,31000,32000,33000,34000,35000,36000,37000,38000,39000,40000,41000,42000,43000,44000,45000,46000,47000,48000,49000,50000,51000,52000,53000,54000,55000,56000,57000,58000,59000,60000,61000,62000,63000,64000,65000,66000,67000,68000,69000,70000,71000,72000,73000,74000,75000,76000,77000,78000,79000,80000,81000,82000,83000,84000,85000
FSP34_Contig01,Count,0,0,0,0,0,0,0,0,0,0,0,0,1,0,0,0,0,0,0,1,0,0,1,0,0,0,0,0,0,0,0,0,0,0,0,0,0,0,0,0,0,0,0,0,0,0,0,0,0,0,0,0,0,0,0,0,0,0,0,0,0,0,0,0,0,0,0,0,0,0,0,0,0,0,0,0,0,0,0,0,0,0,0,0,0,0

	
```

```
		  

		FSP34_Contig02, Position,0,100,200,300,400,500,600,700,800,900,1000,1100,1200,1300,1400,1500,1600,1700,1800,1900,2000,2100,2200,2300,2400,2500,2600,2700,2800,2900,3000,3100,3200,3300,3400,3500,3600,3700,3800,3900,4000,4100,4200,4300,4400,4500,4600,4700,4800,4900,5000,5100,5200,5300,5400,5500,5600,5700,5800,5900,6000,6100,6200,6300,6400,6500,6600,6700,6800,6900,7000,7100,7200,7300,7400,7500,7600,7700,7800,7900,8000,8100,8200,8300,8400,8500,8600,8700,8800,8900,9000,9100,9200,9300,9400,9500,9600,9700,9800,9900,10000,10100,10200,10300,10400,10500,10600,10700,10800,10900,11000,11100,11200,11300,11400,11500,11600,11700,11800,11900,12000,12100,12200,12300,12400,12500,12600,12700,12800,12900,13000,13100,13200,13300,13400,13500,13600,13700,13800,13900,14000,14100,14200,14300,14400,14500,14600,14700,14800,14900,15000,15100,15200,15300,15400,15500,15600,15700,15800,15900,16000,16100,16200,16300,16400,16500,16600,16700,16800,16900,17000,17100,17200,17300,17400,17500,17600,17700,17800,17900,18000,18100,18200,18300,18400,18500,18600,18700,18800,18900,19000,19100,19200,19300,19400,19500,19600,19700,19800,19900,20000,20100,20200,20300,20400,20500,20600,20700,20800,20900,21000,21100,21200,21300,21400,21500,21600,21700,21800,21900,22000,22100,22200,22300,22400,22500,22600,22700,22800,22900,23000,23100,23200,23300,23400,23500,23600,23700,23800,23900,24000,24100,24200,24300,24400,24500,24600,24700,24800,24900,25000,25100,25200,25300,25400,25500,25600,25700,25800,25900,26000,26100,26200,26300,26400,26500,26600,26700,26800,26900,27000,27100,27200,27300,27400,27500,27600,27700
FSP34_Contig02,Count,0,0,0,0,0,0,0,0,0,0,0,0,0,0,0,0,0,0,0,0,0,0,0,0,0,0,0,0,0,0,0,0,0,0,0,0,0,0,0,0,0,0,0,0,0,0,0,0,0,0,0,0,0,0,0,0,0,0,0,0,0,0,0,0,0,0,0,0,0,0,0,0,0,0,0,0,0,0,0,0,0,0,0,0,0,0,0,0,0,0,0,0,0,0,0,0,0,0,0,0,0,0,0,0,0,0,0,0,0,0,0,0,0,0,0,0,0,0,1,0,0,0,0,0,0,0,0,0,0,0,0,0,0,0,0,0,0,0,0,0,1,0,0,0,0,0,0,0,0,0,0,0,0,0,0,0,0,0,0,1,0,0,0,0,0,0,0,0,0,0,0,0,0,0,0,0,0,0,0,0,0,0,0,0,0,0,0,0,0,0,0,0,0,0,0,0,0,0,0,0,0,0,0,0,0,0,0,0,0,0,0,0,0,0,0,0,0,0,0,0,0,0,0,0,0,0,0,0,0,0,0,0,0,0,0,0,0,0,0,0,0,0,0,0,0,0,0,0,0,0,0,0,0,0,0,0,0,0,0,0,0,0,0,0,0,0,0,0,0,0,0,0,0,0,0,0,0,0

	
```

```
		  

		FSP34_Mitochondrion, Position,0,1000,2000,3000,4000,5000,6000,7000,8000,9000,10000,11000,12000,13000,14000,15000,16000,17000,18000,19000,20000,21000,22000,23000,24000,25000,26000,27000,28000,29000,30000,31000,32000,33000,34000,35000,36000,37000,38000,39000,40000,41000,42000,43000,44000,45000,46000,47000,48000,49000,50000,51000,52000,53000,54000,55000,56000,57000,58000,59000,60000,61000,62000,63000,64000,65000,66000,67000,68000,69000,70000,71000,72000,73000,74000,75000,76000,77000,78000,79000,80000,81000
FSP34_Mitochondrion,Count,0,0,0,0,0,0,0,0,0,0,1,1,2,1,0,0,0,0,0,0,0,0,0,0,0,0,0,0,0,0,0,0,0,0,0,0,0,0,0,0,0,0,0,0,0,0,0,0,0,0,0,0,0,0,0,0,0,0,0,0,0,0,1,0,0,0,0,0,0,0,0,0,0,0,0,0,0,0,0,0,0,0

	
```


---

 **Details by gene** 

**Here** you can find a tab-separated table.
