# Supplementary material for: Intra-Species Genomic Variation in the Pine Pathogen Fusarium circinatum
Source: J Fungi (Basel). 2022 Jun 23;8(7):657. doi: 10.3390/jof8070657 (PMC9316270; doi:10.3390/jof8070657)
Supplement: Supplementary file 1 [file jof-08-00657-s001.zip › Supplementary S5.html]

### SnpEff: Variant analysis

|  |
| --- |
| **Contents** Summary   Variant rate by chromosome  Variants by type   Number of variants by impact    Number of variants by functional class    Number of variants by effect   Quality histogram  InDel length histogram  Base variant table  Transition vs transversions (ts/tv)   Allele frequency    Allele Count    Codon change table    Amino acid change table    Chromosome variants plots    Details by gene |


---


**Summary**

|  |  |
| --- | --- |
| **Genome** | fsp34 |
| **Date** | 2018-12-13 17:17 |
| **SnpEff version** | ``` SnpEff 4.3t (build 2017-11-24 10:18), by Pablo Cingolani ``` |
| **Command line arguments** | ``` SnpEff  fsp34 populationfreebayesm30q20filteredq30dp10.vcf ``` |
| **Warnings** | 1,044 |
| **Errors** | 0 |
| **Number of lines (input file)** | 397,704 |
| **Number of variants (before filter)** | 461,683 |
| **Number of not variants  (i.e. reference equals alternative)** | 0 |
| **Number of variants processed   (i.e. after filter and non-variants)** | 461,683 |
| **Number of known variants  (i.e. non-empty ID)** | 0 ( 0% ) |
| **Number of multi-allelic VCF entries  (i.e. more than two alleles)** | 44,587 |
| **Number of effects** | 2,719,213 |
| **Genome total length** | 44,945,038 |
| **Genome effective length** | 44,943,885 |
| **Variant rate** | 1 variant every 97 bases |


---


 **Variants rate details** 

| Chromosome | Length | Variants | Variants rate |
| --- | --- | --- | --- |
| FSP34\_Chr01 | 6,407,589 | 41,809 | 153 |
| FSP34\_Chr02 | 5,065,897 | 44,429 | 114 |
| FSP34\_Chr03 | 5,081,688 | 35,947 | 141 |
| FSP34\_Chr04 | 4,313,068 | 42,594 | 101 |
| FSP34\_Chr05 | 4,432,453 | 36,398 | 121 |
| FSP34\_Chr06 | 4,301,695 | 44,223 | 97 |
| FSP34\_Chr07 | 3,540,654 | 36,258 | 97 |
| FSP34\_Chr08 | 3,172,515 | 43,130 | 73 |
| FSP34\_Chr09 | 2,981,544 | 31,971 | 93 |
| FSP34\_Chr10 | 2,698,620 | 43,870 | 61 |
| FSP34\_Chr11 | 2,228,220 | 36,755 | 60 |
| FSP34\_Chr12 | 525,065 | 17,314 | 30 |
| FSP34\_Contig01 | 85,668 | 5,092 | 16 |
| FSP34\_Contig02 | 27,708 | 1,848 | 14 |
| FSP34\_Mitochondrion | 81,501 | 45 | 1,811 |
| Total | 44,943,885 | 461,683 | 97 |


---


 **Number variants by type**

| **Type** | **Total** |
| --- | --- |
| **SNP** | 316,965 |
| **MNP** | 106,426 |
| **INS** | 14,575 |
| **DEL** | 11,140 |
| **MIXED** | 12,577 |
| **INV** | 0 |
| **DUP** | 0 |
| **BND** | 0 |
| **INTERVAL** | 0 |
| **Total** | 461,683 |
| --- | --- |


---


 **Number of effects by impact** 

| **Type (alphabetical order)** |  | Count | Percent |
| --- | --- | --- | --- |
| **HIGH** |  | 18,047 | 0.664% |
| **LOW** |  | 74,845 | 2.752% |
| **MODERATE** |  | 63,033 | 2.318% |
| **MODIFIER** |  | 2,563,288 | 94.266% |


---


 **Number of effects by functional class** 

| **Type (alphabetical order)** |  | Count | Percent |
| --- | --- | --- | --- |
| **MISSENSE** |  | 46,372 | 42.007% |
| **NONSENSE** |  | 1,027 | 0.93% |
| **SILENT** |  | 62,991 | 57.062% |

  

Missense / Silent ratio: 0.7362


---


 **Number of effects by type and region** 

| Type | Region |
| --- | --- |
| | **Type (alphabetical order)** |  | Count | Percent | | --- | --- | --- | --- | | **conservative\_inframe\_deletion** |  | 404 | 0.015% | | **conservative\_inframe\_insertion** |  | 396 | 0.015% | | **disruptive\_inframe\_deletion** |  | 354 | 0.013% | | **disruptive\_inframe\_insertion** |  | 216 | 0.008% | | **downstream\_gene\_variant** |  | 977,631 | 35.858% | | **frameshift\_variant** |  | 1,881 | 0.069% | | **gene\_fusion** |  | 13,881 | 0.509% | | **initiator\_codon\_variant** |  | 17 | 0.001% | | **intergenic\_region** |  | 277,047 | 10.162% | | **intragenic\_variant** |  | 168,282 | 6.172% | | **intron\_variant** |  | 23,572 | 0.865% | | **missense\_variant** |  | 62,233 | 2.283% | | **non\_coding\_transcript\_variant** |  | 133,146 | 4.884% | | **splice\_acceptor\_variant** |  | 287 | 0.011% | | **splice\_donor\_variant** |  | 325 | 0.012% | | **splice\_region\_variant** |  | 5,689 | 0.209% | | **start\_lost** |  | 110 | 0.004% | | **stop\_gained** |  | 1,434 | 0.053% | | **stop\_lost** |  | 269 | 0.01% | | **stop\_retained\_variant** |  | 170 | 0.006% | | **synonymous\_variant** |  | 71,054 | 2.606% | | **upstream\_gene\_variant** |  | 988,027 | 36.239% | | | **Type (alphabetical order)** |  | Count | Percent | | --- | --- | --- | --- | | **DOWNSTREAM** |  | 977,631 | 35.953% | | **EXON** |  | 136,959 | 5.037% | | **GENE** |  | 13,881 | 0.51% | | **INTERGENIC** |  | 277,047 | 10.188% | | **INTRON** |  | 19,155 | 0.704% | | **SPLICE\_SITE\_ACCEPTOR** |  | 266 | 0.01% | | **SPLICE\_SITE\_DONOR** |  | 301 | 0.011% | | **SPLICE\_SITE\_REGION** |  | 4,518 | 0.166% | | **TRANSCRIPT** |  | 301,428 | 11.085% | | **UPSTREAM** |  | 988,027 | 36.335% | |


---


 **Quality:**

```
|  |  |
| --- | --- |
| Min | 30 |
| Max | 124,509 |
| Mean | 3,151.808 |
| Median | 2,272 |
| Standard deviation | 2,733.431 |
| Values | 30,31,32,33,34,35,36,37,38,39,40,41,42,43,44,45,46,47,48,49,50,51,52,53,54,55,56,57,58,59,60,61,62,63,64,65,66,67,68,69,70,71,72,73,74,75,76,77,78,79,80,81,82,83,84,85,86,87,88,89,90,91,92,93,94,95,96,97,98,99,100,101,102,103,104,105,106,107,108,109,110,111,112,113,114,115,116,117,118,119,120,121,122,123,124,125,126,127,128,129,130,131,132,133,134,135,136,137,138,139,140,141,142,143,144,145,146,147,148,149,150,151,152,153,154,155,156,157,158,159,160,161,162,163,164,165,166,167,168,169,170,171,172,173,174,175,176,177,178,179,180,181,182,183,184,185,186,187,188,189,190,191,192,193,194,195,196,197,198,199,200,201,202,203,204,205,206,207,208,209,210,211,212,213,214,215,216,217,218,219,220,221,222,223,224,225,226,227,228,229,230,231,232,233,234,235,236,237,238,239,240,241,242,243,244,245,246,247,248,249,250,251,252,253,254,255,256,257,258,259,260,261,262,263,264,265,266,267,268,269,270,271,272,273,274,275,276,277,278,279,280,281,282,283,284,285,286,287,288,289,290,291,292,293,294,295,296,297,298,299,300,301,302,303,304,305,306,307,308,309,310,311,312,313,314,315,316,317,318,319,320,321,322,323,324,325,326,327,328,329,330,331,332,333,334,335,336,337,338,339,340,341,342,343,344,345,346,347,348,349,350,351,352,353,354,355,356,357,358,359,360,361,362,363,364,365,366,367,368,369,370,371,372,373,374,375,376,377,378,379,380,381,382,383,384,385,386,387,388,389,390,391,392,393,394,395,396,397,398,399,400,401,402,403,404,405,406,407,408,409,410,411,412,413,414,415,416,417,418,419,420,421,422,423,424,425,426,427,428,429,430,431,432,433,434,435,436,437,438,439,440,441,442,443,444,445,446,447,448,449,450,451,452,453,454,455,456,457,458,459,460,461,462,463,464,465,466,467,468,469,470,471,472,473,474,475,476,477,478,479,480,481,482,483,484,485,486,487,488,489,490,491,492,493,494,495,496,497,498,499,500,501,502,503,504,505,506,507,508,509,510,511,512,513,514,515,516,517,518,519,520,521,522,523,524,525,526,527,528,529,530,531,532,533,534,535,536,537,538,539,540,541,542,543,544,545,546,547,548,549,550,551,552,553,554,555,556,557,558,559,560,561,562,563,564,565,566,567,568,569,570,571,572,573,574,575,576,577,578,579,580,581,582,583,584,585,586,587,588,589,590,591,592,593,594,595,596,597,598,599,600,601,602,603,604,605,606,607,608,609,610,611,612,613,614,615,616,617,618,619,620,621,622,623,624,625,626,627,628,629,630,631,632,633,634,635,636,637,638,639,640,641,642,643,644,645,646,647,648,649,650,651,652,653,654,655,656,657,658,659,660,661,662,663,664,665,666,667,668,669,670,671,672,673,674,675,676,677,678,679,680,681,682,683,684,685,686,687,688,689,690,691,692,693,694,695,696,697,698,699,700,701,702,703,704,705,706,707,708,709,710,711,712,713,714,715,716,717,718,719,720,721,722,723,724,725,726,727,728,729,730,731,732,733,734,735,736,737,738,739,740,741,742,743,744,745,746,747,748,749,750,751,752,753,754,755,756,757,758,759,760,761,762,763,764,765,766,767,768,769,770,771,772,773,774,775,776,777,778,779,780,781,782,783,784,785,786,787,788,789,790,791,792,793,794,795,796,797,798,799,800,801,802,803,804,805,806,807,808,809,810,811,812,813,814,815,816,817,818,819,820,821,822,823,824,825,826,827,828,829,830,831,832,833,834,835,836,837,838,839,840,841,842,843,844,845,846,847,848,849,850,851,852,853,854,855,856,857,858,859,860,861,862,863,864,865,866,867,868,869,870,871,872,873,874,875,876,877,878,879,880,881,882,883,884,885,886,887,888,889,890,891,892,893,894,895,896,897,898,899,900,901,902,903,904,905,906,907,908,909,910,911,912,913,914,915,916,917,918,919,920,921,922,923,924,925,926,927,928,929,930,931,932,933,934,935,936,937,938,939,940,941,942,943,944,945,946,947,948,949,950,951,952,953,954,955,956,957,958,959,960,961,962,963,964,965,966,967,968,969,970,971,972,973,974,975,976,977,978,979,980,981,982,983,984,985,986,987,988,989,990,991,992,993,994,995,996,997,998,999,1000,1001,1002,1003,1004,1005,1006,1007,1008,1009,1010,1011,1012,1013,1014,1015,1016,1017,1018,1019,1020,1021,1022,1023,1024,1025,1026,1027,1028,1029,1030,1031,1032,1033,1034,1035,1036,1037,1038,1039,1040,1041,1042,1043,1044,1045,1046,1047,1048,1049,1050,1051,1052,1053,1054,1055,1056,1057,1058,1059,1060,1061,1062,1063,1064,1065,1066,1067,1068,1069,1070,1071,1072,1073,1074,1075,1076,1077,1078,1079,1080,1081,1082,1083,1084,1085,1086,1087,1088,1089,1090,1091,1092,1093,1094,1095,1096,1097,1098,1099,1100,1101,1102,1103,1104,1105,1106,1107,1108,1109,1110,1111,1112,1113,1114,1115,1116,1117,1118,1119,1120,1121,1122,1123,1124,1125,1126,1127,1128,1129,1130,1131,1132,1133,1134,1135,1136,1137,1138,1139,1140,1141,1142,1143,1144,1145,1146,1147,1148,1149,1150,1151,1152,1153,1154,1155,1156,1157,1158,1159,1160,1161,1162,1163,1164,1165,1166,1167,1168,1169,1170,1171,1172,1173,1174,1175,1176,1177,1178,1179,1180,1181,1182,1183,1184,1185,1186,1187,1188,1189,1190,1191,1192,1193,1194,1195,1196,1197,1198,1199,1200,1201,1202,1203,1204,1205,1206,1207,1208,1209,1210,1211,1212,1213,1214,1215,1216,1217,1218,1219,1220,1221,1222,1223,1224,1225,1226,1227,1228,1229,1230,1231,1232,1233,1234,1235,1236,1237,1238,1239,1240,1241,1242,1243,1244,1245,1246,1247,1248,1249,1250,1251,1252,1253,1254,1255,1256,1257,1258,1259,1260,1261,1262,1263,1264,1265,1266,1267,1268,1269,1270,1271,1272,1273,1274,1275,1276,1277,1278,1279,1280,1281,1282,1283,1284,1285,1286,1287,1288,1289,1290,1291,1292,1293,1294,1295,1296,1297,1298,1299,1300,1301,1302,1303,1304,1305,1306,1307,1308,1309,1310,1311,1312,1313,1314,1315,1316,1317,1318,1319,1320,1321,1322,1323,1324,1325,1326,1327,1328,1329,1330,1331,1332,1333,1334,1335,1336,1337,1338,1339,1340,1341,1342,1343,1344,1345,1346,1347,1348,1349,1350,1351,1352,1353,1354,1355,1356,1357,1358,1359,1360,1361,1362,1363,1364,1365,1366,1367,1368,1369,1370,1371,1372,1373,1374,1375,1376,1377,1378,1379,1380,1381,1382,1383,1384,1385,1386,1387,1388,1389,1390,1391,1392,1393,1394,1395,1396,1397,1398,1399,1400,1401,1402,1403,1404,1405,1406,1407,1408,1409,1410,1411,1412,1413,1414,1415,1416,1417,1418,1419,1420,1421,1422,1423,1424,1425,1426,1427,1428,1429,1430,1431,1432,1433,1434,1435,1436,1437,1438,1439,1440,1441,1442,1443,1444,1445,1446,1447,1448,1449,1450,1451,1452,1453,1454,1455,1456,1457,1458,1459,1460,1461,1462,1463,1464,1465,1466,1467,1468,1469,1470,1471,1472,1473,1474,1475,1476,1477,1478,1479,1480,1481,1482,1483,1484,1485,1486,1487,1488,1489,1490,1491,1492,1493,1494,1495,1496,1497,1498,1499,1500,1501,1502,1503,1504,1505,1506,1507,1508,1509,1510,1511,1512,1513,1514,1515,1516,1517,1518,1519,1520,1521,1522,1523,1524,1525,1526,1527,1528,1529,1530,1531,1532,1533,1534,1535,1536,1537,1538,1539,1540,1541,1542,1543,1544,1545,1546,1547,1548,1549,1550,1551,1552,1553,1554,1555,1556,1557,1558,1559,1560,1561,1562,1563,1564,1565,1566,1567,1568,1569,1570,1571,1572,1573,1574,1575,1576,1577,1578,1579,1580,1581,1582,1583,1584,1585,1586,1587,1588,1589,1590,1591,1592,1593,1594,1595,1596,1597,1598,1599,1600,1601,1602,1603,1604,1605,1606,1607,1608,1609,1610,1611,1612,1613,1614,1615,1616,1617,1618,1619,1620,1621,1622,1623,1624,1625,1626,1627,1628,1629,1630,1631,1632,1633,1634,1635,1636,1637,1638,1639,1640,1641,1642,1643,1644,1645,1646,1647,1648,1649,1650,1651,1652,1653,1654,1655,1656,1657,1658,1659,1660,1661,1662,1663,1664,1665,1666,1667,1668,1669,1670,1671,1672,1673,1674,1675,1676,1677,1678,1679,1680,1681,1682,1683,1684,1685,1686,1687,1688,1689,1690,1691,1692,1693,1694,1695,1696,1697,1698,1699,1700,1701,1702,1703,1704,1705,1706,1707,1708,1709,1710,1711,1712,1713,1714,1715,1716,1717,1718,1719,1720,1721,1722,1723,1724,1725,1726,1727,1728,1729,1730,1731,1732,1733,1734,1735,1736,1737,1738,1739,1740,1741,1742,1743,1744,1745,1746,1747,1748,1749,1750,1751,1752,1753,1754,1755,1756,1757,1758,1759,1760,1761,1762,1763,1764,1765,1766,1767,1768,1769,1770,1771,1772,1773,1774,1775,1776,1777,1778,1779,1780,1781,1782,1783,1784,1785,1786,1787,1788,1789,1790,1791,1792,1793,1794,1795,1796,1797,1798,1799,1800,1801,1802,1803,1804,1805,1806,1807,1808,1809,1810,1811,1812,1813,1814,1815,1816,1817,1818,1819,1820,1821,1822,1823,1824,1825,1826,1827,1828,1829,1830,1831,1832,1833,1834,1835,1836,1837,1838,1839,1840,1841,1842,1843,1844,1845,1846,1847,1848,1849,1850,1851,1852,1853,1854,1855,1856,1857,1858,1859,1860,1861,1862,1863,1864,1865,1866,1867,1868,1869,1870,1871,1872,1873,1874,1875,1876,1877,1878,1879,1880,1881,1882,1883,1884,1885,1886,1887,1888,1889,1890,1891,1892,1893,1894,1895,1896,1897,1898,1899,1900,1901,1902,1903,1904,1905,1906,1907,1908,1909,1910,1911,1912,1913,1914,1915,1916,1917,1918,1919,1920,1921,1922,1923,1924,1925,1926,1927,1928,1929,1930,1931,1932,1933,1934,1935,1936,1937,1938,1939,1940,1941,1942,1943,1944,1945,1946,1947,1948,1949,1950,1951,1952,1953,1954,1955,1956,1957,1958,1959,1960,1961,1962,1963,1964,1965,1966,1967,1968,1969,1970,1971,1972,1973,1974,1975,1976,1977,1978,1979,1980,1981,1982,1983,1984,1985,1986,1987,1988,1989,1990,1991,1992,1993,1994,1995,1996,1997,1998,1999,2000,2001,2002,2003,2004,2005,2006,2007,2008,2009,2010,2011,2012,2013,2014,2015,2016,2017,2018,2019,2020,2021,2022,2023,2024,2025,2026,2027,2028,2029,2030,2031,2032,2033,2034,2035,2036,2037,2038,2039,2040,2041,2042,2043,2044,2045,2046,2047,2048,2049,2050,2051,2052,2053,2054,2055,2056,2057,2058,2059,2060,2061,2062,2063,2064,2065,2066,2067,2068,2069,2070,2071,2072,2073,2074,2075,2076,2077,2078,2079,2080,2081,2082,2083,2084,2085,2086,2087,2088,2089,2090,2091,2092,2093,2094,2095,2096,2097,2098,2099,2100,2101,2102,2103,2104,2105,2106,2107,2108,2109,2110,2111,2112,2113,2114,2115,2116,2117,2118,2119,2120,2121,2122,2123,2124,2125,2126,2127,2128,2129,2130,2131,2132,2133,2134,2135,2136,2137,2138,2139,2140,2141,2142,2143,2144,2145,2146,2147,2148,2149,2150,2151,2152,2153,2154,2155,2156,2157,2158,2159,2160,2161,2162,2163,2164,2165,2166,2167,2168,2169,2170,2171,2172,2173,2174,2175,2176,2177,2178,2179,2180,2181,2182,2183,2184,2185,2186,2187,2188,2189,2190,2191,2192,2193,2194,2195,2196,2197,2198,2199,2200,2201,2202,2203,2204,2205,2206,2207,2208,2209,2210,2211,2212,2213,2214,2215,2216,2217,2218,2219,2220,2221,2222,2223,2224,2225,2226,2227,2228,2229,2230,2231,2232,2233,2234,2235,2236,2237,2238,2239,2240,2241,2242,2243,2244,2245,2246,2247,2248,2249,2250,2251,2252,2253,2254,2255,2256,2257,2258,2259,2260,2261,2262,2263,2264,2265,2266,2267,2268,2269,2270,2271,2272,2273,2274,2275,2276,2277,2278,2279,2280,2281,2282,2283,2284,2285,2286,2287,2288,2289,2290,2291,2292,2293,2294,2295,2296,2297,2298,2299,2300,2301,2302,2303,2304,2305,2306,2307,2308,2309,2310,2311,2312,2313,2314,2315,2316,2317,2318,2319,2320,2321,2322,2323,2324,2325,2326,2327,2328,2329,2330,2331,2332,2333,2334,2335,2336,2337,2338,2339,2340,2341,2342,2343,2344,2345,2346,2347,2348,2349,2350,2351,2352,2353,2354,2355,2356,2357,2358,2359,2360,2361,2362,2363,2364,2365,2366,2367,2368,2369,2370,2371,2372,2373,2374,2375,2376,2377,2378,2379,2380,2381,2382,2383,2384,2385,2386,2387,2388,2389,2390,2391,2392,2393,2394,2395,2396,2397,2398,2399,2400,2401,2402,2403,2404,2405,2406,2407,2408,2409,2410,2411,2412,2413,2414,2415,2416,2417,2418,2419,2420,2421,2422,2423,2424,2425,2426,2427,2428,2429,2430,2431,2432,2433,2434,2435,2436,2437,2438,2439,2440,2441,2442,2443,2444,2445,2446,2447,2448,2449,2450,2451,2452,2453,2454,2455,2456,2457,2458,2459,2460,2461,2462,2463,2464,2465,2466,2467,2468,2469,2470,2471,2472,2473,2474,2475,2476,2477,2478,2479,2480,2481,2482,2483,2484,2485,2486,2487,2488,2489,2490,2491,2492,2493,2494,2495,2496,2497,2498,2499,2500,2501,2502,2503,2504,2505,2506,2507,2508,2509,2510,2511,2512,2513,2514,2515,2516,2517,2518,2519,2520,2521,2522,2523,2524,2525,2526,2527,2528,2529,2530,2531,2532,2533,2534,2535,2536,2537,2538,2539,2540,2541,2542,2543,2544,2545,2546,2547,2548,2549,2550,2551,2552,2553,2554,2555,2556,2557,2558,2559,2560,2561,2562,2563,2564,2565,2566,2567,2568,2569,2570,2571,2572,2573,2574,2575,2576,2577,2578,2579,2580,2581,2582,2583,2584,2585,2586,2587,2588,2589,2590,2591,2592,2593,2594,2595,2596,2597,2598,2599,2600,2601,2602,2603,2604,2605,2606,2607,2608,2609,2610,2611,2612,2613,2614,2615,2616,2617,2618,2619,2620,2621,2622,2623,2624,2625,2626,2627,2628,2629,2630,2631,2632,2633,2634,2635,2636,2637,2638,2639,2640,2641,2642,2643,2644,2645,2646,2647,2648,2649,2650,2651,2652,2653,2654,2655,2656,2657,2658,2659,2660,2661,2662,2663,2664,2665,2666,2667,2668,2669,2670,2671,2672,2673,2674,2675,2676,2677,2678,2679,2680,2681,2682,2683,2684,2685,2686,2687,2688,2689,2690,2691,2692,2693,2694,2695,2696,2697,2698,2699,2700,2701,2702,2703,2704,2705,2706,2707,2708,2709,2710,2711,2712,2713,2714,2715,2716,2717,2718,2719,2720,2721,2722,2723,2724,2725,2726,2727,2728,2729,2730,2731,2732,2733,2734,2735,2736,2737,2738,2739,2740,2741,2742,2743,2744,2745,2746,2747,2748,2749,2750,2751,2752,2753,2754,2755,2756,2757,2758,2759,2760,2761,2762,2763,2764,2765,2766,2767,2768,2769,2770,2771,2772,2773,2774,2775,2776,2777,2778,2779,2780,2781,2782,2783,2784,2785,2786,2787,2788,2789,2790,2791,2792,2793,2794,2795,2796,2797,2798,2799,2800,2801,2802,2803,2804,2805,2806,2807,2808,2809,2810,2811,2812,2813,2814,2815,2816,2817,2818,2819,2820,2821,2822,2823,2824,2825,2826,2827,2828,2829,2830,2831,2832,2833,2834,2835,2836,2837,2838,2839,2840,2841,2842,2843,2844,2845,2846,2847,2848,2849,2850,2851,2852,2853,2854,2855,2856,2857,2858,2859,2860,2861,2862,2863,2864,2865,2866,2867,2868,2869,2870,2871,2872,2873,2874,2875,2876,2877,2878,2879,2880,2881,2882,2883,2884,2885,2886,2887,2888,2889,2890,2891,2892,2893,2894,2895,2896,2897,2898,2899,2900,2901,2902,2903,2904,2905,2906,2907,2908,2909,2910,2911,2912,2913,2914,2915,2916,2917,2918,2919,2920,2921,2922,2923,2924,2925,2926,2927,2928,2929,2930,2931,2932,2933,2934,2935,2936,2937,2938,2939,2940,2941,2942,2943,2944,2945,2946,2947,2948,2949,2950,2951,2952,2953,2954,2955,2956,2957,2958,2959,2960,2961,2962,2963,2964,2965,2966,2967,2968,2969,2970,2971,2972,2973,2974,2975,2976,2977,2978,2979,2980,2981,2982,2983,2984,2985,2986,2987,2988,2989,2990,2991,2992,2993,2994,2995,2996,2997,2998,2999,3000,3001,3002,3003,3004,3005,3006,3007,3008,3009,3010,3011,3012,3013,3014,3015,3016,3017,3018,3019,3020,3021,3022,3023,3024,3025,3026,3027,3028,3029,3030,3031,3032,3033,3034,3035,3036,3037,3038,3039,3040,3041,3042,3043,3044,3045,3046,3047,3048,3049,3050,3051,3052,3053,3054,3055,3056,3057,3058,3059,3060,3061,3062,3063,3064,3065,3066,3067,3068,3069,3070,3071,3072,3073,3074,3075,3076,3077,3078,3079,3080,3081,3082,3083,3084,3085,3086,3087,3088,3089,3090,3091,3092,3093,3094,3095,3096,3097,3098,3099,3100,3101,3102,3103,3104,3105,3106,3107,3108,3109,3110,3111,3112,3113,3114,3115,3116,3117,3118,3119,3120,3121,3122,3123,3124,3125,3126,3127,3128,3129,3130,3131,3132,3133,3134,3135,3136,3137,3138,3139,3140,3141,3142,3143,3144,3145,3146,3147,3148,3149,3150,3151,3152,3153,3154,3155,3156,3157,3158,3159,3160,3161,3162,3163,3164,3165,3166,3167,3168,3169,3170,3171,3172,3173,3174,3175,3176,3177,3178,3179,3180,3181,3182,3183,3184,3185,3186,3187,3188,3189,3190,3191,3192,3193,3194,3195,3196,3197,3198,3199,3200,3201,3202,3203,3204,3205,3206,3207,3208,3209,3210,3211,3212,3213,3214,3215,3216,3217,3218,3219,3220,3221,3222,3223,3224,3225,3226,3227,3228,3229,3230,3231,3232,3233,3234,3235,3236,3237,3238,3239,3240,3241,3242,3243,3244,3245,3246,3247,3248,3249,3250,3251,3252,3253,3254,3255,3256,3257,3258,3259,3260,3261,3262,3263,3264,3265,3266,3267,3268,3269,3270,3271,3272,3273,3274,3275,3276,3277,3278,3279,3280,3281,3282,3283,3284,3285,3286,3287,3288,3289,3290,3291,3292,3293,3294,3295,3296,3297,3298,3299,3300,3301,3302,3303,3304,3305,3306,3307,3308,3309,3310,3311,3312,3313,3314,3315,3316,3317,3318,3319,3320,3321,3322,3323,3324,3325,3326,3327,3328,3329,3330,3331,3332,3333,3334,3335,3336,3337,3338,3339,3340,3341,3342,3343,3344,3345,3346,3347,3348,3349,3350,3351,3352,3353,3354,3355,3356,3357,3358,3359,3360,3361,3362,3363,3364,3365,3366,3367,3368,3369,3370,3371,3372,3373,3374,3375,3376,3377,3378,3379,3380,3381,3382,3383,3384,3385,3386,3387,3388,3389,3390,3391,3392,3393,3394,3395,3396,3397,3398,3399,3400,3401,3402,3403,3404,3405,3406,3407,3408,3409,3410,3411,3412,3413,3414,3415,3416,3417,3418,3419,3420,3421,3422,3423,3424,3425,3426,3427,3428,3429,3430,3431,3432,3433,3434,3435,3436,3437,3438,3439,3440,3441,3442,3443,3444,3445,3446,3447,3448,3449,3450,3451,3452,3453,3454,3455,3456,3457,3458,3459,3460,3461,3462,3463,3464,3465,3466,3467,3468,3469,3470,3471,3472,3473,3474,3475,3476,3477,3478,3479,3480,3481,3482,3483,3484,3485,3486,3487,3488,3489,3490,3491,3492,3493,3494,3495,3496,3497,3498,3499,3500,3501,3502,3503,3504,3505,3506,3507,3508,3509,3510,3511,3512,3513,3514,3515,3516,3517,3518,3519,3520,3521,3522,3523,3524,3525,3526,3527,3528,3529,3530,3531,3532,3533,3534,3535,3536,3537,3538,3539,3540,3541,3542,3543,3544,3545,3546,3547,3548,3549,3550,3551,3552,3553,3554,3555,3556,3557,3558,3559,3560,3561,3562,3563,3564,3565,3566,3567,3568,3569,3570,3571,3572,3573,3574,3575,3576,3577,3578,3579,3580,3581,3582,3583,3584,3585,3586,3587,3588,3589,3590,3591,3592,3593,3594,3595,3596,3597,3598,3599,3600,3601,3602,3603,3604,3605,3606,3607,3608,3609,3610,3611,3612,3613,3614,3615,3616,3617,3618,3619,3620,3621,3622,3623,3624,3625,3626,3627,3628,3629,3630,3631,3632,3633,3634,3635,3636,3637,3638,3639,3640,3641,3642,3643,3644,3645,3646,3647,3648,3649,3650,3651,3652,3653,3654,3655,3656,3657,3658,3659,3660,3661,3662,3663,3664,3665,3666,3667,3668,3669,3670,3671,3672,3673,3674,3675,3676,3677,3678,3679,3680,3681,3682,3683,3684,3685,3686,3687,3688,3689,3690,3691,3692,3693,3694,3695,3696,3697,3698,3699,3700,3701,3702,3703,3704,3705,3706,3707,3708,3709,3710,3711,3712,3713,3714,3715,3716,3717,3718,3719,3720,3721,3722,3723,3724,3725,3726,3727,3728,3729,3730,3731,3732,3733,3734,3735,3736,3737,3738,3739,3740,3741,3742,3743,3744,3745,3746,3747,3748,3749,3750,3751,3752,3753,3754,3755,3756,3757,3758,3759,3760,3761,3762,3763,3764,3765,3766,3767,3768,3769,3770,3771,3772,3773,3774,3775,3776,3777,3778,3779,3780,3781,3782,3783,3784,3785,3786,3787,3788,3789,3790,3791,3792,3793,3794,3795,3796,3797,3798,3799,3800,3801,3802,3803,3804,3805,3806,3807,3808,3809,3810,3811,3812,3813,3814,3815,3816,3817,3818,3819,3820,3821,3822,3823,3824,3825,3826,3827,3828,3829,3830,3831,3832,3833,3834,3835,3836,3837,3838,3839,3840,3841,3842,3843,3844,3845,3846,3847,3848,3849,3850,3851,3852,3853,3854,3855,3856,3857,3858,3859,3860,3861,3862,3863,3864,3865,3866,3867,3868,3869,3870,3871,3872,3873,3874,3875,3876,3877,3878,3879,3880,3881,3882,3883,3884,3885,3886,3887,3888,3889,3890,3891,3892,3893,3894,3895,3896,3897,3898,3899,3900,3901,3902,3903,3904,3905,3906,3907,3908,3909,3910,3911,3912,3913,3914,3915,3916,3917,3918,3919,3920,3921,3922,3923,3924,3925,3926,3927,3928,3929,3930,3931,3932,3933,3934,3935,3936,3937,3938,3939,3940,3941,3942,3943,3944,3945,3946,3947,3948,3949,3950,3951,3952,3953,3954,3955,3956,3957,3958,3959,3960,3961,3962,3963,3964,3965,3966,3967,3968,3969,3970,3971,3972,3973,3974,3975,3976,3977,3978,3979,3980,3981,3982,3983,3984,3985,3986,3987,3988,3989,3990,3991,3992,3993,3994,3995,3996,3997,3998,3999,4000,4001,4002,4003,4004,4005,4006,4007,4008,4009,4010,4011,4012,4013,4014,4015,4016,4017,4018,4019,4020,4021,4022,4023,4024,4025,4026,4027,4028,4029,4030,4031,4032,4033,4034,4035,4036,4037,4038,4039,4040,4041,4042,4043,4044,4045,4046,4047,4048,4049,4050,4051,4052,4053,4054,4055,4056,4057,4058,4059,4060,4061,4062,4063,4064,4065,4066,4067,4068,4069,4070,4071,4072,4073,4074,4075,4076,4077,4078,4079,4080,4081,4082,4083,4084,4085,4086,4087,4088,4089,4090,4091,4092,4093,4094,4095,4096,4097,4098,4099,4100,4101,4102,4103,4104,4105,4106,4107,4108,4109,4110,4111,4112,4113,4114,4115,4116,4117,4118,4119,4120,4121,4122,4123,4124,4125,4126,4127,4128,4129,4130,4131,4132,4133,4134,4135,4136,4137,4138,4139,4140,4141,4142,4143,4144,4145,4146,4147,4148,4149,4150,4151,4152,4153,4154,4155,4156,4157,4158,4159,4160,4161,4162,4163,4164,4165,4166,4167,4168,4169,4170,4171,4172,4173,4174,4175,4176,4177,4178,4179,4180,4181,4182,4183,4184,4185,4186,4187,4188,4189,4190,4191,4192,4193,4194,4195,4196,4197,4198,4199,4200,4201,4202,4203,4204,4205,4206,4207,4208,4209,4210,4211,4212,4213,4214,4215,4216,4217,4218,4219,4220,4221,4222,4223,4224,4225,4226,4227,4228,4229,4230,4231,4232,4233,4234,4235,4236,4237,4238,4239,4240,4241,4242,4243,4244,4245,4246,4247,4248,4249,4250,4251,4252,4253,4254,4255,4256,4257,4258,4259,4260,4261,4262,4263,4264,4265,4266,4267,4268,4269,4270,4271,4272,4273,4274,4275,4276,4277,4278,4279,4280,4281,4282,4283,4284,4285,4286,4287,4288,4289,4290,4291,4292,4293,4294,4295,4296,4297,4298,4299,4300,4301,4302,4303,4304,4305,4306,4307,4308,4309,4310,4311,4312,4313,4314,4315,4316,4317,4318,4319,4320,4321,4322,4323,4324,4325,4326,4327,4328,4329,4330,4331,4332,4333,4334,4335,4336,4337,4338,4339,4340,4341,4342,4343,4344,4345,4346,4347,4348,4349,4350,4351,4352,4353,4354,4355,4356,4357,4358,4359,4360,4361,4362,4363,4364,4365,4366,4367,4368,4369,4370,4371,4372,4373,4374,4375,4376,4377,4378,4379,4380,4381,4382,4383,4384,4385,4386,4387,4388,4389,4390,4391,4392,4393,4394,4395,4396,4397,4398,4399,4400,4401,4402,4403,4404,4405,4406,4407,4408,4409,4410,4411,4412,4413,4414,4415,4416,4417,4418,4419,4420,4421,4422,4423,4424,4425,4426,4427,4428,4429,4430,4431,4432,4433,4434,4435,4436,4437,4438,4439,4440,4441,4442,4443,4444,4445,4446,4447,4448,4449,4450,4451,4452,4453,4454,4455,4456,4457,4458,4459,4460,4461,4462,4463,4464,4465,4466,4467,4468,4469,4470,4471,4472,4473,4474,4475,4476,4477,4478,4479,4480,4481,4482,4483,4484,4485,4486,4487,4488,4489,4490,4491,4492,4493,4494,4495,4496,4497,4498,4499,4500,4501,4502,4503,4504,4505,4506,4507,4508,4509,4510,4511,4512,4513,4514,4515,4516,4517,4518,4519,4520,4521,4522,4523,4524,4525,4526,4527,4528,4529,4530,4531,4532,4533,4534,4535,4536,4537,4538,4539,4540,4541,4542,4543,4544,4545,4546,4547,4548,4549,4550,4551,4552,4553,4554,4555,4556,4557,4558,4559,4560,4561,4562,4563,4564,4565,4566,4567,4568,4569,4570,4571,4572,4573,4574,4575,4576,4577,4578,4579,4580,4581,4582,4583,4584,4585,4586,4587,4588,4589,4590,4591,4592,4593,4594,4595,4596,4597,4598,4599,4600,4601,4602,4603,4604,4605,4606,4607,4608,4609,4610,4611,4612,4613,4614,4615,4616,4617,4618,4619,4620,4621,4622,4623,4624,4625,4626,4627,4628,4629,4630,4631,4632,4633,4634,4635,4636,4637,4638,4639,4640,4641,4642,4643,4644,4645,4646,4647,4648,4649,4650,4651,4652,4653,4654,4655,4656,4657,4658,4659,4660,4661,4662,4663,4664,4665,4666,4667,4668,4669,4670,4671,4672,4673,4674,4675,4676,4677,4678,4679,4680,4681,4682,4683,4684,4685,4686,4687,4688,4689,4690,4691,4692,4693,4694,4695,4696,4697,4698,4699,4700,4701,4702,4703,4704,4705,4706,4707,4708,4709,4710,4711,4712,4713,4714,4715,4716,4717,4718,4719,4720,4721,4722,4723,4724,4725,4726,4727,4728,4729,4730,4731,4732,4733,4734,4735,4736,4737,4738,4739,4740,4741,4742,4743,4744,4745,4746,4747,4748,4749,4750,4751,4752,4753,4754,4755,4756,4757,4758,4759,4760,4761,4762,4763,4764,4765,4766,4767,4768,4769,4770,4771,4772,4773,4774,4775,4776,4777,4778,4779,4780,4781,4782,4783,4784,4785,4786,4787,4788,4789,4790,4791,4792,4793,4794,4795,4796,4797,4798,4799,4800,4801,4802,4803,4804,4805,4806,4807,4808,4809,4810,4811,4812,4813,4814,4815,4816,4817,4818,4819,4820,4821,4822,4823,4824,4825,4826,4827,4828,4829,4830,4831,4832,4833,4834,4835,4836,4837,4838,4839,4840,4841,4842,4843,4844,4845,4846,4847,4848,4849,4850,4851,4852,4853,4854,4855,4856,4857,4858,4859,4860,4861,4862,4863,4864,4865,4866,4867,4868,4869,4870,4871,4872,4873,4874,4875,4876,4877,4878,4879,4880,4881,4882,4883,4884,4885,4886,4887,4888,4889,4890,4891,4892,4893,4894,4895,4896,4897,4898,4899,4900,4901,4902,4903,4904,4905,4906,4907,4908,4909,4910,4911,4912,4913,4914,4915,4916,4917,4918,4919,4920,4921,4922,4923,4924,4925,4926,4927,4928,4929,4930,4931,4932,4933,4934,4935,4936,4937,4938,4939,4940,4941,4942,4943,4944,4945,4946,4947,4948,4949,4950,4951,4952,4953,4954,4955,4956,4957,4958,4959,4960,4961,4962,4963,4964,4965,4966,4967,4968,4969,4970,4971,4972,4973,4974,4975,4976,4977,4978,4979,4980,4981,4982,4983,4984,4985,4986,4987,4988,4989,4990,4991,4992,4993,4994,4995,4996,4997,4998,4999,5000,5001,5002,5003,5004,5005,5006,5007,5008,5009,5010,5011,5012,5013,5014,5015,5016,5017,5018,5019,5020,5021,5022,5023,5024,5025,5026,5027,5028,5029,5030,5031,5032,5033,5034,5035,5036,5037,5038,5039,5040,5041,5042,5043,5044,5045,5046,5047,5048,5049,5050,5051,5052,5053,5054,5055,5056,5057,5058,5059,5060,5061,5062,5063,5064,5065,5066,5067,5068,5069,5070,5071,5072,5073,5074,5075,5076,5077,5078,5079,5080,5081,5082,5083,5084,5085,5086,5087,5088,5089,5090,5091,5092,5093,5094,5095,5096,5097,5098,5099,5100,5101,5102,5103,5104,5105,5106,5107,5108,5109,5110,5111,5112,5113,5114,5115,5116,5117,5118,5119,5120,5121,5122,5123,5124,5125,5126,5127,5128,5129,5130,5131,5132,5133,5134,5135,5136,5137,5138,5139,5140,5141,5142,5143,5144,5145,5146,5147,5148,5149,5150,5151,5152,5153,5154,5155,5156,5157,5158,5159,5160,5161,5162,5163,5164,5165,5166,5167,5168,5169,5170,5171,5172,5173,5174,5175,5176,5177,5178,5179,5180,5181,5182,5183,5184,5185,5186,5187,5188,5189,5190,5191,5192,5193,5194,5195,5196,5197,5198,5199,5200,5201,5202,5203,5204,5205,5206,5207,5208,5209,5210,5211,5212,5213,5214,5215,5216,5217,5218,5219,5220,5221,5222,5223,5224,5225,5226,5227,5228,5229,5230,5231,5232,5233,5234,5235,5236,5237,5238,5239,5240,5241,5242,5243,5244,5245,5246,5247,5248,5249,5250,5251,5252,5253,5254,5255,5256,5257,5258,5259,5260,5261,5262,5263,5264,5265,5266,5267,5268,5269,5270,5271,5272,5273,5274,5275,5276,5277,5278,5279,5280,5281,5282,5283,5284,5285,5286,5287,5288,5289,5290,5291,5292,5293,5294,5295,5296,5297,5298,5299,5300,5301,5302,5303,5304,5305,5306,5307,5308,5309,5310,5311,5312,5313,5314,5315,5316,5317,5318,5319,5320,5321,5322,5323,5324,5325,5326,5327,5328,5329,5330,5331,5332,5333,5334,5335,5336,5337,5338,5339,5340,5341,5342,5343,5344,5345,5346,5347,5348,5349,5350,5351,5352,5353,5354,5355,5356,5357,5358,5359,5360,5361,5362,5363,5364,5365,5366,5367,5368,5369,5370,5371,5372,5373,5374,5375,5376,5377,5378,5379,5380,5381,5382,5383,5384,5385,5386,5387,5388,5389,5390,5391,5392,5393,5394,5395,5396,5397,5398,5399,5400,5401,5402,5403,5404,5405,5406,5407,5408,5409,5410,5411,5412,5413,5414,5415,5416,5417,5418,5419,5420,5421,5422,5423,5424,5425,5426,5427,5428,5429,5430,5431,5432,5433,5434,5435,5436,5437,5438,5439,5440,5441,5442,5443,5444,5445,5446,5447,5448,5449,5450,5451,5452,5453,5454,5455,5456,5457,5458,5459,5460,5461,5462,5463,5464,5465,5466,5467,5468,5469,5470,5471,5472,5473,5474,5475,5476,5477,5478,5479,5480,5481,5482,5483,5484,5485,5486,5487,5488,5489,5490,5491,5492,5493,5494,5495,5496,5497,5498,5499,5500,5501,5502,5503,5504,5505,5506,5507,5508,5509,5510,5511,5512,5513,5514,5515,5516,5517,5518,5519,5520,5521,5522,5523,5524,5525,5526,5527,5528,5529,5530,5531,5532,5533,5534,5535,5536,5537,5538,5539,5540,5541,5542,5543,5544,5545,5546,5547,5548,5549,5550,5551,5552,5553,5554,5555,5556,5557,5558,5559,5560,5561,5562,5563,5564,5565,5566,5567,5568,5569,5570,5571,5572,5573,5574,5575,5576,5577,5578,5579,5580,5581,5582,5583,5584,5585,5586,5587,5588,5589,5590,5591,5592,5593,5594,5595,5596,5597,5598,5599,5600,5601,5602,5603,5604,5605,5606,5607,5608,5609,5610,5611,5612,5613,5614,5615,5616,5617,5618,5619,5620,5621,5622,5623,5624,5625,5626,5627,5628,5629,5630,5631,5632,5633,5634,5635,5636,5637,5638,5639,5640,5641,5642,5643,5644,5645,5646,5647,5648,5649,5650,5651,5652,5653,5654,5655,5656,5657,5658,5659,5660,5661,5662,5663,5664,5665,5666,5667,5668,5669,5670,5671,5672,5673,5674,5675,5676,5677,5678,5679,5680,5681,5682,5683,5684,5685,5686,5687,5688,5689,5690,5691,5692,5693,5694,5695,5696,5697,5698,5699,5700,5701,5702,5703,5704,5705,5706,5707,5708,5709,5710,5711,5712,5713,5714,5715,5716,5717,5718,5719,5720,5721,5722,5723,5724,5725,5726,5727,5728,5729,5730,5731,5732,5733,5734,5735,5736,5737,5738,5739,5740,5741,5742,5743,5744,5745,5746,5747,5748,5749,5750,5751,5752,5753,5754,5755,5756,5757,5758,5759,5760,5761,5762,5763,5764,5765,5766,5767,5768,5769,5770,5771,5772,5773,5774,5775,5776,5777,5778,5779,5780,5781,5782,5783,5784,5785,5786,5787,5788,5789,5790,5791,5792,5793,5794,5795,5796,5797,5798,5799,5800,5801,5802,5803,5804,5805,5806,5807,5808,5809,5810,5811,5812,5813,5814,5815,5816,5817,5818,5819,5820,5821,5822,5823,5824,5825,5826,5827,5828,5829,5830,5831,5832,5833,5834,5835,5836,5837,5838,5839,5840,5841,5842,5843,5844,5845,5846,5847,5848,5849,5850,5851,5852,5853,5854,5855,5856,5857,5858,5859,5860,5861,5862,5863,5864,5865,5866,5867,5868,5869,5870,5871,5872,5873,5874,5875,5876,5877,5878,5879,5880,5881,5882,5883,5884,5885,5886,5887,5888,5889,5890,5891,5892,5893,5894,5895,5896,5897,5898,5899,5900,5901,5902,5903,5904,5905,5906,5907,5908,5909,5910,5911,5912,5913,5914,5915,5916,5917,5918,5919,5920,5921,5922,5923,5924,5925,5926,5927,5928,5929,5930,5931,5932,5933,5934,5935,5936,5937,5938,5939,5940,5941,5942,5943,5944,5945,5946,5947,5948,5949,5950,5951,5952,5953,5954,5955,5956,5957,5958,5959,5960,5961,5962,5963,5964,5965,5966,5967,5968,5969,5970,5971,5972,5973,5974,5975,5976,5977,5978,5979,5980,5981,5982,5983,5984,5985,5986,5987,5988,5989,5990,5991,5992,5993,5994,5995,5996,5997,5998,5999,6000,6001,6002,6003,6004,6005,6006,6007,6008,6009,6010,6011,6012,6013,6014,6015,6016,6017,6018,6019,6020,6021,6022,6023,6024,6025,6026,6027,6028,6029,6030,6031,6032,6033,6034,6035,6036,6037,6038,6039,6040,6041,6042,6043,6044,6045,6046,6047,6048,6049,6050,6051,6052,6053,6054,6055,6056,6057,6058,6059,6060,6061,6062,6063,6064,6065,6066,6067,6068,6069,6070,6071,6072,6073,6074,6075,6076,6077,6078,6079,6080,6081,6082,6083,6084,6085,6086,6087,6088,6089,6090,6091,6092,6093,6094,6095,6096,6097,6098,6099,6100,6101,6102,6103,6104,6105,6106,6107,6108,6109,6110,6111,6112,6113,6114,6115,6116,6117,6118,6119,6120,6121,6122,6123,6124,6125,6126,6127,6128,6129,6130,6131,6132,6133,6134,6135,6136,6137,6138,6139,6140,6141,6142,6143,6144,6145,6146,6147,6148,6149,6150,6151,6152,6153,6154,6155,6156,6157,6158,6159,6160,6161,6162,6163,6164,6165,6166,6167,6168,6169,6170,6171,6172,6173,6174,6175,6176,6177,6178,6179,6180,6181,6182,6183,6184,6185,6186,6187,6188,6189,6190,6191,6192,6193,6194,6195,6196,6197,6198,6199,6200,6201,6202,6203,6204,6205,6206,6207,6208,6209,6210,6211,6212,6213,6214,6215,6216,6217,6218,6219,6220,6221,6222,6223,6224,6225,6226,6227,6228,6229,6230,6231,6232,6233,6234,6235,6236,6237,6238,6239,6240,6241,6242,6243,6244,6245,6246,6247,6248,6249,6250,6251,6252,6253,6254,6255,6256,6257,6258,6259,6260,6261,6262,6263,6264,6265,6266,6267,6268,6269,6270,6271,6272,6273,6274,6275,6276,6277,6278,6279,6280,6281,6282,6283,6284,6285,6286,6287,6288,6289,6290,6291,6292,6293,6294,6295,6296,6297,6298,6299,6300,6301,6302,6303,6304,6305,6306,6307,6308,6309,6310,6311,6312,6313,6314,6315,6316,6317,6318,6319,6320,6321,6322,6323,6324,6325,6326,6327,6328,6329,6330,6331,6332,6333,6334,6335,6336,6337,6338,6339,6340,6341,6342,6343,6344,6345,6346,6347,6348,6349,6350,6351,6352,6353,6354,6355,6356,6357,6358,6359,6360,6361,6362,6363,6364,6365,6366,6367,6368,6369,6370,6371,6372,6373,6374,6375,6376,6377,6378,6379,6380,6381,6382,6383,6384,6385,6386,6387,6388,6389,6390,6391,6392,6393,6394,6395,6396,6397,6398,6399,6400,6401,6402,6403,6404,6405,6406,6407,6408,6409,6410,6411,6412,6413,6414,6415,6416,6417,6418,6419,6420,6421,6422,6423,6424,6425,6426,6427,6428,6429,6430,6431,6432,6433,6434,6435,6436,6437,6438,6439,6440,6441,6442,6443,6444,6445,6446,6447,6448,6449,6450,6451,6452,6453,6454,6455,6456,6457,6458,6459,6460,6461,6462,6463,6464,6465,6466,6467,6468,6469,6470,6471,6472,6473,6474,6475,6476,6477,6478,6479,6480,6481,6482,6483,6484,6485,6486,6487,6488,6489,6490,6491,6492,6493,6494,6495,6496,6497,6498,6499,6500,6501,6502,6503,6504,6505,6506,6507,6508,6509,6510,6511,6512,6513,6514,6515,6516,6517,6518,6519,6520,6521,6522,6523,6524,6525,6526,6527,6528,6529,6530,6531,6532,6533,6534,6535,6536,6537,6538,6539,6540,6541,6542,6543,6544,6545,6546,6547,6548,6549,6550,6551,6552,6553,6554,6555,6556,6557,6558,6559,6560,6561,6562,6563,6564,6565,6566,6567,6568,6569,6570,6571,6572,6573,6574,6575,6576,6577,6578,6579,6580,6581,6582,6583,6584,6585,6586,6587,6588,6589,6590,6591,6592,6593,6594,6595,6596,6597,6598,6599,6600,6601,6602,6603,6604,6605,6606,6607,6608,6609,6610,6611,6612,6613,6614,6615,6616,6617,6618,6619,6620,6621,6622,6623,6624,6625,6626,6627,6628,6629,6630,6631,6632,6633,6634,6635,6636,6637,6638,6639,6640,6641,6642,6643,6644,6645,6646,6647,6648,6649,6650,6651,6652,6653,6654,6655,6656,6657,6658,6659,6660,6661,6662,6663,6664,6665,6666,6667,6668,6669,6670,6671,6672,6673,6674,6675,6676,6677,6678,6679,6680,6681,6682,6683,6684,6685,6686,6687,6688,6689,6690,6691,6692,6693,6694,6695,6696,6697,6698,6699,6700,6701,6702,6703,6704,6705,6706,6707,6708,6709,6710,6711,6712,6713,6714,6715,6716,6717,6718,6719,6720,6721,6722,6723,6724,6725,6726,6727,6728,6729,6730,6731,6732,6733,6734,6735,6736,6737,6738,6739,6740,6741,6742,6743,6744,6745,6746,6747,6748,6749,6750,6751,6752,6753,6754,6755,6756,6757,6758,6759,6760,6761,6762,6763,6764,6765,6766,6767,6768,6769,6770,6771,6772,6773,6774,6775,6776,6777,6778,6779,6780,6781,6782,6783,6784,6785,6786,6787,6788,6789,6790,6791,6792,6793,6794,6795,6796,6797,6798,6799,6800,6801,6802,6803,6804,6805,6806,6807,6808,6809,6810,6811,6812,6813,6814,6815,6816,6817,6818,6819,6820,6821,6822,6823,6824,6825,6826,6827,6828,6829,6830,6831,6832,6833,6834,6835,6836,6837,6838,6839,6840,6841,6842,6843,6844,6845,6846,6847,6848,6849,6850,6851,6852,6853,6854,6855,6856,6857,6858,6859,6860,6861,6862,6863,6864,6865,6866,6867,6868,6869,6870,6871,6872,6873,6874,6875,6876,6877,6878,6879,6880,6881,6882,6883,6884,6885,6886,6887,6888,6889,6890,6891,6892,6893,6894,6895,6896,6897,6898,6899,6900,6901,6902,6903,6904,6905,6906,6907,6908,6909,6910,6911,6912,6913,6914,6915,6916,6917,6918,6919,6920,6921,6922,6923,6924,6925,6926,6927,6928,6929,6930,6931,6932,6933,6934,6935,6936,6937,6938,6939,6940,6941,6942,6943,6944,6945,6946,6947,6948,6949,6950,6951,6952,6953,6954,6955,6956,6957,6958,6959,6960,6961,6962,6963,6964,6965,6966,6967,6968,6969,6970,6971,6972,6973,6974,6975,6976,6977,6978,6979,6980,6981,6982,6983,6984,6985,6986,6987,6988,6989,6990,6991,6992,6993,6994,6995,6996,6997,6998,6999,7000,7001,7002,7003,7004,7005,7006,7007,7008,7009,7010,7011,7012,7013,7014,7015,7016,7017,7018,7019,7020,7021,7022,7023,7024,7025,7026,7027,7028,7029,7030,7031,7032,7033,7034,7035,7036,7037,7038,7039,7040,7041,7042,7043,7044,7045,7046,7047,7048,7049,7050,7051,7052,7053,7054,7055,7056,7057,7058,7059,7060,7061,7062,7063,7064,7065,7066,7067,7068,7069,7070,7071,7072,7073,7074,7075,7076,7077,7078,7079,7080,7081,7082,7083,7084,7085,7086,7087,7088,7089,7090,7091,7092,7093,7094,7095,7096,7097,7098,7099,7100,7101,7102,7103,7104,7105,7106,7107,7108,7109,7110,7111,7112,7113,7114,7115,7116,7117,7118,7119,7120,7121,7122,7123,7124,7125,7126,7127,7128,7129,7130,7131,7132,7133,7134,7135,7136,7137,7138,7139,7140,7141,7142,7143,7144,7145,7146,7147,7148,7149,7150,7151,7152,7153,7154,7155,7156,7157,7158,7159,7160,7161,7162,7163,7164,7165,7166,7167,7168,7169,7170,7171,7172,7173,7174,7175,7176,7177,7178,7179,7180,7181,7182,7183,7184,7185,7186,7187,7188,7189,7190,7191,7192,7193,7194,7195,7196,7197,7198,7199,7200,7201,7202,7203,7204,7205,7206,7207,7208,7209,7210,7211,7212,7213,7214,7215,7216,7217,7218,7219,7220,7221,7222,7223,7224,7225,7226,7227,7228,7229,7230,7231,7232,7233,7234,7235,7236,7237,7238,7239,7240,7241,7242,7243,7244,7245,7246,7247,7248,7249,7250,7251,7252,7253,7254,7255,7256,7257,7258,7259,7260,7261,7262,7263,7264,7265,7266,7267,7268,7269,7270,7271,7272,7273,7274,7275,7276,7277,7278,7279,7280,7281,7282,7283,7284,7285,7286,7287,7288,7289,7290,7291,7292,7293,7294,7295,7296,7297,7298,7299,7300,7301,7302,7303,7304,7305,7306,7307,7308,7309,7310,7311,7312,7313,7314,7315,7316,7317,7318,7319,7320,7321,7322,7323,7324,7325,7326,7327,7328,7329,7330,7331,7332,7333,7334,7335,7336,7337,7338,7339,7340,7341,7342,7343,7344,7345,7346,7347,7348,7349,7350,7351,7352,7353,7354,7355,7356,7357,7358,7359,7360,7361,7362,7363,7364,7365,7366,7367,7368,7369,7370,7371,7372,7373,7374,7375,7376,7377,7378,7379,7380,7381,7382,7383,7384,7385,7386,7387,7388,7389,7390,7391,7392,7393,7394,7395,7396,7397,7398,7399,7400,7401,7402,7403,7404,7405,7406,7407,7408,7409,7410,7411,7412,7413,7414,7415,7416,7417,7418,7419,7420,7421,7422,7423,7424,7425,7426,7427,7428,7429,7430,7431,7432,7433,7434,7435,7436,7437,7438,7439,7440,7441,7442,7443,7444,7445,7446,7447,7448,7449,7450,7451,7452,7453,7454,7455,7456,7457,7458,7459,7460,7461,7462,7463,7464,7465,7466,7467,7468,7469,7470,7471,7472,7473,7474,7475,7476,7477,7478,7479,7480,7481,7482,7483,7484,7485,7486,7487,7488,7489,7490,7491,7492,7493,7494,7495,7496,7497,7498,7499,7500,7501,7502,7503,7504,7505,7506,7507,7508,7509,7510,7511,7512,7513,7514,7515,7516,7517,7518,7519,7520,7521,7522,7523,7524,7525,7526,7527,7528,7529,7530,7531,7532,7533,7534,7535,7536,7537,7538,7539,7540,7541,7542,7543,7544,7545,7546,7547,7548,7549,7550,7551,7552,7553,7554,7555,7556,7557,7558,7559,7560,7561,7562,7563,7564,7565,7566,7567,7568,7569,7570,7571,7572,7573,7574,7575,7576,7577,7578,7579,7580,7581,7582,7583,7584,7585,7586,7587,7588,7589,7590,7591,7592,7593,7594,7595,7596,7597,7598,7599,7600,7601,7602,7603,7604,7605,7606,7607,7608,7609,7610,7611,7612,7613,7614,7615,7616,7617,7618,7619,7620,7621,7622,7623,7624,7625,7626,7627,7628,7629,7630,7631,7632,7633,7634,7635,7636,7637,7638,7639,7640,7641,7642,7643,7644,7645,7646,7647,7648,7649,7650,7651,7652,7653,7654,7655,7656,7657,7658,7659,7660,7661,7662,7663,7664,7665,7666,7667,7668,7669,7670,7671,7672,7673,7674,7675,7676,7677,7678,7679,7680,7681,7682,7683,7684,7685,7686,7687,7688,7689,7690,7691,7692,7693,7694,7695,7696,7697,7698,7699,7700,7701,7702,7703,7704,7705,7706,7707,7708,7709,7710,7711,7712,7713,7714,7715,7716,7717,7718,7719,7720,7721,7722,7723,7724,7725,7726,7727,7728,7729,7730,7731,7732,7733,7734,7735,7736,7737,7738,7739,7740,7741,7742,7743,7744,7745,7746,7747,7748,7749,7750,7751,7752,7753,7754,7755,7756,7757,7758,7759,7760,7761,7762,7763,7764,7765,7766,7767,7768,7769,7770,7771,7772,7773,7774,7775,7776,7777,7778,7779,7780,7781,7782,7783,7784,7785,7786,7787,7788,7789,7790,7791,7792,7793,7794,7795,7796,7797,7798,7799,7800,7801,7802,7803,7804,7805,7806,7807,7808,7809,7810,7811,7812,7813,7814,7815,7816,7817,7818,7819,7820,7821,7822,7823,7824,7825,7826,7827,7828,7829,7830,7831,7832,7833,7834,7835,7836,7837,7838,7839,7840,7841,7842,7843,7844,7845,7846,7847,7848,7849,7850,7851,7852,7853,7854,7855,7856,7857,7858,7859,7860,7861,7862,7863,7864,7865,7866,7867,7868,7869,7870,7871,7872,7873,7874,7875,7876,7877,7878,7879,7880,7881,7882,7883,7884,7885,7886,7887,7888,7889,7890,7891,7892,7893,7894,7895,7896,7897,7898,7899,7900,7901,7902,7903,7904,7905,7906,7907,7908,7909,7910,7911,7912,7913,7914,7915,7916,7917,7918,7919,7920,7921,7922,7923,7924,7925,7926,7927,7928,7929,7930,7931,7932,7933,7934,7935,7936,7937,7938,7939,7940,7941,7942,7943,7944,7945,7946,7947,7948,7949,7950,7951,7952,7953,7954,7955,7956,7957,7958,7959,7960,7961,7962,7963,7964,7965,7966,7967,7968,7969,7970,7971,7972,7973,7974,7975,7976,7977,7978,7979,7980,7981,7982,7983,7984,7985,7986,7987,7988,7989,7990,7991,7992,7993,7994,7995,7996,7997,7998,7999,8000,8001,8002,8003,8004,8005,8006,8007,8008,8009,8010,8011,8012,8013,8014,8015,8016,8017,8018,8019,8020,8021,8022,8023,8024,8025,8026,8027,8028,8029,8030,8031,8032,8033,8034,8035,8036,8037,8038,8039,8040,8041,8042,8043,8044,8045,8046,8047,8048,8049,8050,8051,8052,8053,8054,8055,8056,8057,8058,8059,8060,8061,8062,8063,8064,8065,8066,8067,8068,8069,8070,8071,8072,8073,8074,8075,8076,8077,8078,8079,8080,8081,8082,8083,8084,8085,8086,8087,8088,8089,8090,8091,8092,8093,8094,8095,8096,8097,8098,8099,8100,8101,8102,8103,8104,8105,8106,8107,8108,8109,8110,8111,8112,8113,8114,8115,8116,8117,8118,8119,8120,8121,8122,8123,8124,8125,8126,8127,8128,8129,8130,8131,8132,8133,8134,8135,8136,8137,8138,8139,8140,8141,8142,8143,8144,8145,8146,8147,8148,8149,8150,8151,8152,8153,8154,8155,8156,8157,8158,8159,8160,8161,8162,8163,8164,8165,8166,8167,8168,8169,8170,8171,8172,8173,8174,8175,8176,8177,8178,8179,8180,8181,8182,8183,8184,8185,8186,8187,8188,8189,8190,8191,8192,8193,8194,8195,8196,8197,8198,8199,8200,8201,8202,8203,8204,8205,8206,8207,8208,8209,8210,8211,8212,8213,8214,8215,8216,8217,8218,8219,8220,8221,8222,8223,8224,8225,8226,8227,8228,8229,8230,8231,8232,8233,8234,8235,8236,8237,8238,8239,8240,8241,8242,8243,8244,8245,8246,8247,8248,8249,8250,8251,8252,8253,8254,8255,8256,8257,8258,8259,8260,8261,8262,8263,8264,8265,8266,8267,8268,8269,8270,8271,8272,8273,8274,8275,8276,8277,8278,8279,8280,8281,8282,8283,8284,8285,8286,8287,8288,8289,8290,8291,8292,8293,8294,8295,8296,8297,8298,8299,8300,8301,8302,8303,8304,8305,8306,8307,8308,8309,8310,8311,8312,8313,8314,8315,8316,8317,8318,8319,8320,8321,8322,8323,8324,8325,8326,8327,8328,8329,8330,8331,8332,8333,8334,8335,8336,8337,8338,8339,8340,8341,8342,8343,8344,8345,8346,8347,8348,8349,8350,8351,8352,8353,8354,8355,8356,8357,8358,8359,8360,8361,8362,8363,8364,8365,8366,8367,8368,8369,8370,8371,8372,8373,8374,8375,8376,8377,8378,8379,8380,8381,8382,8383,8384,8385,8386,8387,8388,8389,8390,8391,8392,8393,8394,8395,8396,8397,8398,8399,8400,8401,8402,8403,8404,8405,8406,8407,8408,8409,8410,8411,8412,8413,8414,8415,8416,8417,8418,8419,8420,8421,8422,8423,8424,8425,8426,8427,8428,8429,8430,8431,8432,8433,8434,8435,8436,8437,8438,8439,8440,8441,8442,8443,8444,8445,8446,8447,8448,8449,8450,8451,8452,8453,8454,8455,8456,8457,8458,8459,8460,8461,8462,8463,8464,8465,8466,8467,8468,8469,8470,8471,8472,8473,8474,8475,8476,8477,8478,8479,8480,8481,8482,8483,8484,8485,8486,8487,8488,8489,8490,8491,8492,8493,8494,8495,8496,8497,8498,8499,8500,8501,8502,8503,8504,8505,8506,8507,8508,8509,8510,8511,8512,8513,8514,8515,8516,8517,8518,8519,8520,8521,8522,8523,8524,8525,8526,8527,8528,8529,8530,8531,8532,8533,8534,8535,8536,8537,8538,8539,8540,8541,8542,8543,8544,8545,8546,8547,8548,8549,8550,8551,8552,8553,8554,8555,8556,8557,8558,8559,8560,8561,8562,8563,8564,8565,8566,8567,8568,8569,8570,8571,8572,8573,8574,8575,8576,8577,8578,8579,8580,8581,8582,8583,8584,8585,8586,8587,8588,8589,8590,8591,8592,8593,8594,8595,8596,8597,8598,8599,8600,8601,8602,8603,8604,8605,8606,8607,8608,8609,8610,8611,8612,8613,8614,8615,8616,8617,8618,8619,8620,8621,8622,8623,8624,8625,8626,8627,8628,8629,8630,8631,8632,8633,8634,8635,8636,8637,8638,8639,8640,8641,8642,8643,8644,8645,8646,8647,8648,8649,8650,8651,8652,8653,8654,8655,8656,8657,8658,8659,8660,8661,8662,8663,8664,8665,8666,8667,8668,8669,8670,8671,8672,8673,8674,8675,8676,8677,8678,8679,8680,8681,8682,8683,8684,8685,8686,8687,8688,8689,8690,8691,8692,8693,8694,8695,8696,8697,8698,8699,8700,8701,8702,8703,8704,8705,8706,8707,8708,8709,8710,8711,8712,8713,8714,8715,8716,8717,8718,8719,8720,8721,8722,8723,8724,8725,8726,8727,8728,8729,8730,8731,8732,8733,8734,8735,8736,8737,8738,8739,8740,8741,8742,8743,8744,8745,8746,8747,8748,8749,8750,8751,8752,8753,8754,8755,8756,8757,8758,8759,8760,8761,8762,8763,8764,8765,8766,8767,8768,8769,8770,8771,8772,8773,8774,8775,8776,8777,8778,8779,8780,8781,8782,8783,8784,8785,8786,8787,8788,8789,8790,8791,8792,8793,8794,8795,8796,8797,8798,8799,8800,8801,8802,8803,8804,8805,8806,8807,8808,8809,8810,8811,8812,8813,8814,8815,8816,8817,8818,8819,8820,8821,8822,8823,8824,8825,8826,8827,8828,8829,8830,8831,8832,8833,8834,8835,8836,8837,8838,8839,8840,8841,8842,8843,8844,8845,8846,8847,8848,8849,8850,8851,8852,8853,8854,8855,8856,8857,8858,8859,8860,8861,8862,8863,8864,8865,8866,8867,8868,8869,8870,8871,8872,8873,8874,8875,8876,8877,8878,8879,8880,8881,8882,8883,8884,8885,8886,8887,8888,8889,8890,8891,8892,8893,8894,8895,8896,8897,8898,8899,8900,8901,8902,8903,8904,8905,8906,8907,8908,8909,8910,8911,8912,8913,8914,8915,8916,8917,8918,8919,8920,8921,8922,8923,8924,8925,8926,8927,8928,8929,8930,8931,8932,8933,8934,8935,8936,8937,8938,8939,8940,8941,8942,8943,8944,8945,8946,8947,8948,8949,8950,8951,8952,8953,8954,8955,8956,8957,8958,8959,8960,8961,8962,8963,8964,8965,8966,8967,8968,8969,8970,8971,8972,8973,8974,8975,8976,8977,8978,8979,8980,8981,8982,8983,8984,8985,8986,8987,8988,8989,8990,8991,8992,8993,8994,8995,8996,8997,8998,8999,9000,9001,9002,9003,9004,9005,9006,9007,9008,9009,9010,9011,9012,9013,9014,9015,9016,9017,9018,9019,9020,9021,9022,9023,9024,9025,9026,9027,9028,9029,9030,9031,9032,9033,9034,9035,9036,9037,9038,9039,9040,9041,9042,9043,9044,9045,9046,9047,9048,9049,9050,9051,9052,9053,9054,9055,9056,9057,9058,9059,9060,9061,9062,9063,9064,9065,9066,9067,9068,9069,9070,9071,9072,9073,9074,9075,9076,9077,9078,9079,9080,9081,9082,9083,9084,9085,9086,9087,9088,9089,9090,9091,9092,9093,9094,9095,9096,9097,9098,9099,9100,9101,9102,9103,9104,9105,9106,9107,9108,9109,9110,9111,9112,9113,9114,9115,9116,9117,9118,9119,9120,9121,9122,9123,9124,9125,9126,9127,9128,9129,9130,9131,9132,9133,9134,9135,9136,9137,9138,9139,9140,9141,9142,9143,9144,9145,9146,9147,9148,9149,9150,9151,9152,9153,9154,9155,9156,9157,9158,9159,9160,9161,9162,9163,9164,9165,9166,9167,9168,9169,9170,9171,9172,9173,9174,9175,9176,9177,9178,9179,9180,9181,9182,9183,9184,9185,9186,9187,9188,9189,9190,9191,9192,9193,9194,9195,9196,9197,9198,9199,9200,9201,9202,9203,9204,9205,9206,9207,9208,9209,9210,9211,9212,9213,9214,9215,9216,9217,9218,9219,9220,9221,9222,9223,9224,9225,9226,9227,9228,9229,9230,9231,9232,9233,9234,9235,9236,9237,9238,9239,9240,9241,9242,9243,9244,9245,9246,9247,9248,9249,9250,9251,9252,9253,9254,9255,9256,9257,9258,9259,9260,9261,9262,9263,9264,9265,9266,9267,9268,9269,9270,9271,9272,9273,9274,9275,9276,9277,9278,9279,9280,9281,9282,9283,9284,9285,9286,9287,9288,9289,9290,9291,9292,9293,9294,9295,9296,9297,9298,9299,9300,9301,9302,9303,9304,9305,9306,9307,9308,9309,9310,9311,9312,9313,9314,9315,9316,9317,9318,9319,9320,9321,9322,9323,9324,9325,9326,9327,9328,9329,9330,9331,9332,9333,9334,9335,9336,9337,9338,9339,9340,9341,9342,9343,9344,9345,9346,9347,9348,9349,9350,9351,9352,9353,9354,9355,9356,9357,9358,9359,9360,9361,9362,9363,9364,9365,9366,9367,9368,9369,9370,9371,9372,9373,9374,9375,9376,9377,9378,9379,9380,9381,9382,9383,9384,9385,9386,9387,9388,9389,9390,9391,9392,9393,9394,9395,9396,9397,9398,9399,9400,9401,9402,9403,9404,9405,9406,9407,9408,9409,9410,9411,9412,9413,9414,9415,9416,9417,9418,9419,9420,9421,9422,9423,9424,9425,9426,9427,9428,9429,9430,9431,9432,9433,9434,9435,9436,9437,9438,9439,9440,9441,9442,9443,9444,9445,9446,9447,9448,9449,9450,9451,9452,9453,9454,9455,9456,9457,9458,9459,9460,9461,9462,9463,9464,9465,9466,9467,9468,9469,9470,9471,9472,9473,9474,9475,9476,9477,9478,9479,9480,9481,9482,9483,9484,9485,9486,9487,9488,9489,9490,9491,9492,9493,9494,9495,9496,9497,9498,9499,9500,9501,9502,9503,9504,9505,9506,9507,9508,9509,9510,9511,9512,9513,9514,9515,9516,9517,9518,9519,9520,9521,9522,9523,9524,9525,9526,9527,9528,9529,9530,9531,9532,9533,9534,9535,9536,9537,9538,9539,9540,9541,9542,9543,9544,9545,9546,9547,9548,9549,9550,9551,9552,9553,9554,9555,9556,9557,9558,9559,9560,9561,9562,9563,9564,9565,9566,9567,9568,9569,9570,9571,9572,9573,9574,9575,9576,9577,9578,9579,9580,9581,9582,9583,9585,9586,9587,9588,9589,9590,9591,9592,9593,9594,9595,9596,9597,9598,9599,9600,9601,9602,9603,9604,9605,9606,9607,9608,9609,9610,9611,9612,9613,9614,9615,9616,9617,9618,9619,9620,9621,9622,9623,9624,9625,9626,9627,9628,9629,9630,9631,9632,9633,9634,9635,9636,9637,9638,9639,9640,9641,9642,9643,9644,9645,9646,9647,9648,9649,9650,9651,9652,9653,9654,9655,9656,9657,9658,9659,9660,9661,9662,9663,9664,9665,9666,9667,9668,9669,9670,9671,9672,9673,9674,9675,9676,9677,9678,9679,9680,9681,9682,9683,9684,9685,9686,9687,9688,9689,9690,9691,9692,9693,9694,9695,9696,9697,9698,9699,9700,9701,9702,9703,9704,9705,9706,9707,9708,9709,9710,9711,9712,9713,9714,9715,9716,9717,9718,9719,9720,9721,9722,9723,9724,9725,9726,9727,9728,9729,9730,9731,9732,9733,9734,9735,9736,9737,9738,9739,9740,9741,9742,9743,9744,9745,9746,9747,9748,9749,9750,9751,9752,9753,9754,9755,9756,9757,9758,9759,9760,9761,9762,9763,9764,9765,9766,9767,9768,9769,9770,9771,9772,9773,9774,9775,9776,9777,9778,9779,9780,9781,9782,9783,9784,9785,9786,9787,9789,9790,9791,9792,9793,9794,9795,9796,9797,9798,9799,9800,9801,9802,9803,9804,9805,9806,9807,9808,9809,9810,9811,9812,9813,9814,9815,9816,9817,9818,9819,9820,9821,9822,9823,9824,9825,9826,9827,9828,9829,9830,9831,9832,9833,9834,9835,9836,9837,9838,9839,9840,9841,9842,9843,9844,9845,9846,9847,9848,9849,9850,9851,9852,9853,9854,9855,9856,9857,9858,9859,9860,9861,9862,9863,9864,9865,9866,9867,9868,9869,9870,9871,9872,9873,9874,9875,9876,9877,9878,9879,9880,9881,9882,9883,9884,9885,9886,9887,9888,9889,9890,9891,9892,9893,9894,9896,9897,9898,9899,9900,9901,9902,9903,9904,9905,9906,9907,9908,9909,9910,9911,9912,9913,9914,9915,9916,9917,9918,9919,9920,9921,9922,9923,9924,9925,9926,9927,9928,9929,9930,9931,9932,9933,9934,9935,9936,9938,9939,9940,9941,9942,9943,9944,9945,9946,9947,9948,9949,9950,9951,9952,9953,9954,9955,9956,9957,9958,9959,9960,9961,9962,9963,9964,9965,9966,9967,9968,9969,9970,9971,9972,9973,9974,9975,9976,9977,9978,9979,9980,9981,9982,9983,9984,9985,9986,9987,9988,9989,9990,9991,9992,9993,9994,9995,9996,9997,9998,9999,10000,10001,10002,10003,10004,10005,10006,10007,10008,10009,10010,10011,10012,10013,10014,10015,10016,10017,10018,10019,10020,10021,10022,10023,10024,10025,10026,10028,10029,10030,10031,10032,10034,10035,10036,10037,10038,10039,10040,10041,10042,10043,10044,10046,10047,10048,10049,10050,10051,10052,10053,10054,10055,10056,10057,10058,10059,10060,10061,10062,10063,10064,10065,10066,10067,10068,10069,10070,10071,10072,10073,10074,10076,10077,10078,10079,10080,10081,10082,10083,10084,10085,10086,10087,10088,10089,10090,10091,10092,10093,10094,10095,10096,10097,10098,10099,10100,10101,10102,10103,10104,10106,10107,10108,10109,10110,10111,10112,10113,10114,10115,10116,10117,10118,10119,10120,10121,10122,10123,10124,10125,10126,10127,10128,10129,10130,10131,10132,10133,10134,10135,10136,10137,10138,10139,10140,10141,10142,10143,10144,10146,10147,10148,10149,10150,10151,10152,10153,10154,10155,10156,10157,10158,10159,10160,10161,10162,10163,10164,10165,10166,10168,10169,10170,10171,10172,10173,10174,10175,10176,10177,10178,10179,10180,10181,10182,10183,10184,10185,10186,10188,10189,10190,10191,10192,10193,10194,10195,10196,10197,10198,10200,10201,10202,10203,10204,10205,10206,10207,10208,10209,10210,10211,10212,10213,10214,10215,10216,10217,10218,10219,10220,10221,10222,10223,10224,10225,10226,10227,10228,10229,10230,10231,10232,10233,10234,10235,10236,10237,10238,10240,10241,10242,10243,10244,10245,10246,10247,10248,10249,10250,10251,10252,10253,10254,10255,10256,10257,10258,10259,10260,10261,10262,10263,10264,10265,10266,10267,10268,10269,10270,10271,10272,10273,10274,10275,10276,10277,10278,10279,10280,10281,10282,10283,10284,10285,10286,10287,10288,10290,10291,10292,10293,10294,10295,10296,10297,10298,10299,10300,10301,10302,10303,10304,10305,10306,10307,10308,10309,10310,10311,10312,10313,10314,10315,10316,10317,10318,10319,10320,10321,10322,10323,10324,10325,10326,10327,10328,10329,10330,10331,10332,10333,10334,10335,10336,10337,10338,10339,10340,10341,10342,10343,10344,10345,10346,10347,10348,10349,10350,10351,10352,10353,10354,10355,10357,10358,10359,10360,10361,10362,10363,10364,10365,10366,10367,10368,10369,10370,10371,10372,10373,10374,10375,10376,10377,10378,10379,10380,10382,10383,10384,10385,10386,10387,10388,10389,10390,10391,10392,10393,10394,10395,10396,10397,10398,10399,10400,10401,10402,10403,10404,10405,10406,10407,10408,10409,10410,10411,10413,10414,10415,10416,10417,10418,10419,10420,10421,10422,10424,10425,10426,10427,10428,10429,10430,10431,10432,10433,10434,10435,10436,10437,10438,10439,10440,10441,10442,10443,10444,10445,10446,10447,10448,10449,10450,10451,10452,10453,10454,10455,10456,10457,10458,10459,10460,10461,10462,10463,10464,10465,10466,10467,10468,10469,10470,10471,10473,10474,10475,10476,10477,10478,10479,10480,10481,10482,10483,10484,10486,10487,10488,10489,10490,10491,10492,10493,10494,10495,10496,10497,10498,10499,10500,10501,10502,10503,10504,10505,10506,10507,10508,10509,10510,10511,10512,10513,10514,10515,10516,10517,10518,10519,10520,10521,10522,10523,10524,10525,10526,10527,10528,10529,10530,10531,10532,10533,10534,10535,10536,10538,10539,10541,10542,10543,10544,10545,10546,10547,10548,10549,10550,10551,10552,10553,10554,10555,10556,10557,10558,10559,10560,10561,10562,10563,10564,10565,10566,10567,10568,10569,10570,10571,10572,10573,10574,10575,10576,10577,10578,10579,10580,10581,10582,10583,10585,10586,10587,10588,10589,10590,10591,10592,10593,10594,10595,10596,10597,10598,10599,10600,10601,10602,10603,10604,10605,10606,10607,10608,10609,10610,10611,10613,10615,10616,10617,10618,10619,10620,10621,10622,10623,10624,10625,10627,10628,10629,10630,10631,10632,10633,10634,10635,10636,10637,10638,10639,10641,10642,10643,10644,10645,10646,10647,10648,10650,10651,10652,10653,10655,10656,10657,10658,10659,10660,10661,10662,10663,10665,10666,10667,10668,10669,10670,10671,10672,10673,10674,10675,10676,10677,10678,10679,10680,10681,10682,10683,10684,10685,10687,10688,10689,10690,10691,10692,10693,10695,10697,10698,10699,10700,10702,10703,10704,10705,10706,10707,10709,10711,10712,10713,10715,10716,10717,10718,10719,10720,10721,10722,10723,10726,10727,10730,10731,10732,10733,10734,10736,10737,10738,10739,10741,10742,10743,10744,10745,10746,10747,10748,10749,10750,10751,10752,10754,10755,10756,10757,10758,10759,10760,10761,10762,10763,10764,10765,10766,10767,10768,10769,10770,10771,10772,10773,10774,10775,10776,10777,10778,10779,10780,10781,10782,10783,10784,10785,10786,10788,10791,10792,10793,10794,10795,10797,10798,10799,10800,10801,10802,10803,10804,10805,10806,10807,10808,10810,10811,10813,10814,10815,10816,10817,10818,10819,10820,10821,10822,10823,10824,10825,10826,10827,10828,10829,10830,10831,10832,10833,10834,10835,10836,10837,10838,10839,10840,10841,10842,10843,10845,10846,10847,10848,10849,10850,10851,10852,10853,10854,10855,10856,10857,10858,10859,10861,10862,10863,10864,10865,10866,10867,10868,10869,10870,10871,10872,10873,10874,10875,10876,10877,10878,10879,10880,10881,10882,10883,10884,10885,10886,10887,10888,10889,10890,10892,10893,10894,10895,10897,10898,10899,10900,10901,10902,10903,10904,10905,10906,10907,10908,10909,10910,10911,10913,10914,10915,10916,10917,10918,10919,10920,10921,10922,10923,10925,10926,10927,10928,10929,10930,10931,10932,10933,10935,10936,10937,10938,10939,10940,10942,10943,10944,10945,10946,10947,10949,10950,10951,10952,10953,10954,10956,10957,10958,10959,10960,10961,10962,10964,10965,10966,10967,10968,10969,10970,10971,10972,10973,10974,10975,10976,10978,10979,10980,10981,10983,10984,10985,10986,10989,10990,10991,10992,10993,10994,10995,10996,10997,10998,10999,11000,11001,11002,11003,11005,11006,11007,11008,11009,11011,11012,11013,11014,11015,11016,11017,11018,11019,11020,11021,11022,11023,11024,11025,11026,11027,11028,11029,11030,11031,11032,11034,11035,11036,11037,11038,11039,11040,11041,11042,11043,11045,11046,11047,11048,11049,11050,11051,11052,11053,11054,11055,11056,11057,11058,11059,11060,11061,11062,11063,11064,11065,11066,11067,11068,11069,11070,11071,11072,11073,11074,11075,11077,11078,11079,11080,11081,11082,11083,11084,11085,11086,11087,11088,11089,11090,11091,11092,11093,11094,11095,11096,11098,11099,11100,11101,11102,11103,11104,11105,11106,11107,11109,11110,11111,11112,11113,11114,11115,11117,11118,11120,11121,11122,11123,11126,11127,11128,11129,11131,11134,11135,11136,11137,11138,11139,11140,11141,11142,11143,11144,11145,11147,11148,11149,11150,11151,11152,11153,11154,11155,11156,11157,11159,11161,11163,11164,11165,11166,11167,11168,11169,11170,11171,11172,11174,11175,11176,11177,11178,11179,11180,11182,11184,11185,11186,11187,11189,11190,11191,11192,11193,11194,11195,11196,11199,11200,11201,11202,11203,11205,11207,11209,11210,11211,11212,11213,11214,11215,11216,11217,11218,11219,11220,11221,11223,11224,11225,11226,11227,11228,11229,11230,11231,11232,11233,11234,11235,11236,11238,11239,11240,11241,11243,11244,11247,11248,11249,11250,11252,11253,11254,11255,11256,11257,11258,11259,11260,11261,11262,11263,11264,11265,11266,11267,11268,11269,11270,11271,11272,11273,11274,11275,11276,11277,11278,11279,11280,11281,11282,11283,11284,11285,11286,11287,11288,11290,11291,11292,11293,11294,11295,11296,11297,11298,11299,11300,11301,11303,11304,11305,11306,11307,11308,11309,11310,11311,11312,11313,11314,11315,11316,11317,11318,11319,11320,11321,11322,11323,11324,11325,11328,11329,11331,11332,11334,11335,11336,11338,11339,11340,11341,11342,11343,11344,11345,11346,11347,11348,11349,11351,11352,11353,11354,11355,11356,11357,11358,11359,11360,11363,11364,11365,11366,11367,11368,11369,11370,11371,11373,11374,11375,11377,11379,11380,11381,11383,11384,11385,11388,11389,11390,11391,11392,11393,11394,11396,11397,11399,11400,11401,11402,11403,11404,11405,11406,11407,11408,11409,11410,11411,11413,11414,11415,11416,11417,11418,11419,11420,11423,11425,11426,11427,11428,11429,11432,11433,11434,11435,11436,11437,11438,11440,11441,11443,11445,11446,11447,11448,11449,11451,11452,11453,11455,11456,11457,11458,11459,11460,11461,11462,11463,11464,11465,11466,11468,11469,11470,11471,11472,11473,11475,11476,11477,11478,11479,11480,11481,11483,11484,11485,11486,11487,11488,11489,11491,11492,11493,11494,11496,11497,11498,11499,11500,11501,11502,11503,11505,11506,11507,11508,11509,11510,11512,11513,11515,11518,11519,11520,11522,11524,11525,11526,11527,11528,11532,11535,11537,11538,11539,11540,11542,11543,11545,11546,11547,11548,11549,11550,11551,11552,11553,11554,11555,11557,11558,11559,11560,11561,11562,11563,11565,11566,11567,11569,11570,11571,11572,11573,11574,11575,11576,11578,11579,11580,11581,11582,11583,11586,11587,11588,11589,11590,11592,11593,11594,11595,11596,11597,11598,11602,11604,11606,11607,11608,11609,11614,11615,11616,11617,11620,11621,11624,11625,11627,11628,11631,11632,11634,11636,11637,11638,11639,11641,11643,11644,11645,11646,11647,11648,11649,11651,11652,11653,11654,11655,11656,11658,11659,11660,11661,11662,11663,11664,11665,11666,11667,11668,11671,11672,11673,11675,11677,11678,11679,11680,11681,11683,11684,11686,11687,11689,11690,11691,11692,11693,11695,11696,11699,11700,11701,11702,11703,11705,11707,11708,11709,11710,11711,11712,11713,11714,11715,11716,11717,11718,11720,11721,11722,11724,11725,11726,11727,11728,11730,11731,11733,11735,11736,11737,11738,11739,11741,11742,11744,11745,11746,11747,11748,11749,11750,11751,11752,11753,11754,11755,11756,11757,11758,11759,11763,11764,11765,11766,11767,11768,11769,11770,11771,11774,11775,11776,11777,11778,11779,11781,11782,11783,11784,11785,11786,11787,11788,11789,11790,11791,11792,11794,11795,11796,11798,11799,11800,11802,11804,11805,11807,11808,11810,11811,11812,11813,11814,11816,11817,11818,11819,11821,11822,11823,11825,11826,11829,11830,11831,11832,11833,11834,11835,11836,11837,11838,11840,11841,11842,11843,11844,11845,11847,11849,11850,11851,11853,11854,11855,11856,11857,11858,11859,11860,11861,11862,11863,11864,11866,11868,11870,11872,11873,11874,11875,11877,11878,11880,11881,11882,11883,11884,11885,11886,11887,11888,11890,11892,11893,11894,11895,11896,11897,11898,11899,11900,11901,11902,11903,11904,11905,11906,11907,11910,11911,11912,11914,11915,11916,11917,11919,11920,11922,11925,11926,11927,11928,11929,11930,11931,11932,11933,11934,11936,11938,11939,11940,11942,11943,11944,11945,11946,11948,11949,11950,11952,11954,11957,11959,11960,11961,11962,11963,11964,11965,11966,11967,11968,11969,11973,11974,11975,11976,11977,11978,11980,11981,11983,11985,11988,11989,11990,11991,11993,11994,11995,11997,11999,12000,12001,12003,12004,12005,12006,12007,12008,12009,12012,12013,12014,12015,12016,12017,12018,12019,12023,12024,12026,12027,12028,12029,12030,12031,12032,12033,12035,12036,12037,12038,12039,12040,12041,12042,12044,12046,12047,12048,12049,12050,12051,12052,12053,12054,12055,12057,12058,12059,12061,12062,12063,12064,12065,12066,12067,12070,12071,12073,12074,12075,12077,12078,12079,12080,12082,12083,12085,12086,12087,12090,12092,12094,12096,12097,12098,12099,12100,12101,12102,12103,12104,12106,12107,12108,12109,12111,12112,12113,12116,12118,12119,12122,12124,12126,12127,12130,12131,12132,12133,12135,12137,12138,12140,12141,12142,12143,12144,12145,12148,12152,12154,12156,12157,12158,12159,12160,12161,12162,12163,12165,12166,12167,12168,12170,12171,12172,12176,12177,12179,12181,12182,12183,12185,12186,12187,12188,12189,12190,12191,12192,12193,12196,12201,12203,12206,12207,12208,12212,12214,12216,12217,12218,12219,12220,12221,12222,12223,12224,12225,12226,12227,12230,12231,12232,12233,12234,12235,12236,12237,12238,12239,12241,12243,12244,12247,12249,12250,12251,12252,12253,12254,12256,12257,12258,12260,12261,12262,12263,12264,12265,12267,12268,12270,12271,12272,12273,12274,12275,12276,12277,12279,12280,12281,12283,12285,12287,12290,12291,12292,12293,12294,12296,12297,12298,12299,12300,12301,12302,12303,12304,12305,12306,12307,12309,12310,12312,12313,12315,12316,12318,12321,12322,12323,12324,12326,12327,12328,12329,12331,12332,12333,12334,12335,12336,12337,12340,12341,12342,12344,12345,12346,12347,12348,12349,12350,12351,12352,12354,12355,12356,12358,12359,12360,12363,12364,12365,12369,12372,12374,12375,12377,12380,12381,12382,12385,12387,12388,12389,12390,12392,12394,12397,12398,12399,12401,12402,12408,12409,12410,12411,12414,12417,12419,12420,12421,12422,12425,12428,12429,12430,12431,12432,12433,12434,12436,12437,12443,12444,12445,12447,12450,12451,12453,12454,12459,12460,12462,12463,12464,12467,12468,12473,12475,12476,12477,12478,12479,12482,12483,12484,12488,12490,12491,12494,12497,12499,12501,12502,12504,12509,12511,12512,12513,12515,12519,12520,12522,12523,12524,12525,12527,12530,12531,12532,12533,12534,12535,12537,12539,12540,12544,12545,12547,12552,12554,12558,12559,12563,12564,12566,12567,12569,12570,12571,12572,12574,12576,12577,12579,12582,12583,12584,12585,12587,12590,12594,12595,12596,12597,12599,12605,12607,12609,12610,12611,12614,12617,12621,12622,12624,12626,12627,12628,12629,12630,12632,12634,12636,12637,12638,12639,12641,12642,12647,12649,12650,12651,12652,12653,12655,12656,12657,12658,12659,12660,12661,12665,12666,12668,12670,12674,12675,12676,12677,12678,12679,12680,12681,12682,12683,12684,12685,12686,12688,12689,12691,12693,12694,12697,12699,12701,12704,12706,12709,12710,12712,12715,12716,12717,12721,12724,12725,12727,12728,12732,12735,12736,12738,12745,12746,12748,12749,12751,12752,12753,12754,12759,12763,12765,12767,12769,12770,12774,12776,12777,12778,12779,12781,12782,12784,12788,12789,12791,12792,12793,12800,12801,12802,12803,12805,12806,12808,12809,12811,12812,12813,12814,12816,12818,12819,12820,12823,12826,12828,12829,12830,12831,12832,12833,12835,12836,12841,12843,12845,12846,12847,12850,12852,12853,12854,12856,12857,12861,12862,12863,12864,12865,12866,12869,12870,12872,12874,12876,12877,12879,12886,12887,12890,12891,12892,12893,12894,12895,12897,12900,12901,12903,12904,12907,12909,12910,12913,12914,12917,12921,12924,12925,12928,12929,12930,12932,12933,12934,12935,12938,12940,12941,12943,12944,12947,12949,12951,12952,12955,12957,12959,12960,12965,12966,12968,12971,12972,12975,12979,12980,12981,12983,12985,12986,12989,12991,12998,12999,13001,13002,13005,13008,13010,13012,13016,13017,13018,13022,13025,13026,13031,13032,13033,13035,13038,13043,13045,13046,13048,13049,13052,13053,13054,13056,13060,13062,13063,13065,13067,13073,13075,13076,13083,13084,13087,13088,13089,13090,13094,13099,13100,13101,13102,13103,13105,13108,13109,13112,13116,13118,13120,13123,13124,13125,13126,13127,13130,13131,13134,13137,13139,13141,13142,13143,13145,13146,13147,13153,13158,13159,13160,13161,13162,13163,13165,13172,13173,13174,13175,13179,13181,13184,13186,13191,13192,13195,13198,13201,13203,13204,13205,13209,13214,13216,13217,13218,13220,13221,13222,13223,13227,13233,13234,13237,13238,13240,13241,13242,13244,13246,13249,13250,13254,13260,13264,13267,13269,13270,13271,13273,13276,13277,13278,13281,13282,13283,13284,13285,13286,13291,13293,13296,13297,13302,13306,13309,13310,13317,13319,13320,13322,13323,13325,13331,13333,13334,13335,13337,13338,13339,13340,13341,13342,13343,13344,13350,13351,13357,13365,13369,13371,13375,13376,13378,13380,13382,13387,13390,13396,13397,13399,13402,13403,13404,13407,13410,13411,13413,13415,13420,13423,13424,13426,13431,13434,13435,13436,13437,13438,13439,13440,13445,13447,13448,13450,13451,13452,13455,13456,13457,13462,13464,13466,13468,13470,13476,13477,13479,13482,13483,13495,13498,13499,13500,13503,13505,13509,13510,13512,13514,13515,13519,13523,13524,13525,13541,13544,13546,13547,13548,13553,13554,13555,13560,13566,13567,13568,13572,13573,13577,13578,13579,13581,13582,13584,13585,13587,13593,13597,13601,13605,13611,13621,13623,13624,13627,13630,13637,13642,13646,13649,13651,13654,13659,13663,13665,13666,13673,13674,13675,13676,13681,13684,13689,13690,13691,13692,13700,13705,13712,13715,13721,13728,13729,13732,13735,13737,13741,13742,13744,13747,13753,13764,13766,13769,13770,13772,13776,13778,13780,13787,13790,13794,13795,13796,13797,13799,13800,13803,13806,13807,13813,13821,13823,13825,13828,13829,13830,13831,13836,13841,13843,13845,13846,13848,13854,13858,13859,13863,13871,13881,13892,13896,13902,13912,13913,13918,13932,13938,13940,13943,13944,13948,13950,13953,13955,13959,13968,13970,13971,13972,13977,13978,13985,13986,13988,13996,13998,14000,14003,14007,14010,14013,14019,14020,14024,14025,14028,14030,14037,14039,14046,14061,14066,14070,14082,14092,14098,14100,14101,14108,14109,14112,14120,14129,14134,14135,14141,14143,14155,14166,14169,14188,14190,14202,14223,14225,14227,14238,14249,14252,14253,14255,14267,14271,14279,14280,14289,14306,14308,14318,14345,14346,14347,14350,14353,14354,14355,14358,14371,14375,14382,14383,14384,14391,14396,14399,14412,14440,14442,14451,14454,14476,14477,14483,14490,14492,14495,14504,14521,14534,14536,14539,14540,14562,14566,14574,14583,14600,14622,14628,14631,14632,14640,14643,14647,14649,14657,14659,14661,14662,14669,14670,14680,14681,14682,14683,14685,14686,14699,14709,14715,14728,14731,14743,14784,14786,14807,14826,14830,14833,14837,14843,14847,14857,14859,14869,14885,14886,14903,14919,14944,14965,14972,14983,14986,14987,14990,14993,15001,15025,15028,15031,15044,15046,15047,15078,15089,15110,15120,15121,15125,15136,15172,15175,15191,15195,15211,15219,15221,15250,15260,15271,15284,15294,15306,15307,15309,15310,15331,15334,15344,15355,15359,15378,15381,15407,15412,15413,15424,15434,15438,15477,15481,15489,15492,15494,15495,15505,15517,15541,15544,15549,15559,15565,15577,15588,15601,15615,15619,15620,15629,15639,15645,15672,15674,15676,15682,15686,15690,15691,15700,15734,15752,15762,15820,15824,15825,15833,15842,15849,15852,15855,15862,15863,15918,15929,15954,15968,15983,16007,16015,16041,16042,16043,16057,16063,16066,16069,16073,16085,16090,16093,16096,16115,16118,16131,16155,16160,16168,16169,16170,16179,16184,16190,16231,16232,16237,16241,16249,16267,16321,16329,16372,16379,16398,16399,16416,16420,16423,16434,16438,16450,16467,16484,16512,16523,16533,16539,16551,16580,16583,16636,16640,16660,16673,16683,16684,16703,16712,16743,16748,16749,16755,16802,16824,16828,16829,16834,16836,16865,16867,16892,16911,16920,16940,16942,16945,16967,16971,16979,16980,16983,16990,17013,17028,17030,17042,17047,17060,17078,17101,17135,17147,17155,17180,17189,17218,17221,17231,17233,17243,17246,17277,17292,17308,17325,17338,17341,17350,17351,17365,17386,17397,17404,17415,17432,17480,17487,17503,17525,17558,17560,17566,17573,17611,17615,17617,17630,17712,17721,17733,17755,17758,17763,17767,17785,17790,17809,17839,17856,17859,17861,17872,17903,17909,17910,17913,17955,17996,18053,18056,18058,18076,18129,18135,18141,18160,18165,18199,18231,18246,18265,18298,18301,18310,18338,18360,18399,18414,18434,18443,18488,18511,18516,18553,18564,18595,18640,18665,18674,18696,18718,18750,18790,18791,18793,18814,18920,18994,19004,19014,19054,19067,19109,19125,19166,19268,19279,19298,19329,19336,19356,19365,19394,19421,19431,19483,19515,19524,19626,19641,19656,19660,19767,19791,19803,19836,19878,19908,20010,20055,20093,20134,20163,20178,20223,20231,20324,20358,20361,20386,20401,20412,20494,20658,20833,20854,20902,20997,21169,21185,21188,21193,21209,21214,21239,21256,21294,21377,21482,21654,21674,21708,21777,21826,21832,21859,22011,22035,22126,22143,22220,22240,22271,22324,22344,22364,22442,22454,22457,22473,22487,22537,22714,22754,22769,22803,22819,22878,22949,23011,23029,23061,23182,23206,23298,23333,23335,23359,23431,23446,23573,23825,23959,23960,24009,24088,24136,24294,24533,24580,24738,24788,24867,25032,25054,25059,25274,25288,25310,25361,25512,25717,25795,26546,26842,26967,27109,27224,27429,27484,27891,27898,28092,28263,29136,29421,29799,29992,30386,31208,31364,32308,33359,33442,34836,37135,39197,41022,43480,47957,49666,51970,53481,54892,59155,59753,71240,73899,88614,110884,117445,123555,124509 |
| Count | 150,133,154,145,140,111,145,123,126,143,147,139,139,132,135,117,124,130,132,129,137,127,114,116,130,147,124,112,129,132,123,128,146,121,105,142,140,123,124,118,102,109,128,129,113,121,117,105,124,124,119,124,106,97,108,101,104,122,108,118,120,116,108,109,100,116,106,100,108,101,114,107,106,96,113,108,108,93,93,113,105,98,93,97,88,105,94,97,100,99,103,97,101,102,96,75,106,89,78,80,100,97,100,88,87,97,94,119,102,103,86,90,85,108,107,82,94,87,96,89,90,79,94,96,78,90,98,101,76,96,112,106,96,87,85,60,92,89,89,91,79,80,85,96,98,88,89,88,81,83,82,86,88,94,81,76,94,69,104,73,79,93,91,90,111,90,66,68,98,82,81,79,77,78,94,76,82,72,81,94,80,93,100,87,83,73,90,87,101,70,85,93,77,75,78,63,85,91,77,83,80,78,79,76,87,84,83,87,92,86,81,86,67,76,94,73,88,68,76,88,90,82,62,95,81,83,66,86,67,69,80,64,75,91,82,88,89,88,71,71,73,54,81,87,92,72,86,71,85,83,81,76,84,96,63,78,67,73,80,77,66,76,72,80,57,63,77,72,78,71,89,79,79,89,65,64,81,80,82,71,73,81,69,62,78,84,71,75,76,69,72,69,71,89,82,90,73,84,77,76,72,74,74,77,73,79,66,69,69,76,73,76,79,69,84,75,75,53,77,76,81,75,76,83,80,82,86,67,67,81,76,68,80,71,70,82,90,84,77,62,78,79,93,66,73,88,68,63,77,88,88,67,80,77,70,58,77,61,78,68,77,73,71,85,68,96,66,88,62,65,67,88,69,71,75,70,63,89,80,72,67,77,70,78,77,68,77,82,70,75,79,59,72,66,59,74,63,71,86,75,61,61,76,78,77,70,51,78,71,59,59,69,73,77,71,82,75,62,99,64,83,67,71,57,82,88,72,60,71,80,65,75,83,77,96,71,74,63,69,78,72,65,67,73,83,62,86,66,89,74,59,66,75,69,71,65,73,76,79,84,76,73,83,74,76,84,72,71,65,67,68,75,76,78,71,73,89,66,65,79,59,73,73,62,86,69,90,68,68,70,90,71,91,75,67,95,76,72,68,80,62,95,68,90,77,76,84,73,89,64,56,82,75,74,77,84,80,78,80,79,78,68,60,67,62,61,82,71,84,91,76,70,74,88,80,91,84,92,77,72,79,78,68,79,53,89,67,83,68,75,74,84,80,75,102,88,70,73,75,78,72,92,85,75,87,69,87,89,88,76,88,67,70,90,87,71,89,84,68,74,65,80,96,82,77,83,90,78,81,71,86,76,75,73,86,79,103,92,68,98,82,83,79,75,70,86,90,69,78,85,72,92,78,97,83,90,64,84,85,94,85,84,85,69,87,94,85,92,80,86,76,104,58,93,89,92,83,87,94,83,86,71,81,80,80,109,96,89,104,90,79,100,80,81,79,74,91,83,82,76,102,80,71,98,91,81,80,90,83,89,80,87,86,84,90,80,83,87,99,77,82,93,95,90,79,81,101,89,91,92,89,75,83,86,78,90,92,72,96,88,91,74,76,84,79,106,80,92,93,92,90,78,86,73,90,89,92,90,102,83,86,91,83,88,93,91,83,93,90,85,94,83,99,92,93,95,94,88,95,83,110,87,89,101,85,111,95,80,76,83,86,97,82,84,108,86,83,87,107,85,84,101,109,81,95,82,83,85,94,97,69,87,84,93,90,110,92,93,87,84,99,101,95,82,95,93,88,99,95,98,86,98,87,103,97,105,97,91,69,98,90,91,78,91,111,85,97,102,98,84,101,104,102,91,93,92,97,92,95,87,86,100,74,97,101,96,76,85,98,87,87,95,88,96,99,97,85,98,91,89,96,100,101,89,88,94,87,95,104,98,102,86,111,100,103,82,97,86,94,97,99,107,87,93,95,108,113,88,92,110,105,71,99,91,114,96,96,76,93,103,97,87,104,101,84,95,117,88,76,108,103,100,91,97,94,105,101,93,95,107,104,88,98,106,80,98,95,81,82,96,89,87,80,88,101,90,95,108,105,111,100,76,105,95,100,94,107,115,113,93,113,102,103,95,102,112,89,98,129,97,105,92,92,86,93,109,104,116,103,94,99,84,110,93,131,110,112,114,120,102,99,110,100,112,105,99,106,116,104,117,112,106,118,103,104,120,135,118,122,120,90,97,101,92,108,137,98,107,108,116,111,101,106,108,123,93,117,107,101,130,96,93,132,119,121,107,91,104,120,107,121,115,95,106,115,105,112,102,101,109,96,124,105,117,119,104,109,106,117,122,95,104,107,105,109,125,116,95,121,103,111,123,98,114,123,122,112,107,109,114,104,118,113,118,143,99,88,112,117,119,115,132,133,108,117,146,120,119,103,129,105,120,102,114,136,112,118,106,105,125,116,117,129,104,88,123,117,121,113,113,127,111,117,99,112,107,141,133,133,124,116,131,125,128,105,112,120,110,127,122,99,128,118,127,101,107,132,123,107,152,120,113,134,111,108,144,109,134,125,122,121,111,122,140,136,131,116,129,121,120,120,123,144,134,116,112,127,138,123,121,133,104,144,141,139,128,143,120,121,111,121,122,109,131,128,127,135,133,120,138,120,128,137,122,144,126,126,133,125,129,117,114,141,123,118,108,141,134,119,131,139,135,118,122,135,122,117,153,135,130,125,114,139,128,135,121,135,134,129,113,129,123,135,134,130,152,123,132,135,123,120,126,136,155,125,137,149,132,140,111,142,135,113,131,130,133,127,133,143,141,126,111,145,132,143,138,131,123,114,151,151,134,140,146,146,127,122,138,135,125,137,122,121,116,145,136,146,140,143,133,126,149,131,125,135,119,125,141,142,141,130,120,135,150,140,131,128,119,146,132,121,121,152,127,138,135,146,118,130,121,152,120,156,151,142,134,133,134,134,142,127,147,134,131,141,140,144,135,123,142,141,130,138,125,87,128,154,142,109,131,118,140,125,129,119,131,119,121,135,155,122,131,125,133,124,136,139,117,152,138,133,129,144,111,118,123,125,135,143,116,123,115,124,117,139,142,138,117,118,126,127,127,131,130,136,138,117,111,116,129,149,120,126,127,138,141,118,126,151,130,119,129,143,120,148,122,132,127,132,111,145,133,122,119,123,116,135,132,109,127,118,135,121,125,111,117,109,110,125,114,126,129,112,123,104,112,113,121,119,104,107,112,101,107,131,113,131,113,122,120,128,115,108,137,131,104,105,118,126,110,112,130,129,118,106,131,122,131,128,125,109,121,115,115,104,107,107,98,132,107,113,111,109,119,114,106,116,91,100,103,126,103,123,105,107,102,121,83,81,124,104,124,93,101,90,109,100,110,110,110,99,115,106,101,115,117,104,101,97,108,90,119,114,104,123,105,113,105,123,110,115,111,114,107,111,113,90,104,103,106,105,112,84,108,98,110,128,107,108,95,103,111,98,107,101,113,104,96,95,97,101,99,112,92,100,100,104,149,94,106,84,94,91,97,99,95,107,87,93,76,114,103,111,96,87,97,103,88,91,78,96,67,80,106,86,93,97,114,104,100,90,89,87,97,88,87,102,92,116,88,93,97,100,104,86,80,92,92,95,95,82,96,94,89,88,89,90,86,76,67,97,84,94,84,91,73,97,76,75,96,90,74,89,72,95,88,64,78,90,71,81,71,89,76,81,99,83,90,53,90,93,80,76,84,82,94,84,78,78,102,82,71,86,64,75,85,92,72,86,81,105,85,67,89,79,83,89,63,79,80,68,88,86,60,67,79,72,73,86,72,85,62,70,81,65,75,65,75,79,71,69,81,80,72,70,70,68,72,78,73,69,60,68,70,72,48,83,59,65,70,73,68,60,70,71,59,70,62,69,74,62,55,75,73,87,69,74,68,71,67,74,70,64,64,72,56,77,69,63,62,83,53,67,52,77,71,75,61,69,61,71,45,66,66,55,79,58,76,74,73,62,52,52,63,54,63,65,70,63,49,64,55,65,60,57,60,63,58,54,71,70,66,62,70,55,83,61,55,63,80,62,65,51,78,45,53,47,52,55,54,50,60,56,47,59,62,58,58,42,61,65,57,60,60,61,71,72,52,60,65,46,49,55,59,56,57,69,60,49,51,76,62,53,58,52,58,53,54,59,50,56,55,73,55,50,57,48,52,53,50,50,51,58,57,56,61,60,42,48,65,53,40,68,60,60,49,51,53,57,64,61,49,68,58,57,49,46,69,50,51,52,68,54,53,60,55,58,49,67,39,49,48,52,63,51,50,53,51,70,61,61,58,58,55,67,47,54,51,65,58,44,46,45,54,59,50,45,53,49,52,36,58,46,55,49,45,46,39,49,40,46,67,53,50,66,63,64,50,47,52,50,47,61,50,54,52,40,53,59,33,50,54,50,48,45,56,53,51,42,43,45,56,52,55,50,45,50,44,51,70,54,58,41,54,49,51,47,45,66,62,43,50,50,55,59,44,47,56,42,65,52,56,53,48,52,56,71,45,51,60,71,45,41,46,66,47,55,60,53,64,57,49,51,46,54,68,40,53,49,45,63,44,41,79,46,49,56,54,74,58,49,56,60,50,49,46,57,50,56,59,45,42,53,46,55,62,74,63,59,53,57,51,48,71,57,49,58,44,63,54,54,57,56,55,45,51,66,62,56,48,46,48,47,47,51,52,55,61,59,46,55,58,56,53,46,70,47,62,59,62,55,56,47,42,48,53,47,45,50,56,52,59,42,57,59,66,55,57,56,56,66,53,57,56,53,53,56,39,55,37,46,55,59,54,57,51,48,54,51,54,57,48,61,53,62,59,44,51,45,64,48,51,51,57,60,52,42,71,46,72,55,43,59,43,47,62,57,49,59,42,71,67,61,67,58,68,48,55,56,42,60,43,63,50,59,41,45,48,50,49,60,53,51,52,50,73,59,55,54,75,39,58,49,45,47,60,53,59,55,49,67,52,59,53,60,54,42,56,66,53,71,55,61,69,59,57,69,47,47,72,70,42,50,58,69,65,58,60,59,50,49,53,51,62,56,54,51,52,51,55,63,55,59,54,58,55,58,44,62,57,48,67,63,57,63,62,52,54,47,37,52,54,43,44,47,57,69,49,66,61,58,62,57,62,59,48,46,59,59,51,53,56,62,63,57,52,62,57,62,50,55,51,64,59,58,50,45,64,63,59,61,46,53,49,65,44,52,41,55,56,62,63,70,57,79,68,55,58,55,62,50,64,66,63,62,70,60,62,46,56,59,72,59,55,54,59,56,64,40,51,60,65,62,51,53,63,59,79,59,54,73,57,53,73,64,58,60,53,55,60,54,60,56,53,68,53,61,51,65,58,75,58,71,60,49,60,53,80,61,50,57,62,55,56,55,53,51,61,62,62,53,63,58,59,53,56,62,60,55,61,57,58,62,62,66,66,54,61,56,55,64,44,64,56,54,64,44,52,47,56,55,71,64,58,50,49,55,66,61,56,68,55,58,68,61,70,59,48,56,47,82,56,49,68,51,49,61,59,46,52,65,54,54,64,69,60,61,63,60,58,59,52,64,61,53,48,66,55,53,61,48,68,52,53,62,55,55,50,44,54,62,52,58,63,66,60,55,63,64,47,83,63,57,68,59,52,50,57,39,60,51,53,56,54,58,59,56,80,57,60,71,55,59,59,68,71,50,63,59,66,65,63,62,66,66,68,69,59,54,59,57,54,57,59,73,57,58,60,56,72,62,61,57,63,50,57,57,51,57,50,42,64,53,68,59,48,58,54,51,65,51,49,60,66,58,64,68,62,50,62,51,59,48,61,36,45,55,48,60,46,55,54,61,54,60,57,58,56,57,63,54,57,61,57,60,56,58,51,50,59,42,64,53,57,54,51,57,60,65,63,49,49,65,56,59,65,53,49,63,62,66,50,64,56,57,54,56,48,52,56,44,47,52,47,67,50,59,65,59,60,61,61,49,55,46,62,59,56,51,56,42,53,44,64,44,61,59,61,56,54,54,61,50,49,57,59,59,55,64,70,49,60,59,49,45,56,48,47,50,64,54,53,57,53,56,58,62,50,53,44,39,42,55,60,68,58,42,42,60,47,62,65,65,52,53,59,61,55,51,56,45,54,44,59,46,68,56,71,58,59,59,55,44,55,48,39,54,74,49,40,48,61,44,49,49,55,53,60,56,53,52,39,43,56,45,54,52,57,65,60,43,57,54,42,64,57,56,53,40,49,43,51,54,80,51,61,52,54,48,61,58,53,60,53,60,63,53,43,45,48,41,54,50,59,51,54,61,58,52,56,50,59,46,50,54,54,55,48,47,62,66,48,56,51,53,42,50,49,44,56,46,41,69,50,49,42,52,44,42,47,45,56,60,58,53,47,40,48,48,61,60,58,59,63,54,54,47,43,47,67,47,49,45,56,48,52,49,48,50,44,77,49,51,51,55,55,50,55,44,57,48,48,45,44,60,48,58,52,48,64,49,61,48,47,49,52,56,50,45,47,53,49,46,52,59,45,34,53,59,38,51,59,53,59,35,51,60,45,60,53,35,65,46,53,53,41,58,45,53,56,52,62,43,53,46,63,47,41,55,45,45,44,51,48,50,45,51,49,39,46,58,42,47,42,46,45,56,53,44,48,42,35,40,55,49,43,62,56,48,36,43,44,42,45,49,42,56,39,52,57,54,47,43,40,45,35,52,52,45,40,49,57,47,43,55,42,53,46,41,34,38,50,46,56,41,56,46,46,39,45,41,56,47,35,41,46,45,50,38,46,36,41,41,43,47,56,41,45,45,50,46,45,41,33,35,39,42,41,43,54,39,46,49,48,53,45,52,35,40,48,43,42,48,46,36,46,37,47,51,39,49,42,46,29,56,33,50,43,43,48,46,38,53,45,42,50,52,49,42,42,53,51,39,51,50,42,37,37,53,51,54,46,48,42,49,39,43,52,41,41,47,47,44,66,51,45,40,38,43,44,45,34,42,47,47,40,31,48,53,44,42,37,50,36,40,37,36,41,41,46,40,41,43,39,37,53,44,43,51,38,33,58,48,35,41,44,45,46,30,46,46,46,41,35,47,41,50,46,47,37,55,49,40,41,50,39,50,41,42,37,46,36,38,40,40,34,38,39,43,37,45,42,49,45,41,30,48,36,42,45,42,42,41,34,40,43,38,52,36,34,45,45,47,47,47,41,41,43,39,40,45,41,39,26,41,41,32,42,38,45,61,38,34,48,48,35,42,42,47,44,39,38,42,37,29,28,53,40,44,38,46,43,46,39,51,61,31,33,50,38,22,36,28,47,46,43,29,21,41,34,41,37,43,41,36,44,32,37,29,39,45,32,30,38,33,44,47,29,44,30,38,40,38,51,46,49,42,41,43,50,31,40,47,34,31,37,39,44,44,40,47,56,50,43,33,38,43,32,37,33,42,33,35,41,40,44,51,48,39,39,44,37,41,35,39,39,38,35,46,40,44,55,43,54,46,51,40,37,34,46,41,34,47,31,38,42,37,39,39,33,39,42,37,45,44,35,35,40,35,43,28,47,40,45,47,48,39,51,43,30,35,33,45,38,30,31,49,39,38,37,39,43,35,45,33,31,27,23,45,34,47,35,41,44,53,45,34,48,49,49,40,39,40,40,39,36,43,31,44,25,38,43,49,42,46,38,32,35,31,39,43,27,30,43,29,32,40,28,44,39,54,43,40,34,40,40,30,40,43,35,41,39,41,37,55,30,27,34,33,36,30,36,43,42,43,31,33,50,31,31,28,36,35,39,35,43,41,33,43,45,48,25,30,30,42,31,38,38,37,35,38,36,27,44,46,27,29,36,38,43,37,42,36,36,32,34,28,33,40,38,37,51,41,33,49,30,30,42,33,33,43,38,49,46,22,35,36,28,36,52,40,36,40,34,40,37,41,34,42,38,44,41,35,41,37,45,40,40,18,36,32,35,38,30,29,38,41,33,52,36,46,42,36,48,37,39,32,37,37,43,29,39,35,51,28,34,36,40,43,28,47,38,47,32,30,37,26,35,39,25,45,41,36,37,23,39,26,36,36,43,48,37,43,36,41,35,33,28,45,28,41,44,46,37,44,29,37,31,30,53,39,31,32,34,36,33,26,34,22,37,33,38,28,30,35,42,38,33,39,37,36,35,29,35,31,46,30,34,38,41,38,36,36,40,29,39,33,31,39,40,38,39,38,43,24,37,38,33,35,46,37,37,43,34,27,36,36,31,37,22,34,45,34,43,34,32,38,34,25,35,39,33,33,33,39,44,26,31,35,45,32,26,35,37,36,39,49,38,44,34,36,37,41,32,36,37,34,26,43,34,27,40,32,25,38,40,33,43,30,28,40,29,35,45,41,36,30,33,36,37,24,34,37,35,42,30,51,46,34,32,42,30,29,39,43,23,34,33,37,39,43,34,35,32,47,30,27,41,31,35,41,42,33,40,35,34,37,31,38,28,34,33,36,40,30,35,37,31,37,31,38,37,39,35,35,34,46,24,32,35,40,42,33,35,34,33,28,39,47,26,29,37,28,38,29,32,30,36,22,39,41,45,31,32,32,38,27,31,34,40,44,35,42,45,35,50,41,31,31,34,34,27,32,38,41,38,34,33,33,35,26,33,31,38,38,39,39,31,38,36,37,38,36,32,38,37,37,29,28,29,36,22,37,35,42,29,21,36,31,30,29,46,39,43,49,39,35,36,40,21,37,37,40,40,34,31,38,40,36,46,45,34,36,37,39,45,45,41,39,37,37,25,36,37,35,32,38,32,35,39,39,32,41,23,43,31,29,33,35,35,30,40,34,34,33,27,31,27,37,41,39,33,38,34,38,29,15,25,33,40,32,46,40,31,35,34,33,36,25,26,42,49,36,43,31,39,27,44,28,27,34,30,25,42,29,29,29,39,38,45,33,33,45,31,33,42,43,28,33,38,24,38,39,27,30,31,26,28,39,34,29,31,31,38,46,30,18,29,31,27,29,32,27,31,36,42,40,29,28,33,40,32,30,39,32,50,21,29,30,34,29,25,31,29,31,37,30,42,26,40,33,27,29,34,22,38,27,34,23,30,37,32,41,29,34,37,36,33,37,28,21,34,33,45,29,29,26,29,26,40,27,22,37,33,28,31,42,25,30,29,31,38,35,34,33,33,34,28,27,27,32,30,32,32,44,36,21,38,22,32,28,26,36,27,31,36,34,40,34,33,36,25,33,32,38,44,32,26,25,34,36,33,37,44,28,38,30,30,31,32,34,40,33,31,35,35,31,26,30,32,27,38,27,29,22,37,34,39,25,26,33,28,42,30,47,32,27,41,36,22,37,22,42,34,33,34,39,30,25,30,36,28,33,46,25,24,27,26,31,24,24,34,32,26,32,34,32,32,29,34,37,40,47,34,25,22,29,24,31,22,31,24,31,39,24,25,38,29,30,40,34,39,24,29,32,29,25,32,24,28,31,30,26,25,23,26,37,37,30,23,32,30,33,27,39,41,33,29,26,35,26,25,37,38,30,24,31,29,22,35,24,32,30,32,33,28,42,32,33,27,33,31,39,28,27,23,32,23,33,19,33,26,31,35,27,25,31,31,48,24,26,24,26,26,33,30,26,28,28,31,23,33,36,27,26,25,34,30,42,22,29,30,35,23,30,26,39,34,27,30,25,23,32,16,35,37,32,25,28,26,26,38,41,26,38,23,23,29,41,32,39,32,36,30,27,32,33,34,32,31,34,34,27,30,38,22,37,31,28,16,25,25,24,16,28,20,38,38,35,35,31,29,33,28,26,32,28,24,27,28,22,36,25,25,31,23,29,27,34,27,40,30,32,30,25,25,22,23,24,31,40,24,36,28,30,18,29,34,32,25,23,28,32,24,25,33,37,27,27,22,20,34,33,30,25,23,23,34,20,24,27,23,28,30,33,27,29,15,23,40,20,26,31,37,29,34,33,31,36,35,34,38,22,30,38,39,33,24,28,36,29,39,33,38,37,23,30,20,30,34,24,27,27,29,21,25,36,34,28,24,44,26,21,35,26,33,40,24,33,32,29,29,19,28,31,33,27,24,27,36,34,39,30,19,12,25,29,22,30,24,21,28,23,28,27,30,21,26,27,35,37,30,24,28,28,20,31,30,29,36,33,31,26,30,30,31,28,33,24,27,29,24,20,35,18,28,27,29,28,22,33,29,25,18,31,28,22,24,42,26,23,22,22,30,22,34,25,20,23,18,16,29,33,26,25,25,22,36,20,31,30,22,39,34,36,25,28,26,32,25,31,28,18,37,26,26,35,27,25,33,22,32,25,18,27,23,32,28,26,19,24,35,24,30,37,32,23,22,20,22,28,23,29,36,34,34,34,38,27,20,28,21,30,30,32,31,19,21,13,25,23,25,30,22,30,34,25,16,40,29,32,29,28,24,23,30,33,32,21,23,21,28,21,21,19,29,19,23,25,23,30,36,30,24,39,28,30,34,24,32,28,34,26,24,18,23,26,27,25,32,31,22,24,35,31,35,29,27,36,24,23,24,26,25,28,22,21,31,33,30,26,25,38,39,26,28,20,34,23,27,26,32,32,16,26,26,27,26,23,24,26,22,26,29,25,21,28,27,26,21,27,23,25,22,33,29,25,38,21,24,35,24,24,31,20,24,23,24,30,23,26,19,21,30,32,19,38,35,17,21,25,25,27,22,26,20,31,32,26,31,18,25,30,24,20,28,33,25,27,23,18,31,32,36,27,26,26,27,19,21,22,32,30,20,25,34,25,21,27,33,28,29,29,37,23,24,36,28,22,27,29,21,30,25,20,31,22,27,29,27,19,26,32,28,37,23,23,22,15,29,30,32,20,22,26,22,29,22,24,32,32,23,29,18,25,21,29,22,28,27,26,27,29,31,26,27,24,21,25,20,15,19,21,36,29,19,24,18,42,26,22,33,26,28,32,30,27,26,33,29,24,32,23,20,33,33,29,31,17,33,30,34,35,21,25,30,33,31,27,31,27,22,27,27,19,22,30,23,21,29,21,26,19,27,29,21,23,27,21,28,40,35,31,29,34,33,28,28,23,23,33,24,22,27,22,29,25,23,19,21,18,26,27,29,25,26,34,33,23,31,28,28,25,28,28,37,30,24,25,25,30,26,16,22,19,20,30,29,23,23,21,20,24,22,23,20,26,27,31,24,31,24,30,21,22,25,17,23,19,23,22,22,20,36,30,25,34,32,29,32,37,25,19,34,28,27,31,25,23,31,24,31,22,18,21,32,24,28,37,28,35,28,16,34,26,31,21,16,38,24,28,22,26,34,29,17,23,21,29,16,29,23,29,19,31,18,29,21,29,22,29,30,36,26,19,28,23,25,30,22,25,26,26,19,26,31,25,32,21,29,28,30,28,22,21,25,21,26,28,25,29,16,21,25,27,29,29,27,28,24,26,30,20,32,34,35,26,28,21,24,23,21,23,19,29,31,26,26,33,26,22,22,19,21,35,26,22,24,27,23,25,26,28,23,16,24,21,22,28,20,27,17,21,21,25,27,22,28,25,25,17,16,27,28,27,25,25,26,30,32,32,18,26,19,31,20,26,18,32,23,24,25,26,15,40,22,23,36,30,21,30,32,31,28,20,30,27,29,19,27,18,19,26,27,23,23,31,19,15,33,21,17,33,29,25,27,30,30,21,26,24,30,24,24,21,19,20,32,18,31,27,20,33,27,18,20,20,24,23,31,20,29,27,26,27,31,30,27,36,29,37,32,23,30,33,23,25,28,21,20,29,23,14,21,31,33,25,27,25,27,24,22,22,34,24,27,25,21,33,27,36,24,19,19,33,29,25,22,18,25,26,28,16,25,22,19,20,33,22,21,23,24,16,19,29,31,22,23,21,20,9,42,32,22,21,22,29,27,26,24,22,24,23,22,15,34,23,23,21,15,16,20,24,27,13,18,25,23,26,29,37,22,19,26,21,24,19,21,22,30,25,22,13,28,25,24,31,22,26,22,13,22,16,22,23,21,21,19,23,27,28,23,25,25,23,21,28,20,21,14,22,24,23,22,17,30,25,23,28,20,34,25,22,25,25,27,26,27,25,23,16,25,27,26,22,23,16,22,31,28,27,16,20,27,19,28,23,20,24,31,26,17,20,24,16,19,27,21,18,29,25,26,20,16,29,23,30,21,22,27,24,31,12,24,26,32,25,19,24,19,27,27,19,28,21,15,20,21,22,29,30,22,27,21,32,9,22,23,23,22,28,20,28,26,32,22,32,26,30,28,24,14,30,19,32,26,21,23,30,28,18,22,19,19,21,28,34,28,24,21,27,29,25,19,22,27,15,22,24,17,24,26,19,23,28,24,25,25,27,22,21,33,32,26,21,31,23,28,30,27,24,31,24,15,37,20,24,24,20,28,23,33,20,23,22,14,28,26,17,25,18,19,26,21,25,27,22,20,25,22,17,21,22,20,19,15,13,27,15,27,27,27,27,23,19,22,21,26,28,25,21,22,26,22,24,24,31,18,19,24,18,29,20,28,20,16,27,17,21,19,31,28,24,16,21,17,20,28,29,26,25,24,21,30,17,24,27,18,27,15,17,29,30,19,28,27,22,23,11,23,23,17,26,21,32,22,25,30,26,19,38,29,15,23,14,26,25,25,28,32,24,22,15,25,18,29,23,29,23,25,30,22,25,28,20,27,27,26,16,25,23,19,21,24,38,28,31,21,30,23,23,30,21,31,21,16,20,20,23,25,23,17,24,28,22,25,17,21,16,33,17,36,19,22,22,23,29,20,17,17,29,21,25,21,28,28,24,24,22,27,27,22,34,16,20,21,16,26,26,12,20,17,30,10,23,23,28,26,14,22,20,20,24,24,18,21,19,18,18,17,26,15,19,29,22,23,20,17,26,22,21,19,21,30,20,20,30,21,27,23,27,25,24,29,31,23,16,11,25,18,18,27,27,37,16,21,19,26,9,28,28,26,33,16,22,32,11,25,31,16,18,24,18,21,28,22,21,22,19,21,24,23,21,21,21,23,17,21,21,23,18,27,22,22,26,21,25,19,19,23,29,18,21,26,26,24,24,17,21,32,26,25,17,31,20,23,25,30,21,29,21,19,24,23,33,19,27,26,21,19,17,20,20,18,22,27,25,23,17,30,18,23,26,30,15,18,30,27,23,26,24,21,30,27,24,26,19,27,19,23,23,23,22,18,27,17,19,21,19,27,22,30,18,17,16,24,19,19,13,17,25,22,27,19,24,18,22,22,23,18,22,32,25,11,20,13,16,35,14,19,28,14,25,18,15,21,18,16,24,23,23,21,27,30,19,26,24,18,20,21,23,28,17,27,19,29,20,20,18,27,14,16,27,26,16,22,26,18,14,24,17,25,23,15,20,24,20,24,20,23,19,24,22,21,19,37,26,24,25,16,19,35,36,13,23,24,24,22,24,28,16,18,20,28,18,24,19,18,23,27,30,18,18,23,29,24,27,23,22,23,23,24,24,20,25,19,16,28,26,12,25,14,18,24,20,23,25,21,20,17,29,27,18,21,24,21,27,22,21,28,29,29,28,28,25,31,29,24,20,17,25,18,18,27,10,25,23,27,21,18,18,18,27,20,27,22,24,23,11,19,30,22,17,25,19,20,19,22,22,29,8,16,33,25,21,17,20,19,20,21,30,15,16,24,27,22,26,16,25,20,28,25,27,23,14,19,24,28,18,24,20,20,21,24,20,26,22,30,23,19,23,25,18,28,28,20,22,13,18,17,23,20,22,21,20,18,16,22,20,31,23,25,16,11,17,16,19,25,17,26,24,14,23,19,27,15,22,21,25,20,24,23,17,18,27,24,18,18,22,21,20,16,23,22,16,25,19,23,18,22,21,21,28,18,21,28,16,20,19,25,24,19,20,18,16,20,20,24,15,22,21,29,15,21,23,24,27,18,22,22,20,26,33,24,25,29,21,21,26,15,25,27,20,22,25,27,22,21,15,18,13,27,21,25,18,26,23,18,24,24,30,20,24,22,21,19,15,13,19,25,15,26,26,24,23,17,23,13,18,22,26,22,19,25,22,14,21,23,31,30,17,18,19,32,18,24,22,22,23,26,26,8,27,16,23,19,20,20,22,23,19,28,16,14,23,20,28,13,24,15,16,30,14,23,23,14,23,23,15,19,22,22,19,19,19,24,17,17,20,23,14,26,20,22,24,25,20,25,19,22,19,29,26,21,22,20,17,23,22,21,14,18,18,22,23,28,27,18,22,21,23,24,23,19,20,23,21,20,19,24,24,21,28,20,26,12,21,17,20,24,29,18,20,31,22,20,25,25,33,19,24,29,26,21,19,20,21,13,21,33,25,26,19,23,23,19,23,24,27,19,17,23,26,26,23,22,30,23,29,20,22,18,20,25,25,17,27,23,16,21,22,20,20,22,24,26,23,19,16,23,23,19,21,27,20,25,18,15,28,23,14,19,22,18,16,24,19,28,22,15,23,17,15,21,19,29,20,20,25,15,19,21,22,20,25,21,21,21,17,16,20,26,27,13,24,21,24,19,20,18,25,18,18,20,30,30,22,18,24,25,17,23,24,19,27,23,25,27,35,17,16,21,21,24,22,18,22,18,20,22,25,27,24,27,18,22,23,19,23,21,17,27,21,20,18,26,21,27,24,13,26,17,21,23,28,23,22,24,19,25,10,12,26,14,24,18,17,9,19,16,23,27,18,23,18,24,18,11,18,28,18,24,23,29,20,16,26,25,19,22,14,27,17,11,26,23,21,20,25,23,16,18,15,28,20,21,14,20,19,26,22,17,19,23,25,21,19,20,26,27,17,16,26,23,12,20,21,21,25,25,20,18,21,19,25,28,32,13,18,19,24,22,19,13,20,23,22,20,20,21,15,18,20,19,26,13,27,24,27,20,20,28,23,17,30,28,19,35,24,21,19,27,19,22,24,29,21,19,24,19,20,22,16,23,28,19,24,18,19,23,23,25,32,19,20,28,18,15,26,22,24,23,16,22,21,20,26,19,19,24,10,29,27,23,19,29,24,19,20,23,19,26,24,20,21,27,22,28,19,17,25,27,16,22,34,14,24,34,22,22,32,24,23,20,14,24,19,15,28,20,21,31,22,14,25,27,18,20,25,20,23,26,23,22,21,32,22,19,23,27,20,16,17,27,21,31,19,15,24,22,27,26,30,17,17,26,27,21,27,14,24,12,15,19,21,17,13,20,18,29,35,17,31,20,18,24,28,20,16,13,21,19,19,28,24,27,15,19,20,29,20,31,19,22,22,27,24,24,22,18,18,16,33,15,17,15,16,14,24,19,17,23,8,25,25,19,23,17,23,22,22,20,23,20,23,27,31,22,13,26,20,22,16,16,32,19,17,15,14,23,18,25,30,26,26,35,30,18,22,20,28,25,20,21,23,18,28,21,26,25,15,22,25,26,18,28,20,17,16,27,17,19,29,15,20,27,25,27,14,25,23,23,31,24,19,28,20,34,17,23,28,18,16,22,17,21,28,16,28,17,18,13,20,17,26,17,20,19,23,24,20,26,21,19,24,21,15,18,15,17,18,20,31,21,19,24,21,19,28,23,26,22,23,22,31,28,25,21,18,22,21,19,20,24,21,24,22,22,22,16,17,20,28,25,29,23,21,19,24,21,34,10,23,24,15,22,26,30,23,30,19,25,19,15,11,22,18,16,22,23,24,14,19,23,28,22,16,22,24,31,22,22,22,18,19,18,27,22,10,30,25,21,17,15,15,15,16,20,20,22,19,29,40,17,22,19,17,27,23,24,24,19,31,19,27,21,16,25,27,24,21,19,25,21,16,17,20,21,28,30,18,21,24,24,22,16,21,20,20,22,16,21,14,17,24,12,29,23,22,23,20,20,27,23,18,23,12,24,16,25,23,20,23,18,19,17,14,18,18,14,23,22,34,15,26,23,18,30,22,19,19,23,26,23,26,21,32,22,19,21,26,27,19,31,20,20,19,24,18,24,14,30,25,21,28,27,20,17,20,19,26,17,23,25,24,20,21,19,25,20,22,21,21,20,21,15,17,24,16,32,25,20,20,26,26,24,25,18,27,28,19,24,20,24,24,18,22,17,16,29,25,27,18,17,27,18,23,27,21,15,31,17,18,16,21,20,25,9,26,14,16,23,26,22,27,17,19,12,27,22,22,23,25,25,17,13,21,18,14,25,21,27,22,16,17,18,28,25,26,22,20,24,19,23,22,12,24,15,26,18,20,13,13,24,17,20,19,19,19,23,21,28,11,18,19,17,16,29,23,24,23,13,26,32,20,30,18,20,26,17,32,19,22,16,20,24,18,16,19,26,25,14,23,15,18,25,21,24,22,28,24,19,13,19,25,16,20,17,13,13,27,14,19,30,22,18,22,22,22,18,20,17,20,27,23,21,24,24,22,21,23,24,22,12,21,17,22,23,22,17,27,14,23,21,30,19,25,20,30,32,26,17,16,25,29,23,15,17,27,41,20,28,14,15,20,19,28,17,18,30,22,24,22,24,23,22,16,19,20,23,26,20,25,17,17,21,16,25,19,24,25,25,21,19,21,26,17,24,16,28,20,22,16,26,16,14,20,20,21,22,23,20,21,27,26,22,36,22,25,24,26,14,17,25,29,32,27,13,18,19,22,19,16,23,14,20,20,24,31,17,24,21,29,24,22,20,24,12,14,18,18,15,26,24,20,32,23,27,18,20,18,17,18,25,23,14,21,24,16,23,21,18,21,22,20,23,22,16,20,14,22,16,17,24,19,17,23,25,17,25,31,28,24,22,21,19,19,19,17,20,21,26,27,12,25,19,15,25,23,27,19,23,20,25,18,21,20,18,18,27,19,36,19,20,23,21,21,22,18,28,13,18,22,26,17,30,22,23,20,28,17,22,26,18,23,21,12,15,21,23,20,22,14,27,24,20,17,22,18,15,19,25,17,24,19,20,26,22,24,25,20,20,18,26,21,29,21,15,19,20,15,21,26,20,22,18,23,25,26,23,16,24,21,19,15,24,22,22,16,16,23,20,20,19,23,18,17,16,19,34,20,24,21,23,24,18,13,20,21,12,20,13,29,23,25,21,22,16,18,21,23,19,16,23,15,25,30,27,17,23,20,21,17,31,19,21,12,18,14,12,21,21,27,24,24,18,19,16,19,22,22,21,21,21,16,26,28,20,16,13,17,19,11,25,25,22,20,20,23,22,19,14,23,15,23,16,18,18,23,23,19,27,16,24,19,26,20,26,23,21,23,18,18,20,21,17,23,16,15,25,17,16,23,20,24,18,17,23,18,13,18,18,24,16,19,13,20,22,19,26,16,15,18,13,19,28,12,22,19,24,18,16,14,21,14,15,28,22,33,20,18,16,25,22,20,19,14,19,27,9,13,16,26,18,21,16,18,20,16,21,21,19,19,21,27,19,23,18,21,16,22,20,16,23,19,26,22,28,21,14,23,25,18,15,19,15,21,22,22,15,13,20,13,23,26,21,23,16,14,22,21,17,19,20,17,22,12,13,19,18,22,19,15,17,21,29,19,18,23,23,25,21,20,24,14,19,23,16,25,18,17,15,20,14,10,20,16,15,14,22,22,25,10,9,29,25,14,14,19,19,18,16,12,18,23,20,23,14,16,25,22,13,17,16,21,13,13,17,23,24,21,17,10,21,19,17,20,23,18,23,20,13,22,17,15,24,25,21,19,22,31,15,25,21,26,27,22,25,17,19,19,13,21,15,21,20,17,24,21,20,22,15,22,16,20,18,17,23,17,22,13,25,19,22,14,18,15,24,25,18,24,25,19,14,14,15,20,10,32,24,15,24,21,13,19,13,20,16,19,19,19,21,19,22,17,15,27,17,16,19,23,15,18,16,19,18,19,14,14,18,19,13,26,19,19,24,25,17,16,14,19,14,14,19,19,29,20,19,17,16,27,14,12,18,16,20,21,10,19,20,19,20,19,20,21,18,23,20,11,19,16,16,13,23,14,18,20,10,15,22,9,18,10,13,16,19,20,13,13,15,18,25,13,24,24,18,24,22,18,21,14,11,11,23,19,16,17,20,22,10,17,16,22,21,20,23,21,13,30,14,24,11,21,15,21,17,18,14,22,15,17,12,13,18,11,8,18,17,19,14,13,10,19,16,12,19,18,21,27,23,19,21,15,21,17,15,17,19,15,18,13,19,15,12,20,22,11,21,19,12,15,14,13,19,13,20,18,17,17,25,21,21,21,18,18,21,16,14,12,12,17,17,18,14,19,16,15,11,22,16,21,12,16,15,16,14,18,21,12,20,20,15,13,16,26,17,14,21,17,16,17,20,15,14,18,22,18,21,13,18,11,23,25,20,14,24,10,31,16,18,19,24,20,15,24,13,20,16,24,15,22,18,12,15,11,12,20,15,23,24,19,14,16,9,14,18,24,24,15,7,11,11,22,23,17,12,16,19,15,14,14,16,13,19,17,16,17,14,16,21,17,13,21,19,16,12,15,9,19,10,23,15,19,18,16,19,16,13,14,21,12,15,13,21,21,13,22,18,13,16,16,16,21,21,16,16,15,18,17,11,22,19,7,17,17,19,17,10,15,6,15,18,13,14,24,15,13,20,15,12,12,19,11,12,15,12,17,19,13,23,23,18,14,16,19,17,17,11,16,17,18,17,23,12,14,15,19,16,17,17,18,16,8,17,16,9,17,17,15,16,13,12,18,17,19,10,14,16,16,16,12,12,12,10,16,5,16,6,10,12,17,19,10,15,16,20,18,20,14,13,14,23,27,13,17,17,13,11,13,14,20,13,20,15,14,14,24,13,12,12,16,13,15,15,19,15,16,11,14,15,11,16,15,12,12,10,14,21,12,12,10,9,8,14,18,16,13,11,21,12,12,13,24,9,10,21,16,9,10,15,6,21,15,13,8,20,11,14,10,11,16,14,11,16,16,11,13,9,10,11,12,17,21,10,12,21,17,15,17,14,14,14,14,13,19,16,10,14,8,10,16,14,11,17,7,13,11,14,15,19,15,15,20,13,18,15,15,12,16,19,19,9,17,17,16,11,16,9,17,9,14,15,18,7,7,14,18,15,13,13,21,14,15,16,10,15,11,11,11,12,12,12,13,12,10,12,15,18,23,12,13,12,17,8,11,14,13,17,18,10,10,14,12,12,12,12,9,22,13,18,15,17,11,10,7,9,12,9,16,18,9,12,13,15,12,14,12,4,6,13,14,11,13,12,9,12,11,16,14,9,5,11,19,24,10,15,9,5,12,18,15,11,16,13,11,16,17,8,18,9,12,21,9,11,16,9,17,17,8,13,9,15,13,14,9,6,10,10,15,17,15,11,10,8,11,4,14,9,16,25,14,17,11,6,13,17,10,16,6,12,10,16,18,7,15,6,8,15,10,8,16,17,9,11,15,10,14,14,9,13,12,9,13,14,12,14,16,9,11,9,17,12,14,19,10,10,11,16,12,8,14,8,7,14,12,9,9,15,10,14,12,13,10,13,4,12,8,15,8,10,14,11,17,11,17,11,15,15,14,7,11,17,9,10,14,12,12,7,9,14,13,12,11,11,5,13,8,8,14,10,10,12,9,19,13,5,14,11,12,9,8,10,9,16,12,15,10,8,9,11,14,9,4,10,13,13,10,11,8,13,14,6,13,7,8,11,19,11,15,7,5,11,17,9,9,12,9,12,9,15,9,11,16,11,11,11,9,11,9,12,9,8,9,9,9,16,20,10,11,8,7,9,11,12,10,14,13,17,10,22,7,5,17,9,8,9,8,20,8,6,13,7,9,11,7,5,10,7,9,19,6,8,8,10,9,6,9,5,7,9,8,11,7,12,5,14,9,9,13,14,12,7,8,14,8,9,8,11,6,10,7,14,15,6,11,14,11,8,7,9,8,5,12,9,17,8,10,9,13,16,8,6,12,6,12,9,5,5,8,15,12,11,12,9,10,11,12,18,10,10,9,8,10,8,10,13,5,10,10,7,17,11,10,9,9,10,10,6,10,5,10,14,6,6,12,7,10,7,13,12,6,8,10,11,8,11,6,8,9,7,8,8,12,7,12,9,12,6,14,10,13,8,6,10,10,3,7,7,8,9,12,13,5,11,8,11,11,11,14,8,14,6,8,7,13,8,6,14,6,10,11,12,9,6,10,8,7,9,11,8,7,10,10,8,15,8,5,11,11,10,5,12,11,8,5,7,6,11,6,7,11,8,13,6,14,9,10,8,5,7,17,10,9,12,6,9,10,11,9,11,8,6,8,13,7,11,7,7,13,8,9,7,5,7,10,9,9,4,6,9,10,5,9,7,8,10,10,5,6,9,5,9,9,15,12,2,16,7,6,12,4,7,9,10,7,6,11,4,9,6,9,8,2,7,12,8,4,8,5,14,6,13,13,9,10,15,7,6,9,4,11,4,10,9,9,4,8,5,5,12,6,5,6,10,10,8,7,9,5,7,11,7,6,4,9,5,8,14,9,10,9,4,9,8,8,8,8,6,11,5,4,5,7,10,6,5,8,6,7,4,5,6,14,9,11,11,6,11,7,6,6,1,4,7,7,7,11,6,5,3,8,9,5,7,9,5,10,7,7,7,6,8,7,10,6,7,4,7,4,3,4,5,6,5,4,6,8,10,7,6,6,9,5,8,9,9,7,5,9,9,3,7,11,9,4,10,6,9,9,8,2,4,6,9,7,9,7,5,6,9,6,3,6,10,4,10,8,8,5,5,7,5,9,6,5,9,7,7,4,13,8,3,8,6,6,10,4,5,13,6,1,3,9,4,5,3,10,7,7,6,10,9,4,2,8,6,8,9,7,6,7,5,6,6,7,11,6,7,4,6,10,4,8,2,4,4,3,2,6,7,8,3,9,7,11,8,7,8,9,8,6,5,5,6,4,4,7,7,8,9,8,5,7,6,7,8,5,4,4,8,9,5,6,10,4,4,7,1,5,7,6,3,2,8,6,4,7,4,8,7,9,7,9,10,4,3,1,6,9,4,10,9,5,7,12,5,5,4,7,5,5,5,5,8,6,11,7,5,4,10,5,6,6,5,5,7,6,5,3,3,4,4,7,4,4,6,5,8,5,5,9,5,9,8,5,3,6,4,5,4,4,4,9,6,7,3,4,6,6,4,5,3,6,6,4,5,2,5,5,7,3,4,5,5,11,6,2,4,6,3,6,9,2,10,7,6,7,5,4,9,5,5,9,9,1,8,5,6,6,2,6,6,4,6,4,4,3,9,4,4,9,7,3,2,8,8,3,1,6,4,2,1,5,8,11,7,2,6,5,4,3,6,5,4,6,5,5,7,3,6,3,4,4,10,9,3,5,9,6,2,2,6,1,6,5,7,5,7,5,8,5,6,2,7,4,5,4,4,9,6,2,6,7,8,5,9,4,4,7,4,9,5,10,7,2,6,5,3,8,4,7,4,3,9,4,7,3,5,4,8,6,4,8,7,5,3,4,5,6,4,5,4,4,7,8,6,8,3,6,5,7,6,3,2,12,8,5,2,11,4,5,7,5,4,7,7,3,1,3,6,6,4,6,4,5,5,2,6,4,7,5,3,3,3,4,3,3,2,4,4,4,7,7,10,4,3,5,4,2,5,4,6,3,5,3,6,5,4,7,3,1,4,2,5,5,4,3,5,6,6,6,6,4,5,8,8,4,5,6,6,7,3,5,7,5,9,6,7,4,3,1,5,7,5,5,7,3,7,5,1,5,3,4,3,3,7,6,8,7,3,2,4,4,5,4,1,4,5,5,5,4,9,2,7,7,4,2,7,6,6,4,5,3,8,4,8,6,3,6,5,1,6,2,4,9,5,5,3,10,3,6,6,6,4,5,2,5,2,8,7,5,5,5,6,4,6,7,6,4,7,9,4,5,3,6,3,3,5,3,3,2,4,2,4,7,7,4,7,1,1,5,6,1,2,3,3,4,5,5,4,5,7,2,4,4,4,4,8,1,5,6,2,4,4,8,3,1,3,5,2,3,5,10,3,7,3,6,4,4,1,4,3,8,2,1,2,7,10,6,3,4,4,2,7,1,4,5,1,2,4,4,3,4,4,4,7,6,3,5,7,5,2,2,6,4,5,3,1,5,4,5,4,8,7,4,3,5,1,3,3,5,4,5,1,2,4,4,2,1,5,5,3,6,2,9,4,4,3,4,3,1,3,6,4,9,2,3,5,4,4,5,4,5,4,4,5,5,3,4,3,4,4,2,6,6,3,3,3,2,3,4,4,2,7,1,5,3,5,4,4,3,2,7,5,3,5,2,7,3,3,5,2,1,3,3,6,2,4,4,3,7,7,5,3,3,1,6,6,3,4,8,1,4,5,4,2,2,3,6,5,2,3,4,2,2,4,6,5,5,12,3,3,3,4,2,5,5,5,8,1,4,3,4,3,2,6,3,2,3,5,1,7,5,5,1,4,3,1,4,5,3,2,3,3,8,3,5,1,3,1,2,9,4,3,4,3,1,3,6,4,3,3,2,4,8,2,2,2,4,3,5,4,6,4,5,1,2,1,7,4,4,6,2,3,4,4,5,7,1,7,6,4,1,3,4,1,8,3,2,1,3,5,6,4,2,4,4,3,2,2,5,5,3,6,4,4,4,2,2,6,3,5,4,3,5,4,4,1,4,3,7,3,6,5,5,1,1,3,2,2,1,6,2,2,2,7,4,2,2,7,5,4,1,3,7,4,2,4,6,4,5,1,1,5,2,3,3,2,7,5,9,6,3,7,5,3,4,2,1,6,5,6,3,4,3,1,5,4,4,4,8,3,8,5,4,1,1,5,7,2,4,5,3,6,2,4,4,4,3,9,3,3,2,3,1,3,3,2,1,1,1,5,6,6,3,4,3,2,3,3,3,2,6,4,2,3,4,4,2,2,4,3,8,4,5,3,4,2,4,3,1,2,4,3,7,2,5,2,7,3,1,2,1,10,3,2,5,2,2,6,2,6,4,7,5,2,6,1,5,1,3,3,2,2,4,1,6,3,5,5,4,2,1,2,2,2,1,4,4,3,4,3,3,3,5,2,2,1,4,2,1,1,6,2,2,4,2,3,2,4,6,3,1,4,1,5,5,2,4,2,1,3,1,2,2,2,3,2,2,3,6,1,1,5,2,1,4,4,5,6,5,2,6,3,3,2,3,4,3,4,2,7,5,2,4,2,5,3,1,2,2,3,4,1,2,3,1,2,1,2,1,3,1,6,8,7,6,3,2,5,5,4,4,3,3,4,5,3,1,1,4,4,2,3,4,2,3,1,2,5,2,3,1,2,3,2,4,2,5,5,5,3,3,2,2,4,5,2,2,4,4,2,1,5,3,3,3,8,4,5,5,3,3,1,2,1,7,3,4,3,2,2,4,3,4,2,5,2,4,1,4,7,2,3,4,4,2,2,4,4,4,4,1,2,4,5,2,1,8,3,4,3,5,6,7,3,1,5,4,2,3,4,3,1,1,4,3,2,5,3,2,3,6,4,1,3,1,6,4,3,1,5,4,2,4,2,1,2,2,6,4,2,3,2,2,3,3,2,4,2,3,2,3,6,2,4,8,2,4,2,4,4,2,4,5,5,3,5,4,1,2,2,1,2,4,1,1,5,6,5,2,3,3,2,2,2,2,4,3,2,2,5,3,3,4,2,1,3,1,1,4,3,2,4,4,3,4,5,4,1,6,1,1,2,3,1,2,6,2,3,3,3,3,4,4,4,2,4,2,5,4,5,3,3,1,3,3,4,6,2,4,5,2,1,5,1,1,1,3,6,3,6,2,6,5,2,2,4,3,3,3,2,3,2,2,3,6,3,4,3,2,3,1,2,2,3,1,5,1,3,3,1,4,4,1,2,3,5,3,1,3,3,1,5,5,3,1,2,4,2,3,1,3,1,2,2,2,4,4,4,2,2,2,2,4,2,2,2,3,3,3,5,2,4,3,2,2,2,3,2,4,3,3,1,6,2,1,1,2,4,3,1,1,1,2,3,5,3,1,2,3,4,6,3,2,3,1,3,2,6,3,5,3,3,3,3,1,5,4,2,2,2,3,2,1,2,4,2,1,3,2,1,6,1,4,3,2,4,4,2,1,1,5,5,4,3,4,2,1,5,4,1,2,3,1,1,4,1,2,5,5,3,1,3,3,2,2,2,2,1,2,3,3,3,1,2,1,1,1,3,1,2,2,2,2,1,3,2,3,1,2,3,1,2,3,3,4,2,4,3,1,1,2,4,4,1,5,2,2,2,4,1,1,2,4,3,4,2,5,4,1,3,2,3,4,2,3,1,4,5,3,2,4,2,4,2,3,5,1,2,2,2,1,2,2,1,2,1,2,2,6,3,2,2,3,2,1,1,2,5,4,1,3,1,1,1,1,5,2,1,2,3,3,1,3,2,6,3,2,1,1,4,1,1,2,2,6,2,2,2,2,5,2,4,1,1,1,3,1,5,1,3,2,4,1,2,1,3,3,1,2,3,1,1,2,3,3,2,7,3,1,1,6,1,1,3,2,3,2,4,1,4,2,2,3,2,4,1,4,4,4,1,4,2,1,4,3,1,2,1,1,3,2,1,1,3,2,2,1,1,1,1,2,2,1,1,3,3,3,3,1,1,4,1,3,1,2,3,2,1,2,3,1,1,1,1,2,2,2,3,2,1,1,2,3,4,1,2,3,1,5,3,3,2,1,2,3,4,2,3,1,3,1,4,1,1,3,2,2,2,2,5,1,2,2,4,2,1,3,1,2,1,4,3,3,2,2,4,2,3,3,3,1,1,3,2,1,3,3,2,2,5,3,2,2,4,2,4,3,1,1,2,1,3,4,1,3,2,2,4,3,2,1,2,3,1,4,2,3,2,3,4,3,1,1,1,3,1,4,1,5,2,1,3,1,2,3,2,1,2,2,2,3,3,4,1,1,1,2,1,3,1,5,4,1,3,1,3,5,2,1,2,1,6,2,2,3,2,1,5,2,4,2,3,1,2,3,1,4,3,2,1,1,5,2,1,3,2,1,6,2,3,1,1,2,2,2,4,1,4,5,1,1,1,5,2,2,2,1,2,2,3,2,1,4,2,2,4,2,5,4,3,3,1,4,1,5,4,1,7,1,2,1,2,1,3,1,2,3,4,4,2,2,5,2,3,1,2,2,2,3,1,4,3,4,1,4,2,2,1,2,1,3,5,4,1,1,5,2,2,2,6,3,2,1,2,2,3,3,1,3,3,2,3,3,4,4,2,1,1,1,3,2,3,2,2,2,3,1,2,1,1,1,1,9,2,4,2,5,1,1,2,3,1,4,3,1,2,1,3,1,3,1,2,1,3,1,4,2,4,1,4,1,2,1,2,4,1,1,2,3,2,3,2,2,3,2,1,3,3,2,1,1,6,3,2,3,4,1,1,2,3,3,1,3,1,1,2,1,1,3,5,2,1,6,2,1,3,2,1,5,1,2,3,3,4,1,1,2,4,4,2,1,4,1,2,1,1,2,3,3,1,2,1,2,4,3,5,1,4,2,1,3,3,4,1,1,3,4,3,3,2,3,1,2,1,1,1,3,1,3,1,1,1,2,3,5,2,3,1,5,1,2,1,1,3,1,1,1,1,2,4,1,6,2,1,1,1,1,1,1,2,4,3,2,2,3,1,3,3,3,1,1,2,3,4,3,1,1,2,2,2,1,1,2,1,1,5,2,1,1,2,2,2,2,2,1,1,2,3,3,3,2,1,3,1,2,2,1,3,2,3,1,4,1,1,1,2,3,1,2,2,1,5,1,1,2,2,1,2,3,5,3,3,2,1,4,2,2,5,2,3,2,1,1,1,2,1,1,2,2,1,2,1,1,1,3,1,1,1,1,1,1,1,2,3,2,4,1,1,1,2,1,3,1,2,1,3,1,1,1,1,2,3,2,1,1,2,1,2,1,2,1,2,2,1,1,1,3,2,1,1,3,1,3,1,1,2,1,1,1,2,2,1,2,3,1,2,2,1,1,1,1,1,1,1,2,1,1,4,1,1,1,1,2,2,1,3,1,3,1,4,2,2,3,2,2,1,1,3,2,2,2,2,2,4,4,3,1,3,1,2,2,1,1,2,1,2,3,2,1,1,2,3,2,1,1,2,1,4,1,5,3,2,1,1,5,1,4,2,1,3,1,1,1,1,2,2,1,4,2,1,2,1,1,3,4,1,1,2,1,3,4,2,3,1,1,1,2,2,3,1,1,1,4,1,2,1,2,3,1,2,1,1,6,2,2,3,2,1,2,1,3,2,1,1,2,3,1,1,1,2,2,2,1,4,1,1,2,5,1,2,2,4,3,2,2,1,5,2,2,2,2,4,3,1,3,1,2,1,2,7,2,2,3,1,2,1,1,1,2,1,1,3,1,1,1,1,1,1,2,1,2,1,3,2,2,3,1,1,3,1,1,1,3,1,1,1,4,3,1,2,3,1,1,1,2,1,2,2,2,1,2,3,1,2,1,1,2,1,3,1,1,1,2,1,1,1,2,1,1,1,1,2,2,2,2,1,2,1,2,1,1,2,2,1,4,2,3,1,3,1,2,1,2,1,1,2,1,1,1,4,3,1,1,2,4,1,1,2,1,1,1,2,3,1,1,1,1,2,2,1,3,1,3,1,3,1,2,1,1,1,2,1,1,2,1,1,1,1,1,1,1,1,1,1,1,1,1,1,1,2,2,1,1,1,2,2,2,1,1,1,1,2,2,2,1,1,1,1,1,1,1,1,1,2,1,2,4,4,1,1,1,1,2,1,2,3,2,3,1,1,3,3,2,1,2,5,1,1,2,3,1,1,1,2,2,1,1,1,4,1,1,1,2,1,1,2,1,2,1,2,2,1,2,1,1,4,1,1,2,1,1,1,1,1,1,2,2,1,2,1,1,3,3,2,3,2,5,1,2,2,2,1,2,3,2,1,1,1,1,2,1,1,1,2,1,1,2,1,2,1,1,1,1,1,1,2,2,2,2,4,1,1,2,2,2,1,4,3,2,1,4,1,1,1,2,1,1,2,2,1,2,2,1,1,1,3,1,2,1,1,2,1,1,2,1,2,2,1,1,3,1,2,1,1,2,1,1,2,1,1,1,1,1,1,1,1,3,1,1,1,3,1,1,4,1,1,1,2,2,1,2,2,4,2,2,1,1,1,1,1,1,1,2,1,1,1,2,2,2,2,1,1,2,1,1,1,1,2,1,2,1,1,1,2,1,1,2,1,1,1,1,1,1,1,2,1,1,1,2,2,1,1,2,1,1,1,1,1,1,1,1,1,1,1,2,1,2,1,1,1,1,1,1,2,2,1,2,2,1,1,1,1,2,3,1,1,1,2,1,2,1,2,1,1,2,1,2,2,1,1,2,2,1,1,2,1,1,1,1,2,3,1,3,2,2,1,1,1,1,1,1,1,1,1,1,2,1,1,1,1,1,3,1,1,3,2,1,1,3,2,1,2,1,2,1,1,1,2,2,1,2,1,1,1,1,1,2,3,2,1,1,1,1,1,1,2,1,1,3,2,1,3,1,1,2,4,1,1,1,2,1,1,1,1,1,1,1,2,1,1,1,1,1,2,1,2,2,1,1,1,1,1,2,1,1,1,2,1,1,1,1,2,2,1,1,2,2,2,1,1,2,1,1,1,1,1,1,1,1,2,5,1,1,2,2,2,1,2,2,1,1,2,1,1,2,1,1,1,1,1,1,1,1,1,2,1,1,3,3,1,1,1,3,1,1,1,1,2,2,3,1,1,1,1,1,2,1,1,2,3,1,1,2,1,1,1,1,1,3,1,1,1,1,1,1,1,3,2,1,1,1,1,1,1,1,2,1,1,1,4,1,2,1,2,1,3,1,1,1,1,1,1,2,2,1,1,1,1,1,2,1,1,1,1,3,2,1,1,1,1,1,2,2,3,1,1,1,2,1,1,1,2,1,1,1,1,1,2,1,1,1,3,1,1,1,1,1,2,1,2,1,1,2,1,1,1,1,1,1,1,1,1,2,1,2,1,1,1,1,1,1,1,1,2,1,1,1,1,2,1,2,1,1,1,2,1,2,1,1,2,1,1,2,2,1,2,1,1,1,1,1,1,2,1,1,1,1,1,1,3,1,1,1,1,1,1,3,1,1,1,1,1,1,1,1,1,1,1,2,2,1,1,1,1,1,1,1,1,1,1,1,2,1,1,1,2,1,1,1,1,1,2,1,1,1,3,1,1,2,1,1,1,2,2,2,1,1,2,1,1,3,1,1,1,2,2,2,1,1,4,1,1,1,1,1,1,1,1,1,2,3,2,1,1,1,2,1,1,1,1,2,1,1,1,1,1,1,1,3,1,1,1,2,1,1,2,1,1,1,1,1,1,1,1,1,1,1,1,2,1,1,2,1,2,1,1,1,1,2,2,2,1,1,1,1,1,1,1,1,2,2,1,1,1,1,1,2,1,1,2,1,1,1,1,1,2,1,1,1,2,1,1,2,1,1,2,1,2,1,1,1,2,1,1,1,1,1,1,1,1,1,1,1,1,1,2,1,1,1,2,1,1,1,1,2,1,2,1,1,1,1,3,1,1,1,2,1,1,1,1,1,1,1,1,1,1,1,1,1,1,1,1,1,1,1,1,1,1,1,1,1,1,1,2,1,1,1,1,1,1,1,1,1,1,1,1,1,1,1,1,1,1,1,2,1,1,1,1,1,1,1,1,1,1,1,1,1,1,1,1,1,1,1,1,1,1,1,1,1,1,1,1,1,1,1,1,3,1,1,1,1,1,1,1,1,1,1,1,1,1,1,1,1,1,1,1,1,1,1,1,1,1,1,1,1,1,1,1,1,1,1,1,1,1,1,1,2,1,1,1,1,1,1,1,1,1,2,1,1,1,2,1,1,1,1,1,1,1,1,1,1,1,3,1,1,1,1,2,1,1,1,1,1,1,1,1,1,1,1,1,1,2,1,1,1,1,1,1,1,1,1,1,1,1,1,2,1,1,1,1,1,1,1,1,1,2,1,1,1,1,1,1,1,1,1,1,1,1,1,1,2,1,1,1,1,1,2,1,2,1,1,2,1,1,1,1,1,1,1,1,1,1,1,1,1,1,1,1,1,1,1,1,1,1,1,1,1,1,1,1,1,1,1,1,1,1,1,1,1,1,1,1,1,1,1,1,1,1,1,1,1,1,1,1,1,1,1,1,1,1,1,1,1,1,1,1,1,1,1,1,1,1,1,1,1,1,1,1,1,2,1,2,1,1,1,1,1,1,1,1,1,1,1,1,1,2,1,1,1,1,1,1,1,1,1,1,1,1,1,1,1,1,1,1,1,1,1,1,1,1,1,1,1,1,1,1,1,1,1,1,1,1,1,1,1,1,1,1,1,1,1,1,1,1,1,1,1,1,1,1,1,1,1,1,1,1,1,2,1,1,1,1,1,1,1,1,1,1,1,1,1,1,1,1,1,2,1,1,1,1,1,1,1,1,1,1,1,1,1,1,1,1,1,1,1,1,1,2,1,1,1,1,1,1,1,1,1,1,1,1,1,1,1,1,1,1,1,1,1,1,1,1,1,1,1,1,1,1,1,1,1,1,1,1,1,1,1,1,1,1,1,1,1,1,1,1,1,1,1,1,1,1,1,1,1,1,1,1,1,1,1,1,1,1,1,1,1,1,1,1,1,1,1,1,1,1,1,1,1,1,1,1,1,1,1,1,1,1,1,1,1,1,1,1,1,1,1,1,1,1,1,1,1,1,1,1,1,2,1,1,1,1,1,1,1,1,1,1,1,1,1,1,1,1,1,1,1,1,1,1,1,1,1,1,1,1,1,1,1,1,1,1,1,1,1,1,1,1,1,1,1,1,1,1,1,1,1,1,1,1,1,1,1,1,1,1,1,1,1,1,1,1,1,1,1,1,1,1,1,1,1,1,1,1,1,1,1 |
```


---


 **Insertions and deletions length:**

```
|  |  |
| --- | --- |
| Min | 0 |
| Max | 62 |
| Mean | 1.747 |
| Median | 1 |
| Standard deviation | 4.247 |
| Values | 0,1,2,3,4,5,6,7,8,9,10,11,12,13,14,15,16,17,18,19,20,21,22,23,24,25,26,27,28,29,30,31,32,33,34,35,36,37,38,39,41,42,43,44,45,46,47,48,49,50,51,52,53,54,55,61,62 |
| Count | 8848,13011,814,566,320,353,201,169,206,138,73,181,60,70,95,41,38,94,31,51,46,24,16,59,19,10,21,14,4,28,5,7,8,4,9,11,2,6,7,7,7,5,1,4,3,1,5,3,3,4,2,3,2,2,1,1,1 |
```


---


 **Base changes (SNPs)** 

|  |  |  |  |  |
| --- | --- | --- | --- | --- |
|  | **A** | **C** | **G** | **T** |
| **A** | 0 | 8,804 | 58,656 | 8,269 |
| **C** | 10,441 | 0 | 7,767 | 64,283 |
| **G** | 64,368 | 7,523 | 0 | 10,587 |
| **T** | 8,253 | 59,038 | 8,976 | 0 |

---


  **Ts/Tv (transitions / transversions)** 

**Note:** Only SNPs are used for this statistic.  
**Note:** This Ts/Tv ratio is a 'raw' ratio (ratio of observed events).

|  |  |
| --- | --- |
| Transitions | 490,741 |
| Transversions | 167,376 |
| Ts/Tv ratio | 2.932 |

**All variants:**

```
Sample ,KS17,CMW560,CMW1803,UG27,UG10,CMW567,Total
Transitions ,76722,89962,80471,78977,79203,85406,490741
Transversions ,26475,30474,26986,27183,27190,29068,167376
Ts/Tv ,2,898,2,952,2,982,2,905,2,913,2,938,2,932
```

**Only known variants** (i.e. the ones having a non-empty ID field):

```
No results available (empty input?)
```

---


  **Allele frequency** 
  

|  |  |
| --- | --- |
| Min | 8 |
| Max | 50 |
| Mean | 26.156 |
| Median | 25 |
| Standard deviation | 16.102 |
| Values | 8,10,12,16,20,25,30,33,37,40,41,50 |
| Count | 107049,6301,3806,64994,5935,45936,4294,36938,2793,3855,29483,86320 |

---


  **Allele Count** 
  

|  |  |
| --- | --- |
| Min | 1 |
| Max | 6 |
| Mean | 2.931 |
| Median | 2 |
| Standard deviation | 1.835 |
| Values | 1,2,3,4,5,6 |
| Count | 126551,78336,51774,41566,35929,63548 |

---


  **Hom/Het per sample** 
  
  
  

```
Sample_names , KS17, CMW560, CMW1803, UG27, UG10, CMW567
Reference , 186752, 166466, 188409, 185683, 184285, 175821
Het , 183353, 209058, 190151, 191317, 192181, 199682
Hom , 0, 0, 0, 0, 0, 0
Missing , 27599, 22180, 19144, 20704, 21238, 22201
```

---


 **Codon changes**

How to read this table:   
- Rows are reference codons and columns are changed codons. E.g. Row 'AAA' column 'TAA' indicates how many 'AAA' codons have been replaced by 'TAA' codons.  
- Red background colors indicate that more changes happened (heat-map).  
- Diagonals are indicated using grey background color   
- WARNING: This table may include different translation codon tables (e.g. mamalian DNA and mitochondrial DNA).

|  | - | AAA | AAC | AAG | AAT | ACA | ACC | ACG | ACT | AGA | AGC | AGG | AGT | ATA | ATC | ATG | ATT | CAA | CAC | CAG | CAT | CCA | CCC | CCG | CCT | CGA | CGC | CGG | CGT | CTA | CTC | CTG | CTT | GAA | GAC | GAG | GAT | GCA | GCC | GCG | GCT | GGA | GGC | GGG | GGT | GTA | GTC | GTG | GTT | TAA | TAC | TAG | TAT | TCA | TCC | TCG | TCT | TGA | TGC | TGG | TGT | TTA | TTC | TTG | TTT |
| --- | --- | --- | --- | --- | --- | --- | --- | --- | --- | --- | --- | --- | --- | --- | --- | --- | --- | --- | --- | --- | --- | --- | --- | --- | --- | --- | --- | --- | --- | --- | --- | --- | --- | --- | --- | --- | --- | --- | --- | --- | --- | --- | --- | --- | --- | --- | --- | --- | --- | --- | --- | --- | --- | --- | --- | --- | --- | --- | --- | --- | --- | --- | --- | --- | --- |
| - |  | 30 | 59 | 113 | 37 | 42 | 51 | 26 | 44 | 18 | 49 | 19 | 28 | 8 | 32 | 20 | 19 | 64 | 44 | 90 | 23 | 64 | 57 | 28 | 65 | 12 | 16 | 10 | 18 | 11 | 32 | 20 | 15 | 102 | 115 | 186 | 72 | 63 | 55 | 21 | 92 | 44 | 104 | 21 | 81 | 6 | 43 | 11 | 22 | 4 | 19 | 3 | 15 | 34 | 66 | 35 | 76 | 6 | 16 | 22 | 4 | 7 | 35 | 12 | 24 |
| AAA | 122 | 83 | 178 | 1,377 | 89 | 103 | 1 | 4 | 3 | 296 | 8 | 28 | 6 | 38 | 1 | 7 | 1 | 158 | 3 | 8 | 1 | 5 |  |  | 1 | 5 | 1 |  |  | 1 | 1 |  | 1 | 411 | 14 | 24 | 5 | 3 | 1 | 1 |  | 11 | 3 | 4 |  | 2 |  | 1 | 1 | 12 | 1 |  | 2 |  |  |  |  |  |  |  |  |  |  | 1 |  |
| AAC | 102 | 196 | 114 | 147 | 1,557 | 4 | 118 | 2 | 10 | 7 | 518 | 6 | 35 | 1 | 47 |  | 6 | 2 | 80 | 5 | 5 | 1 | 1 |  |  | 1 | 3 | 1 | 1 |  | 3 |  |  | 9 | 493 | 8 | 42 |  | 5 |  | 1 | 1 | 20 |  |  |  | 3 |  |  | 1 | 45 |  | 4 |  | 4 |  | 1 |  | 3 |  |  |  |  | 1 | 1 |
| AAG | 225 | 1,644 | 154 | 146 | 257 | 12 | 3 | 108 | 4 | 54 | 6 | 473 | 11 | 2 | 4 | 100 | 4 | 20 | 6 | 185 | 5 |  |  | 2 | 1 | 2 |  | 5 | 2 |  |  | 5 |  | 25 | 9 | 596 | 13 |  | 2 | 7 | 4 | 2 |  | 9 | 4 |  |  | 3 | 1 | 3 | 2 | 18 | 2 |  |  | 4 |  |  |  | 1 |  |  |  | 1 |  |
| AAT | 63 | 97 | 1,522 | 156 | 86 | 6 | 7 | 3 | 90 | 3 | 34 | 2 | 289 | 1 | 6 | 1 | 58 | 4 | 7 | 4 | 100 |  |  | 1 | 1 |  | 2 |  |  |  |  |  | 2 | 9 | 39 | 8 | 504 | 1 |  | 1 | 9 |  | 3 |  | 20 | 1 | 3 |  | 6 |  | 3 |  | 45 |  | 1 | 1 | 1 |  | 2 |  |  |  |  |  | 2 |
| ACA | 77 | 117 | 7 | 7 | 3 | 54 | 370 | 906 | 267 | 68 | 5 | 2 | 3 | 377 | 12 | 19 | 2 | 7 |  | 3 | 1 | 65 |  | 2 | 1 | 5 |  |  |  | 2 | 1 | 1 |  | 10 |  | 3 |  | 428 | 13 | 20 | 4 | 2 | 1 | 2 |  | 15 | 1 | 4 |  |  |  | 1 |  | 69 | 5 | 12 | 11 |  |  |  | 1 | 9 |  | 1 |  |
| ACC | 125 | 7 | 138 | 4 | 10 | 402 | 59 | 323 | 1,199 | 1 | 116 | 2 | 11 | 11 | 337 | 7 | 20 | 2 | 1 |  |  |  | 72 | 2 | 3 |  | 1 |  |  |  | 7 |  |  | 5 | 4 | 1 | 2 | 8 | 512 | 4 | 29 |  | 7 |  | 2 |  | 18 | 1 | 5 |  | 2 |  | 1 | 3 | 83 | 2 | 5 |  | 2 |  | 1 | 1 | 3 | 1 | 1 |
| ACG | 46 | 6 | 4 | 116 | 6 | 1,021 | 252 | 34 | 292 | 5 |  | 33 | 1 | 32 | 7 | 276 | 8 |  |  | 5 |  | 3 | 1 | 35 |  |  |  |  | 1 |  | 2 | 3 |  | 2 |  | 8 | 1 | 17 | 7 | 277 | 10 | 1 |  | 1 |  | 2 | 1 | 6 |  |  | 1 |  |  | 4 | 2 | 56 | 2 |  |  |  |  | 1 |  | 3 |  |
| ACT | 115 | 5 | 16 | 7 | 155 | 295 | 1,150 | 297 | 72 | 3 | 14 | 1 | 131 | 4 | 28 | 11 | 275 |  |  |  | 4 | 2 |  | 1 | 77 |  |  |  | 3 |  |  |  | 6 | 2 | 3 | 1 | 5 | 6 | 25 | 6 | 486 |  | 1 |  | 3 | 1 | 2 | 2 | 11 |  |  | 1 |  |  | 7 |  | 70 |  |  |  | 1 |  |  | 2 | 2 |
| AGA | 58 | 314 | 12 | 34 | 5 | 60 |  |  | 1 | 29 | 61 | 502 | 20 | 46 |  | 2 | 1 | 6 | 1 | 2 | 1 | 3 |  |  | 1 | 207 | 5 | 19 | 4 |  |  |  |  | 6 |  | 2 | 2 | 6 |  | 1 |  | 192 | 1 | 10 | 1 | 2 |  | 1 | 1 |  | 1 | 2 |  |  |  | 2 | 1 | 12 | 1 |  | 1 |  |  |  |  |
| AGC | 83 | 12 | 445 | 10 | 27 | 6 | 98 | 2 | 21 | 90 | 49 | 37 | 941 | 1 | 54 | 4 | 7 |  | 6 | 4 | 2 |  | 2 |  | 1 |  | 49 | 2 | 7 |  | 2 |  |  | 1 | 24 | 1 | 2 | 1 | 7 |  |  | 2 | 337 | 6 | 27 | 1 | 2 |  | 1 |  |  |  |  |  | 9 |  | 1 |  | 35 | 1 | 5 |  |  |  |  |
| AGG | 45 | 33 | 2 | 416 | 6 | 5 | 2 | 33 |  | 531 | 44 | 22 | 53 | 3 | 1 | 51 |  | 2 |  | 9 |  |  |  |  |  | 19 | 10 | 113 | 2 |  |  | 1 |  | 2 | 1 | 12 | 1 |  |  | 3 |  | 3 | 5 | 121 |  |  |  | 6 |  | 1 |  |  |  |  |  |  |  | 1 |  | 30 | 2 |  |  |  |  |
| AGT | 43 | 7 | 30 | 15 | 269 | 7 | 15 | 1 | 103 | 45 | 901 | 51 | 48 | 1 | 7 | 1 | 52 | 2 |  | 3 | 8 |  |  |  | 2 | 1 | 4 | 1 | 44 |  |  |  | 3 |  | 3 | 1 | 13 |  | 1 | 2 | 14 | 1 | 26 | 6 | 290 |  | 2 |  | 9 |  |  |  | 1 |  |  | 1 | 6 |  | 1 |  | 39 |  |  | 1 |  |
| ATA | 24 | 38 |  | 2 | 5 | 267 | 5 | 6 | 3 | 43 | 1 | 4 |  | 32 | 379 | 344 | 173 | 2 |  | 1 |  | 4 |  |  |  | 1 |  | 1 |  | 63 | 4 | 11 | 3 | 8 |  | 2 |  | 16 | 1 | 1 | 1 | 2 |  |  |  | 213 | 10 | 9 | 6 |  | 1 |  |  | 2 | 1 |  | 1 |  | 1 |  |  | 48 | 2 | 6 | 2 |
| ATC | 82 | 3 | 53 | 2 | 7 | 4 | 334 | 6 | 18 |  | 52 |  | 4 | 499 | 102 | 138 | 1,938 |  |  | 1 |  |  | 1 |  |  |  | 2 |  |  | 2 | 146 | 4 | 21 |  | 4 |  | 1 | 1 | 17 |  | 1 | 2 | 2 |  | 1 | 9 | 744 | 10 | 69 |  |  |  | 2 |  | 3 |  |  |  | 3 |  |  | 2 | 68 | 4 | 7 |
| ATG | 75 | 6 | 1 | 78 | 7 | 18 | 7 | 258 | 9 | 3 | 3 | 61 | 5 | 498 | 132 | 83 | 153 | 1 |  | 7 | 1 | 1 |  | 1 | 1 |  |  |  |  | 11 | 5 | 119 | 5 | 2 |  | 5 | 1 | 1 | 1 | 15 | 2 |  | 1 |  | 1 | 14 | 11 | 287 | 2 | 1 |  |  | 1 | 1 | 1 | 3 |  |  |  | 1 |  | 9 |  | 100 | 1 |
| ATT | 40 | 1 | 5 |  | 57 | 3 | 25 | 9 | 245 |  | 3 |  | 56 | 191 | 1,854 | 122 | 71 | 1 |  |  | 3 |  | 2 |  |  |  |  |  |  | 3 | 7 | 5 | 156 |  |  |  | 5 |  | 2 |  | 16 | 1 |  |  | 7 | 6 | 70 | 16 | 623 |  |  |  | 2 | 2 |  |  | 7 |  |  |  | 1 |  | 4 | 2 | 49 |
| CAA | 134 | 198 | 2 | 12 | 2 | 7 | 1 |  |  | 6 |  |  | 1 | 2 |  |  |  | 70 | 111 | 1,240 | 83 | 82 | 5 | 4 | 6 | 275 | 7 | 21 | 3 | 35 |  | 6 | 4 | 213 | 2 | 25 | 6 | 4 |  |  |  | 6 |  |  |  | 5 |  |  |  | 214 |  | 9 | 1 | 1 |  | 1 | 1 | 4 |  |  |  | 1 |  | 1 | 1 |
| CAC | 42 | 2 | 83 |  | 6 |  | 4 |  | 2 | 1 | 5 | 1 | 1 |  | 2 | 1 |  | 111 | 32 | 97 | 911 | 1 | 47 |  | 4 | 4 | 222 | 5 | 5 | 3 | 40 | 2 | 5 | 5 | 71 |  | 3 | 1 | 1 |  | 1 | 1 | 1 |  |  | 1 |  |  |  |  | 286 |  | 30 | 1 | 3 |  |  |  | 6 |  | 3 |  | 2 |  | 1 |
| CAG | 153 | 13 | 7 | 184 | 3 | 3 | 1 | 2 | 1 | 1 |  | 2 |  |  |  | 1 |  | 1,301 | 99 | 83 | 148 | 7 | 1 | 62 | 6 | 23 | 1 | 239 | 1 | 4 | 3 | 76 | 2 | 17 | 4 | 210 | 4 | 1 |  | 2 | 2 |  |  | 3 |  |  |  |  |  | 10 | 2 | 256 | 4 | 2 |  | 2 |  |  |  | 3 |  | 1 |  | 2 |  |
| CAT | 37 |  | 10 | 3 | 123 | 1 |  |  | 2 |  |  |  | 3 |  |  |  |  | 84 | 838 | 115 | 65 | 1 |  |  | 55 | 4 | 16 | 7 | 163 | 2 | 6 |  | 52 | 3 | 10 | 3 | 106 |  |  |  | 2 |  |  |  | 5 | 1 |  |  | 1 |  | 18 | 3 | 335 | 1 |  |  |  |  |  | 1 | 4 |  |  |  | 2 |
| CCA | 114 | 3 | 2 |  |  | 65 | 2 | 9 |  | 1 |  |  |  | 4 |  |  | 3 | 90 | 6 | 9 | 4 | 66 | 314 | 856 | 279 | 51 | 2 | 1 |  | 353 | 3 | 8 | 4 | 1 |  |  |  | 63 | 3 | 4 | 3 | 1 | 1 |  |  | 2 |  |  |  | 1 | 1 |  |  | 343 | 10 | 12 | 5 | 1 |  |  |  | 11 |  | 2 | 1 |
| CCC | 213 |  | 1 |  |  | 1 | 73 | 3 | 7 |  | 5 | 1 |  |  |  |  | 1 | 2 | 66 |  | 5 | 349 | 65 | 207 | 1,206 | 3 | 34 | 3 | 2 | 3 | 178 | 1 | 12 |  | 4 |  |  | 4 | 61 | 1 | 6 | 1 | 2 |  |  |  | 4 |  |  |  | 2 |  | 1 | 3 | 267 | 2 | 26 |  | 3 |  | 2 |  | 7 |  | 1 |
| CCG | 62 |  |  | 2 | 2 | 2 | 1 | 36 | 4 |  |  |  |  |  |  | 1 | 1 | 6 | 1 | 58 |  | 841 | 232 | 32 | 255 | 3 |  | 23 |  | 7 | 3 | 198 |  | 1 |  | 1 |  | 5 | 1 | 41 | 5 | 1 |  |  |  |  |  | 1 |  |  |  |  |  | 13 | 3 | 163 | 7 |  |  | 2 |  |  |  | 16 |  |
| CCT | 167 |  |  | 1 | 4 | 3 | 6 | 4 | 110 |  |  |  | 1 |  |  | 1 | 4 | 3 | 3 | 3 | 95 | 293 | 1,085 | 255 | 96 |  | 5 |  | 54 | 1 | 9 | 3 | 250 |  |  |  | 3 | 1 | 5 |  | 84 |  |  |  |  |  |  |  | 4 |  |  |  | 1 | 5 | 18 | 7 | 356 |  | 1 |  | 2 |  | 1 | 1 | 3 |
| CGA | 54 | 4 |  | 1 | 1 | 3 |  |  |  | 221 |  | 22 | 2 |  |  |  |  | 380 | 4 | 21 | 5 | 37 |  | 6 | 1 | 29 | 181 | 499 | 107 | 36 | 2 | 1 |  | 3 |  |  |  | 1 |  | 1 | 1 | 35 | 1 | 3 | 1 | 2 |  |  |  | 16 |  |  |  | 1 |  | 1 |  | 124 | 2 | 1 |  | 2 |  | 1 |  |
| CGC | 54 | 2 | 4 |  | 2 |  |  |  |  | 10 | 64 | 6 | 6 |  | 1 |  | 1 | 7 | 214 | 5 | 14 | 2 | 25 |  | 3 | 180 | 34 | 148 | 866 | 1 | 46 | 2 | 6 |  | 2 |  |  |  | 1 | 1 |  | 3 | 55 | 1 | 6 |  | 1 |  |  | 1 | 6 |  |  |  |  |  |  |  | 201 | 2 | 5 |  | 1 |  |  |
| CGG | 32 |  | 1 | 6 | 1 |  |  | 1 |  | 17 | 2 | 111 | 1 |  |  | 1 |  | 17 | 5 | 262 | 4 | 2 |  | 24 |  | 472 | 117 | 14 | 133 |  |  | 35 |  | 2 | 1 | 3 | 1 |  |  |  |  | 3 | 1 | 36 | 1 | 1 |  |  |  |  |  | 5 |  |  |  |  |  | 2 | 1 | 119 | 3 |  |  |  |  |
| CGT | 31 |  |  |  | 5 |  |  |  |  | 5 | 2 | 4 | 38 |  |  |  |  | 7 | 12 | 6 | 229 | 1 | 3 |  | 40 | 105 | 773 | 110 | 31 |  | 5 | 3 | 54 |  |  |  | 4 |  |  |  | 1 |  | 2 | 1 | 32 |  |  |  |  |  | 1 |  | 6 |  |  |  |  | 3 | 7 | 1 | 172 | 1 |  |  | 1 |
| CTA | 31 |  |  |  |  | 1 |  |  |  | 2 |  |  | 1 | 90 | 4 | 14 | 1 | 28 | 2 | 4 | 2 | 154 | 6 | 13 | 5 | 45 | 1 | 2 | 1 | 23 | 320 | 873 | 201 | 2 |  | 2 |  | 1 | 1 |  |  |  |  |  |  | 25 | 1 | 2 | 5 | 1 |  | 1 |  | 12 |  | 1 |  |  |  | 2 |  | 395 | 10 | 97 | 2 |
| CTC | 116 |  | 2 | 1 | 1 |  | 2 |  | 3 | 2 | 1 |  |  | 3 | 167 | 2 | 8 |  | 41 | 2 | 3 | 3 | 151 |  | 20 | 2 | 43 |  | 8 | 409 | 86 | 443 | 1,780 |  | 2 |  |  |  |  | 1 | 3 |  |  |  |  | 1 | 123 | 1 | 7 |  | 5 |  | 2 |  | 18 |  | 2 | 1 | 1 |  |  | 14 | 333 | 36 | 22 |
| CTG | 51 |  |  | 1 | 2 |  |  | 1 |  | 1 | 1 | 1 |  | 13 | 1 | 145 | 3 | 4 | 1 | 58 | 2 | 8 | 4 | 151 | 4 | 7 |  | 32 |  | 1,016 | 400 | 58 | 424 |  |  | 3 |  |  |  | 3 |  |  | 1 | 2 |  | 4 | 6 | 69 |  |  |  |  |  | 2 |  | 5 |  |  |  | 3 |  | 88 | 6 | 971 | 7 |
| CTT | 68 | 1 | 1 |  | 2 | 1 |  | 1 | 8 | 1 |  |  | 1 | 2 | 8 | 3 | 183 | 1 | 5 | 6 | 48 |  | 11 | 4 | 185 | 3 | 2 | 2 | 47 | 218 | 1,541 | 402 | 89 |  | 1 | 2 | 2 |  | 1 |  | 8 |  |  |  | 3 | 1 | 11 | 1 | 105 |  | 1 |  | 2 |  |  |  | 9 |  |  | 1 | 3 | 18 | 22 | 36 | 324 |
| GAA | 211 | 611 | 11 | 37 | 7 | 13 | 1 |  |  | 15 | 1 | 1 | 2 | 11 | 1 | 2 |  | 188 | 3 | 16 | 1 | 3 | 1 |  | 1 | 7 | 1 |  |  |  |  |  |  | 109 | 284 | 1,603 | 210 | 122 | 4 | 3 | 7 | 359 | 8 | 21 | 6 | 44 | 2 | 3 | 1 | 54 |  | 2 | 2 | 7 |  | 1 | 3 |  |  |  |  | 3 |  |  |  |
| GAC | 159 | 14 | 639 | 12 | 55 | 1 | 6 |  | 2 | 1 | 21 |  | 4 |  | 3 | 1 |  | 3 | 65 | 6 | 8 |  | 1 |  |  | 1 | 5 |  |  |  |  |  | 1 | 350 | 111 | 261 | 2,307 | 5 | 74 | 1 | 6 | 16 | 560 | 5 | 24 |  | 35 | 1 | 7 |  | 77 |  | 3 |  | 9 | 1 |  | 1 | 2 | 2 |  |  | 1 |  |  |
| GAG | 322 | 32 | 12 | 702 | 10 |  | 3 | 7 | 1 | 2 | 1 | 14 | 5 | 1 |  | 5 |  | 19 |  | 222 | 5 | 1 |  | 1 |  | 2 |  | 9 |  |  |  | 2 |  | 1,768 | 275 | 171 | 303 | 10 | 2 | 57 | 5 | 25 | 6 | 309 | 7 | 3 | 1 | 67 | 1 | 3 | 2 | 48 | 2 |  |  | 6 |  |  |  | 1 |  |  | 2 | 2 | 1 |
| GAT | 184 | 6 | 46 | 6 | 769 | 1 | 2 | 1 | 8 |  | 2 | 1 | 8 |  |  | 1 | 2 | 3 | 13 | 4 | 126 |  |  |  | 2 | 2 | 1 |  | 2 |  |  |  | 1 | 193 | 2,183 | 259 | 177 | 1 | 7 | 1 | 96 | 7 | 30 | 2 | 368 | 2 | 2 | 3 | 70 |  | 3 |  | 143 | 1 |  |  | 3 |  |  |  | 1 |  |  | 1 | 3 |
| GCA | 93 | 13 |  | 2 |  | 527 | 18 | 22 | 12 | 3 | 1 | 1 |  | 19 | 3 | 1 |  |  |  | 2 |  | 79 |  | 2 |  | 1 |  |  |  | 3 |  | 1 | 1 | 132 | 6 | 13 | 1 | 55 | 419 | 947 | 334 | 93 | 1 | 6 | 2 | 437 | 9 | 14 | 3 |  |  |  |  | 144 | 3 | 12 | 1 | 1 | 3 | 1 |  | 7 |  |  |  |
| GCC | 114 | 1 | 11 |  |  | 21 | 590 | 11 | 33 | 2 | 11 |  | 1 |  | 15 | 1 | 1 |  | 1 |  |  | 5 | 63 | 1 | 6 |  | 1 |  |  |  | 2 |  |  | 5 | 92 | 4 | 9 | 460 | 82 | 335 | 1,765 | 2 | 89 | 1 | 10 | 10 | 504 | 9 | 19 |  | 1 |  |  | 3 | 171 | 7 | 10 |  | 6 |  | 1 |  | 5 |  | 1 |
| GCG | 50 | 1 | 1 | 4 |  | 28 | 4 | 258 | 8 |  |  | 2 | 1 | 2 |  | 13 | 4 | 1 |  | 1 |  | 3 |  | 35 | 2 |  |  |  |  |  |  | 5 |  | 8 | 3 | 89 | 2 | 1,100 | 378 | 39 | 382 | 5 |  | 52 | 1 | 23 | 10 | 304 | 12 |  |  |  | 1 | 4 | 2 | 68 | 2 |  |  | 1 |  | 1 |  | 3 |  |
| GCT | 166 | 1 | 1 |  | 8 | 9 | 33 | 10 | 616 |  | 2 |  | 6 | 1 | 1 |  | 14 |  |  | 2 | 2 | 4 | 9 | 2 | 91 | 1 | 1 |  | 3 |  | 1 |  | 8 | 4 | 9 | 9 | 129 | 349 | 1,559 | 362 | 108 | 3 | 12 | 1 | 94 | 6 | 25 | 6 | 472 |  |  |  | 2 | 5 | 11 | 3 | 232 |  |  |  | 2 | 1 | 1 | 1 | 3 |
| GGA | 104 | 16 | 1 | 3 |  | 3 | 1 | 2 |  | 299 | 6 | 10 | 3 |  |  | 1 |  | 10 | 1 | 2 | 2 | 3 |  |  |  | 53 | 2 |  |  | 1 |  |  |  | 317 | 7 | 28 | 3 | 104 | 2 | 6 | 5 | 71 | 385 | 917 | 280 | 49 | 1 | 5 |  | 3 |  |  |  | 1 |  | 1 |  | 31 |  | 1 |  |  |  | 1 |  |
| GGC | 180 |  | 29 | 1 | 6 |  | 8 |  | 2 | 2 | 487 | 4 | 25 |  | 2 |  | 1 |  | 4 | 1 | 2 |  |  | 1 | 3 |  | 44 | 1 | 2 |  |  |  | 1 | 9 | 511 | 11 | 29 | 2 | 100 | 1 | 12 | 453 | 95 | 299 | 2,054 |  | 57 |  | 7 |  | 6 |  |  |  | 4 | 1 | 1 |  | 70 | 1 | 6 |  |  |  |  |
| GGG | 85 |  | 3 | 9 | 1 |  |  | 5 |  | 11 | 3 | 180 | 6 |  |  | 4 |  |  |  | 4 | 1 |  |  |  |  | 4 | 2 | 13 |  |  |  |  |  | 26 | 8 | 254 | 8 | 3 | 2 | 51 | 2 | 869 | 271 | 42 | 324 | 1 | 3 | 36 | 2 |  |  | 2 |  |  |  |  |  | 1 |  | 38 | 2 |  |  | 1 |  |
| GGT | 145 |  | 4 | 2 | 10 | 1 | 3 |  | 7 | 2 | 26 | 5 | 394 | 1 | 2 |  | 6 |  |  |  | 5 |  |  |  | 1 |  | 5 | 1 | 42 |  |  |  |  | 7 | 23 | 3 | 360 | 1 | 8 | 2 | 96 | 276 | 1,873 | 279 | 67 | 1 | 3 | 1 | 82 |  | 1 | 1 | 4 |  | 1 |  | 3 |  | 2 | 1 | 56 |  |  |  |  |
| GTA | 19 | 3 |  | 1 |  | 11 |  | 2 |  | 2 |  |  |  | 290 | 14 | 17 | 10 | 4 |  |  |  |  |  |  | 1 |  |  | 1 |  | 43 | 2 | 7 | 1 | 41 | 1 | 6 | 6 | 302 | 6 | 9 | 2 | 35 | 1 | 1 |  | 15 | 280 | 598 | 166 |  |  |  | 1 | 5 |  |  |  |  |  |  |  | 55 | 5 | 3 |  |
| GTC | 77 |  | 4 | 1 | 2 | 5 | 18 |  | 2 |  | 3 | 1 |  | 12 | 851 | 10 | 63 |  |  |  |  | 1 | 3 | 1 | 2 |  |  |  |  | 1 | 121 | 3 | 8 | 4 | 48 | 2 | 6 | 6 | 555 | 4 | 31 |  | 45 |  | 6 | 311 | 72 | 341 | 1,498 |  |  |  | 1 |  | 5 | 2 | 1 |  | 1 |  |  | 1 | 114 | 4 | 5 |
| GTG | 36 | 1 |  |  |  | 1 |  | 14 | 1 |  |  |  |  | 47 | 14 | 300 | 10 |  |  | 3 |  |  |  | 1 |  |  |  |  |  | 5 | 4 | 71 | 1 | 1 | 1 | 59 | 5 | 15 | 3 | 233 | 8 | 2 | 2 | 41 |  | 678 | 318 | 39 | 339 |  |  |  |  |  | 1 | 5 |  |  |  |  |  | 4 | 2 | 83 | 1 |
| GTT | 60 | 1 |  | 1 | 6 | 1 | 4 |  | 14 |  | 1 |  | 2 | 6 | 58 | 8 | 698 |  | 1 | 1 | 5 |  | 1 |  | 3 |  |  |  | 1 | 1 | 9 | 1 | 101 | 2 | 3 | 3 | 67 | 5 | 17 | 8 | 427 | 2 | 5 |  | 54 | 183 | 1,446 | 296 | 81 |  |  |  | 1 |  | 2 |  | 11 |  |  | 1 | 3 | 3 | 5 | 1 | 101 |
| TAA | 8 | 6 |  |  |  |  |  |  |  |  |  |  |  |  |  |  |  | 29 |  |  |  |  |  |  |  | 3 |  |  |  |  |  |  |  | 11 |  |  |  |  |  |  |  |  |  |  |  |  |  | 1 |  | 9 | 7 | 47 | 3 | 3 |  |  |  | 44 |  | 1 |  | 4 | 1 | 2 |  |
| TAC | 46 | 1 | 38 |  | 4 |  | 1 | 1 |  |  |  |  | 1 |  | 1 |  |  | 2 | 222 |  | 14 |  | 1 |  |  | 1 | 5 |  |  | 1 |  |  | 2 | 1 | 63 |  | 7 |  |  |  |  | 1 | 1 |  |  |  |  |  |  | 37 | 80 | 14 | 1,244 | 1 | 31 |  | 4 |  | 172 | 2 | 11 | 1 | 77 |  | 13 |
| TAG | 6 | 2 |  | 5 |  |  |  |  |  |  |  |  |  |  |  |  |  |  | 1 | 27 |  |  |  |  |  |  |  |  |  |  |  |  |  |  |  | 11 |  |  |  |  |  | 1 |  |  |  |  |  |  |  | 30 | 7 | 8 | 2 |  |  | 4 |  | 4 | 1 | 30 |  | 1 |  |  |  |
| TAT | 35 |  |  |  | 33 |  |  |  |  |  | 2 |  |  |  | 1 |  | 3 | 4 | 20 | 5 | 239 |  |  |  | 2 |  |  |  | 5 | 1 |  | 1 | 6 | 3 | 4 | 2 | 85 |  |  | 1 | 1 |  |  |  | 3 |  |  |  | 2 | 8 | 1,092 | 17 | 66 |  | 4 | 2 | 53 | 2 | 16 | 5 | 123 | 1 | 14 | 2 | 80 |
| TCA | 66 | 4 | 1 | 1 |  | 84 | 4 | 8 | 3 | 4 | 1 |  | 2 | 7 | 1 |  |  | 7 |  | 2 |  | 317 | 3 | 13 | 7 | 1 | 1 | 1 |  | 8 | 1 | 1 | 1 | 1 |  | 1 | 2 | 131 | 3 | 13 | 7 | 3 |  |  | 3 | 4 |  | 1 | 1 | 24 | 2 |  | 1 | 78 | 272 | 921 | 222 | 13 | 1 |  | 1 | 291 | 7 | 15 | 4 |
| TCC | 149 |  | 2 |  | 1 | 3 | 82 | 1 | 13 | 1 | 4 |  |  |  | 4 |  |  |  | 6 |  | 1 | 9 | 252 | 7 | 16 | 1 | 3 |  | 1 | 1 | 5 |  | 2 |  | 4 |  |  | 4 | 136 | 3 | 11 | 1 | 1 |  | 1 | 1 | 1 |  |  | 1 | 59 | 1 | 4 | 289 | 56 | 247 | 1,039 |  | 41 |  | 5 | 6 | 224 | 4 | 18 |
| TCG | 52 |  | 1 | 2 |  | 5 | 1 | 48 | 1 |  |  |  |  |  |  | 5 |  | 2 | 2 | 1 |  | 13 | 1 | 135 | 4 | 1 |  |  |  |  | 1 | 7 |  |  |  | 1 |  | 6 | 6 | 77 | 6 |  |  | 1 |  | 2 | 1 | 3 |  |  | 3 | 24 |  | 979 | 260 | 30 | 284 | 1 |  | 34 | 1 | 22 | 4 | 281 | 3 |
| TCT | 134 |  |  |  |  |  | 14 | 2 | 71 |  | 2 |  | 7 |  |  | 1 | 5 |  | 1 |  | 4 | 4 | 15 | 2 | 269 |  |  |  | 4 |  | 1 | 1 | 8 |  | 2 | 2 | 5 | 2 | 10 | 7 | 173 |  | 1 |  | 2 |  | 1 |  | 1 |  | 5 |  | 80 | 240 | 970 | 272 | 74 | 1 | 3 | 2 | 69 | 2 | 12 | 7 | 210 |
| TGA | 6 |  |  |  |  | 1 |  |  |  | 3 |  |  |  |  |  | 1 |  | 2 |  |  |  |  |  |  |  | 24 |  |  |  | 1 | 1 | 1 |  | 2 |  |  |  | 1 |  |  |  | 1 |  |  |  |  |  |  |  | 33 |  | 4 |  | 3 |  |  |  | 6 | 4 | 28 | 4 | 4 | 1 | 1 |  |
| TGC | 31 |  | 3 |  |  |  |  |  |  |  | 17 |  | 2 |  | 1 |  |  |  | 3 |  |  |  | 1 |  |  |  | 94 |  | 7 |  | 1 |  |  |  | 2 |  | 1 |  | 3 |  | 2 |  | 42 |  | 1 |  | 2 |  |  |  | 170 |  | 12 | 1 | 50 |  | 7 | 17 | 25 | 18 | 686 |  | 55 |  | 4 |
| TGG | 36 |  |  | 3 |  | 1 |  |  |  | 1 | 1 | 30 |  |  |  | 1 |  | 1 |  | 5 |  | 1 |  |  |  | 4 | 6 | 115 | 1 | 1 | 1 | 2 |  |  |  | 4 | 1 | 1 |  |  |  |  | 1 | 25 | 1 | 1 |  | 2 |  | 18 | 2 | 241 | 1 | 2 | 1 | 35 | 1 | 105 | 18 | 60 | 44 |  | 2 | 62 | 5 |
| TGT | 20 |  |  | 1 | 1 |  |  |  | 2 | 1 | 4 | 2 | 21 |  | 1 |  | 3 |  |  |  | 8 |  |  |  | 5 | 2 | 5 | 1 | 107 |  |  |  | 1 |  | 1 |  | 3 |  |  |  | 2 |  | 2 |  | 47 |  |  |  |  | 2 | 7 |  | 152 | 1 | 3 | 1 | 55 | 8 | 615 | 15 | 23 |  | 2 | 1 | 33 |
| TTA | 18 | 2 |  |  |  | 3 |  |  |  | 2 |  |  |  | 39 | 2 | 5 | 1 | 5 |  |  |  | 15 |  |  |  |  |  |  |  | 309 | 11 | 64 | 14 | 1 |  |  |  | 1 | 1 |  |  |  |  |  | 1 | 40 | 1 | 4 | 2 | 10 |  |  | 2 | 157 | 3 | 13 |  | 11 |  |  | 1 | 21 | 55 | 659 | 33 |
| TTC | 66 |  | 3 |  |  |  | 8 |  |  | 1 | 1 |  |  |  | 67 |  | 5 |  | 5 |  | 1 |  | 7 |  | 1 |  |  |  |  | 3 | 282 | 2 | 19 | 2 |  |  | 1 |  |  |  | 2 |  | 2 |  | 1 | 3 | 79 | 2 | 8 |  | 87 |  | 8 | 5 | 224 | 1 | 10 |  | 34 | 2 | 2 | 130 | 71 | 93 | 1,791 |
| TTG | 63 |  |  | 1 |  |  |  | 4 |  | 1 |  |  |  | 4 | 1 | 88 | 4 |  |  | 2 |  |  |  | 8 | 2 |  |  | 1 |  | 107 | 32 | 898 | 39 |  |  | 1 |  |  |  | 3 |  |  |  | 1 |  | 8 | 2 | 66 | 1 |  | 1 | 10 |  | 11 | 1 | 191 | 3 | 1 | 1 | 52 |  | 811 | 86 | 56 | 114 |
| TTT | 67 |  |  |  | 2 |  |  |  | 3 |  |  |  | 1 |  | 5 | 2 | 63 |  | 1 |  |  |  | 1 |  | 4 |  |  |  | 1 | 2 | 13 | 5 | 278 |  |  |  | 4 |  | 1 |  | 2 |  | 1 |  | 1 |  | 5 | 2 | 85 |  | 15 |  | 84 | 1 | 11 | 1 | 163 | 1 | 1 | 1 | 25 | 51 | 1,523 | 96 | 70 |


---


 **Amino acid changes**

How to read this table:   
- Rows are reference amino acids and columns are changed amino acids. E.g. Row 'A' column 'E' indicates how many 'A' amino acids have been replaced by 'E' amino acids.  
- Red background colors indicate that more changes happened (heat-map).  
- Diagonals are indicated using grey background color   
- WARNING: This table may include different translation codon tables (e.g. mamalian DNA and mitochondrial DNA).

|  | \* | - | ? | A | C | D | E | F | G | H | I | K | L | M | N | P | Q | R | S | T | V | W | Y |
| --- | --- | --- | --- | --- | --- | --- | --- | --- | --- | --- | --- | --- | --- | --- | --- | --- | --- | --- | --- | --- | --- | --- | --- |
| \* | 185 | 14 | 6 | 1 | 9 |  | 24 | 2 | 2 | 1 |  | 13 | 15 | 1 |  |  | 58 | 30 | 10 | 1 | 1 | 59 | 19 |
| - | 13 |  | 646 | 231 | 20 | 187 | 288 | 59 | 250 | 67 | 59 | 143 | 97 | 20 | 96 | 214 | 154 | 93 | 288 | 163 | 82 | 22 | 34 |
| ? |  |  |  |  |  |  |  |  |  |  |  |  |  |  |  |  |  |  |  |  |  |  |  |
| A | 1 | 410 | 13 | 8,674 | 12 | 251 | 264 | 10 | 372 | 3 | 60 | 22 | 34 | 15 | 21 | 302 | 6 | 15 | 700 | 2,200 | 1,863 | 2 | 4 |
| C | 27 | 46 | 5 | 7 | 1,349 | 7 |  | 94 | 92 | 11 | 5 | 1 | 3 |  | 4 | 6 |  | 219 | 162 | 2 | 2 | 33 | 341 |
| D | 1 | 333 | 10 | 191 | 3 | 4,778 | 1,063 | 4 | 1,012 | 212 | 5 | 38 | 3 | 2 | 1,509 | 3 | 16 | 13 | 49 | 21 | 120 | 2 | 226 |
| E | 107 | 522 | 11 | 210 |  | 1,072 | 3,651 | 3 | 741 | 9 | 13 | 1,382 | 7 | 7 | 40 | 7 | 445 | 51 | 26 | 25 | 122 | 1 | 6 |
| F | 1 | 126 | 7 | 5 | 62 | 5 | 2 | 3,455 | 5 | 7 | 140 |  | 974 | 2 | 5 | 13 |  | 2 | 418 | 11 | 184 | 3 | 194 |
| G | 38 | 498 | 16 | 397 | 136 | 949 | 655 |  | 8,555 | 15 | 12 | 31 | 4 | 5 | 54 | 8 | 17 | 682 | 962 | 32 | 248 | 41 | 11 |
| H | 3 | 75 | 4 | 5 | 13 | 190 | 11 | 5 | 7 | 1,846 | 2 | 5 | 110 | 1 | 222 | 108 | 407 | 428 | 14 | 9 | 3 | 1 | 669 |
| I |  | 135 | 11 | 56 | 5 | 10 | 10 | 132 | 15 | 3 | 5,239 | 46 | 487 | 604 | 127 | 7 | 5 | 51 | 132 | 925 | 1,785 |  | 5 |
| K | 33 | 332 | 15 | 18 |  | 41 | 1,056 |  | 33 | 15 | 50 | 3,250 | 10 | 107 | 678 | 9 | 371 | 866 | 35 | 238 | 8 | 1 | 7 |
| L | 35 | 327 | 20 | 23 | 6 | 5 | 11 | 1,014 | 8 | 104 | 534 | 6 | 12,959 | 257 | 8 | 744 | 110 | 206 | 432 | 24 | 486 | 58 | 13 |
| M | 1 | 69 | 6 | 19 |  | 1 | 7 | 1 | 2 | 1 | 783 | 84 | 249 | 83 | 8 | 3 | 8 | 64 | 13 | 292 | 314 | 1 | 1 |
| N | 1 | 159 | 6 | 17 | 5 | 1,078 | 34 | 3 | 44 | 192 | 119 | 596 | 6 | 1 | 3,279 | 4 | 15 | 26 | 884 | 240 | 13 |  | 97 |
| P | 2 | 537 | 19 | 287 | 8 | 7 | 3 | 13 | 6 | 180 | 13 | 6 | 1,063 | 2 | 9 | 6,431 | 171 | 183 | 1,246 | 326 | 11 | 2 | 5 |
| Q | 493 | 284 | 3 | 9 |  | 16 | 465 | 1 | 9 | 441 | 2 | 407 | 135 | 1 | 14 | 173 | 2,694 | 579 | 8 | 15 | 5 | 3 | 7 |
| R | 167 | 258 | 16 | 16 | 395 | 12 | 30 | 2 | 514 | 489 | 53 | 810 | 196 | 54 | 39 | 148 | 724 | 5,658 | 298 | 105 | 14 | 153 | 14 |
| S | 65 | 503 | 24 | 620 | 201 | 55 | 8 | 482 | 708 | 30 | 139 | 51 | 671 | 11 | 776 | 1,072 | 21 | 349 | 8,205 | 593 | 31 | 37 | 155 |
| T | 2 | 353 | 10 | 1,852 | 5 | 15 | 32 | 6 | 20 | 6 | 1,113 | 269 | 40 | 313 | 339 | 264 | 17 | 125 | 612 | 6,993 | 69 |  | 4 |
| V |  | 181 | 11 | 1,631 | 4 | 137 | 118 | 233 | 194 | 6 | 2,073 | 8 | 533 | 335 | 12 | 13 | 8 | 5 | 38 | 73 | 6,661 | 1 | 3 |
| W | 364 | 32 | 4 | 1 | 62 | 1 | 4 | 7 | 27 |  |  | 3 | 66 | 1 |  | 1 | 6 | 157 | 40 | 1 | 3 | 60 | 3 |
| Y | 78 | 79 | 2 | 2 | 322 | 159 | 6 | 184 | 5 | 495 | 5 | 1 | 15 |  | 75 | 3 | 11 | 11 | 98 | 2 | 2 | 7 | 2,482 |


---


 **Variants by chromosome**

```
		  

		FSP34_Chr01, Position,0,100000,200000,300000,400000,500000,600000,700000,800000,900000,1000000,1100000,1200000,1300000,1400000,1500000,1600000,1700000,1800000,1900000,2000000,2100000,2200000,2300000,2400000,2500000,2600000,2700000,2800000,2900000,3000000,3100000,3200000,3300000,3400000,3500000,3600000,3700000,3800000,3900000,4000000,4100000,4200000,4300000,4400000,4500000,4600000,4700000,4800000,4900000,5000000,5100000,5200000,5300000,5400000,5500000,5600000,5700000,5800000,5900000,6000000,6100000,6200000,6300000,6400000
FSP34_Chr01,Count,6009,1731,3631,3427,1711,485,413,295,391,212,276,143,91,246,113,109,480,108,192,111,214,154,135,105,196,75,266,127,201,85,157,166,93,101,175,158,156,148,170,172,132,411,170,174,129,128,370,126,309,149,271,218,328,269,185,393,1005,1005,925,1815,2091,3396,3221,1359,2

	
```

```
		  

		FSP34_Chr02, Position,0,100000,200000,300000,400000,500000,600000,700000,800000,900000,1000000,1100000,1200000,1300000,1400000,1500000,1600000,1700000,1800000,1900000,2000000,2100000,2200000,2300000,2400000,2500000,2600000,2700000,2800000,2900000,3000000,3100000,3200000,3300000,3400000,3500000,3600000,3700000,3800000,3900000,4000000,4100000,4200000,4300000,4400000,4500000,4600000,4700000,4800000,4900000,5000000
FSP34_Chr02,Count,158,3129,2149,1441,2075,1535,869,588,239,244,313,242,249,240,170,292,216,1029,161,159,133,149,1601,222,175,401,157,211,176,230,1014,873,229,300,267,388,989,181,364,322,363,857,1119,1400,2857,1847,1144,576,4177,1598,4611

	
```

```
		  

		FSP34_Chr03, Position,0,100000,200000,300000,400000,500000,600000,700000,800000,900000,1000000,1100000,1200000,1300000,1400000,1500000,1600000,1700000,1800000,1900000,2000000,2100000,2200000,2300000,2400000,2500000,2600000,2700000,2800000,2900000,3000000,3100000,3200000,3300000,3400000,3500000,3600000,3700000,3800000,3900000,4000000,4100000,4200000,4300000,4400000,4500000,4600000,4700000,4800000,4900000,5000000
FSP34_Chr03,Count,1431,1320,2232,1720,1899,1346,1029,458,469,1503,342,319,295,242,250,287,416,224,267,122,107,150,106,226,122,561,1055,602,214,162,433,289,193,149,96,154,440,112,136,226,232,452,471,297,665,647,1144,2635,1823,2564,3313

	
```

```
		  

		FSP34_Chr04, Position,0,100000,200000,300000,400000,500000,600000,700000,800000,900000,1000000,1100000,1200000,1300000,1400000,1500000,1600000,1700000,1800000,1900000,2000000,2100000,2200000,2300000,2400000,2500000,2600000,2700000,2800000,2900000,3000000,3100000,3200000,3300000,3400000,3500000,3600000,3700000,3800000,3900000,4000000,4100000,4200000,4300000
FSP34_Chr04,Count,4076,2423,2577,3143,1880,724,183,272,154,184,112,81,82,125,122,237,206,85,220,80,77,221,122,87,372,103,457,540,779,1716,499,1026,960,573,3985,419,1106,1072,1106,1609,4115,1956,2226,502

	
```

```
		  

		FSP34_Chr05, Position,0,100000,200000,300000,400000,500000,600000,700000,800000,900000,1000000,1100000,1200000,1300000,1400000,1500000,1600000,1700000,1800000,1900000,2000000,2100000,2200000,2300000,2400000,2500000,2600000,2700000,2800000,2900000,3000000,3100000,3200000,3300000,3400000,3500000,3600000,3700000,3800000,3900000,4000000,4100000,4200000,4300000,4400000
FSP34_Chr05,Count,3230,1801,2546,2161,926,821,469,1650,752,531,170,341,124,134,117,132,159,857,388,154,114,130,635,156,126,240,178,892,320,250,366,279,272,262,329,201,150,270,817,604,1442,914,1795,5253,2940

	
```

```
		  

		FSP34_Chr06, Position,0,100000,200000,300000,400000,500000,600000,700000,800000,900000,1000000,1100000,1200000,1300000,1400000,1500000,1600000,1700000,1800000,1900000,2000000,2100000,2200000,2300000,2400000,2500000,2600000,2700000,2800000,2900000,3000000,3100000,3200000,3300000,3400000,3500000,3600000,3700000,3800000,3900000,4000000,4100000,4200000,4300000
FSP34_Chr06,Count,4867,1237,3090,1382,1132,1541,428,769,256,376,357,328,541,369,262,275,291,199,250,279,246,246,366,318,191,208,203,195,330,244,215,247,629,987,1013,1499,3390,3704,2252,2429,1444,4089,1458,91

	
```

```
		  

		FSP34_Chr07, Position,0,100000,200000,300000,400000,500000,600000,700000,800000,900000,1000000,1100000,1200000,1300000,1400000,1500000,1600000,1700000,1800000,1900000,2000000,2100000,2200000,2300000,2400000,2500000,2600000,2700000,2800000,2900000,3000000,3100000,3200000,3300000,3400000,3500000
FSP34_Chr07,Count,2319,818,2006,2563,977,676,1016,1621,546,801,240,425,183,195,367,198,361,231,171,176,979,481,248,492,250,1000,1165,424,726,2127,1209,2736,1854,2332,1656,2689

	
```

```
		  

		FSP34_Chr08, Position,0,100000,200000,300000,400000,500000,600000,700000,800000,900000,1000000,1100000,1200000,1300000,1400000,1500000,1600000,1700000,1800000,1900000,2000000,2100000,2200000,2300000,2400000,2500000,2600000,2700000,2800000,2900000,3000000,3100000
FSP34_Chr08,Count,7737,2227,2279,4385,2580,1898,968,325,523,725,311,250,194,99,152,182,91,224,439,1403,1047,1644,398,1197,1362,1210,1823,2295,1413,663,989,2097

	
```

```
		  

		FSP34_Chr09, Position,0,10000,20000,30000,40000,50000,60000,70000,80000,90000,100000,110000,120000,130000,140000,150000,160000,170000,180000,190000,200000,210000,220000,230000,240000,250000,260000,270000,280000,290000,300000,310000,320000,330000,340000,350000,360000,370000,380000,390000,400000,410000,420000,430000,440000,450000,460000,470000,480000,490000,500000,510000,520000,530000,540000,550000,560000,570000,580000,590000,600000,610000,620000,630000,640000,650000,660000,670000,680000,690000,700000,710000,720000,730000,740000,750000,760000,770000,780000,790000,800000,810000,820000,830000,840000,850000,860000,870000,880000,890000,900000,910000,920000,930000,940000,950000,960000,970000,980000,990000,1000000,1010000,1020000,1030000,1040000,1050000,1060000,1070000,1080000,1090000,1100000,1110000,1120000,1130000,1140000,1150000,1160000,1170000,1180000,1190000,1200000,1210000,1220000,1230000,1240000,1250000,1260000,1270000,1280000,1290000,1300000,1310000,1320000,1330000,1340000,1350000,1360000,1370000,1380000,1390000,1400000,1410000,1420000,1430000,1440000,1450000,1460000,1470000,1480000,1490000,1500000,1510000,1520000,1530000,1540000,1550000,1560000,1570000,1580000,1590000,1600000,1610000,1620000,1630000,1640000,1650000,1660000,1670000,1680000,1690000,1700000,1710000,1720000,1730000,1740000,1750000,1760000,1770000,1780000,1790000,1800000,1810000,1820000,1830000,1840000,1850000,1860000,1870000,1880000,1890000,1900000,1910000,1920000,1930000,1940000,1950000,1960000,1970000,1980000,1990000,2000000,2010000,2020000,2030000,2040000,2050000,2060000,2070000,2080000,2090000,2100000,2110000,2120000,2130000,2140000,2150000,2160000,2170000,2180000,2190000,2200000,2210000,2220000,2230000,2240000,2250000,2260000,2270000,2280000,2290000,2300000,2310000,2320000,2330000,2340000,2350000,2360000,2370000,2380000,2390000,2400000,2410000,2420000,2430000,2440000,2450000,2460000,2470000,2480000,2490000,2500000,2510000,2520000,2530000,2540000,2550000,2560000,2570000,2580000,2590000,2600000,2610000,2620000,2630000,2640000,2650000,2660000,2670000,2680000,2690000,2700000,2710000,2720000,2730000,2740000,2750000,2760000,2770000,2780000,2790000,2800000,2810000,2820000,2830000,2840000,2850000,2860000,2870000,2880000,2890000,2900000,2910000,2920000,2930000,2940000,2950000,2960000,2970000,2980000
FSP34_Chr09,Count,311,1262,364,36,94,875,430,453,327,173,251,34,48,33,88,46,44,189,31,43,26,188,78,327,227,392,411,39,107,233,447,220,125,105,143,71,169,223,455,78,80,40,8,30,73,61,49,39,18,235,79,22,37,65,25,141,222,134,114,106,68,68,13,86,38,59,90,88,32,65,41,23,15,16,50,125,36,28,37,131,52,45,114,75,93,24,239,140,27,31,31,17,20,15,20,27,23,8,25,14,204,305,14,16,14,19,22,18,60,17,17,39,24,17,13,9,14,43,10,9,10,14,18,22,16,19,10,14,16,14,17,6,22,17,20,29,907,388,294,313,117,15,10,11,27,26,15,121,619,14,18,21,14,24,53,49,33,35,14,18,10,15,31,24,23,11,15,18,13,20,15,22,15,28,33,24,17,26,24,24,21,15,13,15,17,19,16,20,11,9,9,7,19,23,30,11,12,14,11,24,27,8,15,18,14,24,10,36,40,46,68,31,37,40,35,29,23,34,22,23,24,15,36,19,34,16,13,9,30,36,38,16,28,41,104,90,80,28,37,12,17,36,150,281,105,34,124,302,21,20,167,85,45,268,352,34,44,118,116,43,134,95,96,83,205,63,120,17,30,35,186,191,184,104,171,350,387,390,571,610,190,326,246,26,164,720,86,58,40,47,33,25,31,392,870,1048,1071,839,138

	
```

```
		  

		FSP34_Chr10, Position,0,10000,20000,30000,40000,50000,60000,70000,80000,90000,100000,110000,120000,130000,140000,150000,160000,170000,180000,190000,200000,210000,220000,230000,240000,250000,260000,270000,280000,290000,300000,310000,320000,330000,340000,350000,360000,370000,380000,390000,400000,410000,420000,430000,440000,450000,460000,470000,480000,490000,500000,510000,520000,530000,540000,550000,560000,570000,580000,590000,600000,610000,620000,630000,640000,650000,660000,670000,680000,690000,700000,710000,720000,730000,740000,750000,760000,770000,780000,790000,800000,810000,820000,830000,840000,850000,860000,870000,880000,890000,900000,910000,920000,930000,940000,950000,960000,970000,980000,990000,1000000,1010000,1020000,1030000,1040000,1050000,1060000,1070000,1080000,1090000,1100000,1110000,1120000,1130000,1140000,1150000,1160000,1170000,1180000,1190000,1200000,1210000,1220000,1230000,1240000,1250000,1260000,1270000,1280000,1290000,1300000,1310000,1320000,1330000,1340000,1350000,1360000,1370000,1380000,1390000,1400000,1410000,1420000,1430000,1440000,1450000,1460000,1470000,1480000,1490000,1500000,1510000,1520000,1530000,1540000,1550000,1560000,1570000,1580000,1590000,1600000,1610000,1620000,1630000,1640000,1650000,1660000,1670000,1680000,1690000,1700000,1710000,1720000,1730000,1740000,1750000,1760000,1770000,1780000,1790000,1800000,1810000,1820000,1830000,1840000,1850000,1860000,1870000,1880000,1890000,1900000,1910000,1920000,1930000,1940000,1950000,1960000,1970000,1980000,1990000,2000000,2010000,2020000,2030000,2040000,2050000,2060000,2070000,2080000,2090000,2100000,2110000,2120000,2130000,2140000,2150000,2160000,2170000,2180000,2190000,2200000,2210000,2220000,2230000,2240000,2250000,2260000,2270000,2280000,2290000,2300000,2310000,2320000,2330000,2340000,2350000,2360000,2370000,2380000,2390000,2400000,2410000,2420000,2430000,2440000,2450000,2460000,2470000,2480000,2490000,2500000,2510000,2520000,2530000,2540000,2550000,2560000,2570000,2580000,2590000,2600000,2610000,2620000,2630000,2640000,2650000,2660000,2670000,2680000,2690000
FSP34_Chr10,Count,292,845,1155,375,705,989,882,565,465,47,40,97,298,109,249,218,79,137,249,454,399,388,559,174,172,147,328,21,417,354,213,937,71,315,817,668,131,126,90,209,494,154,231,141,101,238,218,92,94,240,228,105,211,40,28,64,116,39,73,48,33,180,746,131,24,29,15,17,75,49,32,63,50,121,18,42,177,236,78,66,102,298,364,267,331,315,10,17,10,9,22,7,34,91,32,15,20,17,46,63,31,36,28,24,15,81,27,81,19,59,553,542,540,73,38,37,104,37,39,46,26,37,39,368,48,33,42,12,27,32,102,21,17,18,131,46,19,70,37,58,27,23,21,18,16,15,28,20,25,22,19,14,16,13,20,13,18,29,24,27,49,62,15,35,25,16,24,27,158,27,31,72,69,87,147,23,26,28,30,893,20,53,188,311,36,30,33,499,849,343,62,36,29,17,19,22,31,13,19,27,72,23,23,30,31,94,56,223,462,72,149,140,44,109,97,145,70,109,104,43,133,555,800,32,23,23,37,55,97,46,16,21,169,210,167,209,171,216,230,241,174,77,337,109,125,218,405,226,296,214,87,381,41,133,91,86,98,196,61,65,249,846,745,213,304,55,49,296,1197,474

	
```

```
		  

		FSP34_Chr11, Position,0,10000,20000,30000,40000,50000,60000,70000,80000,90000,100000,110000,120000,130000,140000,150000,160000,170000,180000,190000,200000,210000,220000,230000,240000,250000,260000,270000,280000,290000,300000,310000,320000,330000,340000,350000,360000,370000,380000,390000,400000,410000,420000,430000,440000,450000,460000,470000,480000,490000,500000,510000,520000,530000,540000,550000,560000,570000,580000,590000,600000,610000,620000,630000,640000,650000,660000,670000,680000,690000,700000,710000,720000,730000,740000,750000,760000,770000,780000,790000,800000,810000,820000,830000,840000,850000,860000,870000,880000,890000,900000,910000,920000,930000,940000,950000,960000,970000,980000,990000,1000000,1010000,1020000,1030000,1040000,1050000,1060000,1070000,1080000,1090000,1100000,1110000,1120000,1130000,1140000,1150000,1160000,1170000,1180000,1190000,1200000,1210000,1220000,1230000,1240000,1250000,1260000,1270000,1280000,1290000,1300000,1310000,1320000,1330000,1340000,1350000,1360000,1370000,1380000,1390000,1400000,1410000,1420000,1430000,1440000,1450000,1460000,1470000,1480000,1490000,1500000,1510000,1520000,1530000,1540000,1550000,1560000,1570000,1580000,1590000,1600000,1610000,1620000,1630000,1640000,1650000,1660000,1670000,1680000,1690000,1700000,1710000,1720000,1730000,1740000,1750000,1760000,1770000,1780000,1790000,1800000,1810000,1820000,1830000,1840000,1850000,1860000,1870000,1880000,1890000,1900000,1910000,1920000,1930000,1940000,1950000,1960000,1970000,1980000,1990000,2000000,2010000,2020000,2030000,2040000,2050000,2060000,2070000,2080000,2090000,2100000,2110000,2120000,2130000,2140000,2150000,2160000,2170000,2180000,2190000,2200000,2210000,2220000
FSP34_Chr11,Count,440,1005,950,853,101,615,65,36,32,39,39,59,374,166,163,200,378,196,34,253,140,140,118,320,157,36,21,444,169,300,345,477,407,178,108,152,237,166,239,231,131,343,273,465,426,175,122,197,143,34,408,52,142,48,155,24,111,143,69,193,235,80,198,80,86,100,80,42,208,108,35,34,33,47,207,419,543,434,369,382,670,204,108,23,32,25,26,23,37,60,49,137,439,487,213,480,241,457,53,0,378,32,107,82,58,77,62,43,60,42,60,202,447,125,54,81,97,117,80,76,24,46,26,27,17,21,23,23,31,24,23,16,75,114,82,34,25,20,121,98,422,530,191,50,39,36,20,59,52,168,65,136,108,103,63,79,37,61,196,55,48,48,56,99,235,280,91,142,56,90,233,247,245,166,128,283,51,52,175,173,154,85,33,56,65,116,57,114,336,214,169,348,97,205,46,103,215,51,55,374,136,73,129,53,105,31,79,140,234,354,66,88,125,254,276,75,45,22,32,427,350,729,0

	
```

```
		  

		FSP34_Chr12, Position,0,10000,20000,30000,40000,50000,60000,70000,80000,90000,100000,110000,120000,130000,140000,150000,160000,170000,180000,190000,200000,210000,220000,230000,240000,250000,260000,270000,280000,290000,300000,310000,320000,330000,340000,350000,360000,370000,380000,390000,400000,410000,420000,430000,440000,450000,460000,470000,480000,490000,500000,510000,520000
FSP34_Chr12,Count,697,172,86,253,248,225,205,84,47,56,59,34,27,77,69,176,249,681,212,570,448,551,660,554,188,221,809,418,742,683,478,408,608,772,553,250,369,216,48,225,407,73,133,374,400,81,127,457,143,436,657,340,258

	
```

```
		  

		FSP34_Contig01, Position,0,1000,2000,3000,4000,5000,6000,7000,8000,9000,10000,11000,12000,13000,14000,15000,16000,17000,18000,19000,20000,21000,22000,23000,24000,25000,26000,27000,28000,29000,30000,31000,32000,33000,34000,35000,36000,37000,38000,39000,40000,41000,42000,43000,44000,45000,46000,47000,48000,49000,50000,51000,52000,53000,54000,55000,56000,57000,58000,59000,60000,61000,62000,63000,64000,65000,66000,67000,68000,69000,70000,71000,72000,73000,74000,75000,76000,77000,78000,79000,80000,81000,82000,83000,84000,85000
FSP34_Contig01,Count,0,94,121,136,86,106,111,129,112,110,92,169,114,56,69,76,79,135,153,83,0,19,42,0,0,22,6,0,0,0,157,106,120,170,106,127,137,113,106,95,139,157,179,0,0,0,0,0,0,0,58,128,144,136,136,52,0,0,0,0,0,0,5,0,0,0,0,0,20,0,18,67,36,22,30,35,44,28,32,48,23,35,39,50,42,32

	
```

```
		  

		FSP34_Contig02, Position,0,100,200,300,400,500,600,700,800,900,1000,1100,1200,1300,1400,1500,1600,1700,1800,1900,2000,2100,2200,2300,2400,2500,2600,2700,2800,2900,3000,3100,3200,3300,3400,3500,3600,3700,3800,3900,4000,4100,4200,4300,4400,4500,4600,4700,4800,4900,5000,5100,5200,5300,5400,5500,5600,5700,5800,5900,6000,6100,6200,6300,6400,6500,6600,6700,6800,6900,7000,7100,7200,7300,7400,7500,7600,7700,7800,7900,8000,8100,8200,8300,8400,8500,8600,8700,8800,8900,9000,9100,9200,9300,9400,9500,9600,9700,9800,9900,10000,10100,10200,10300,10400,10500,10600,10700,10800,10900,11000,11100,11200,11300,11400,11500,11600,11700,11800,11900,12000,12100,12200,12300,12400,12500,12600,12700,12800,12900,13000,13100,13200,13300,13400,13500,13600,13700,13800,13900,14000,14100,14200,14300,14400,14500,14600,14700,14800,14900,15000,15100,15200,15300,15400,15500,15600,15700,15800,15900,16000,16100,16200,16300,16400,16500,16600,16700,16800,16900,17000,17100,17200,17300,17400,17500,17600,17700,17800,17900,18000,18100,18200,18300,18400,18500,18600,18700,18800,18900,19000,19100,19200,19300,19400,19500,19600,19700,19800,19900,20000,20100,20200,20300,20400,20500,20600,20700,20800,20900,21000,21100,21200,21300,21400,21500,21600,21700,21800,21900,22000,22100,22200,22300,22400,22500,22600,22700,22800,22900,23000,23100,23200,23300,23400,23500,23600,23700,23800,23900,24000,24100,24200,24300,24400,24500,24600,24700,24800,24900,25000,25100,25200,25300,25400,25500,25600,25700,25800,25900,26000,26100,26200,26300,26400,26500,26600,26700,26800,26900,27000,27100,27200,27300,27400,27500,27600,27700
FSP34_Contig02,Count,0,0,0,0,0,0,0,0,0,0,0,0,0,0,0,0,0,0,0,0,0,0,0,0,0,0,0,0,0,0,0,0,0,0,0,10,10,6,13,15,5,11,15,22,20,6,0,12,16,12,15,14,8,10,7,12,10,8,12,14,9,15,20,12,13,14,12,14,11,12,10,11,5,3,6,16,11,13,17,16,16,13,12,14,16,8,10,10,4,7,10,12,9,9,17,15,15,14,19,15,12,16,21,15,12,15,16,18,18,12,9,17,1,0,0,9,15,12,14,10,14,21,15,19,15,13,9,13,10,8,18,12,12,13,13,13,9,10,12,11,17,11,10,11,17,19,10,14,15,15,11,15,11,9,14,15,16,16,12,2,8,16,15,8,13,15,8,11,8,12,7,9,6,13,16,15,20,10,10,7,8,9,4,3,0,0,0,0,0,0,0,0,0,0,0,0,0,0,0,0,0,0,0,0,0,0,0,0,0,0,0,0,0,0,0,0,0,0,0,0,9,5,8,4,6,0,0,0,0,0,0,0,0,0,0,0,0,0,0,0,0,0,0,0,0,0,0,12,5,8,3,0,0,0,2,5,0,0,0,0,0,0,0,0,0,0,0,0,0,0,0,0,0,0,0,0,0,0

	
```

```
		  

		FSP34_Mitochondrion, Position,0,1000,2000,3000,4000,5000,6000,7000,8000,9000,10000,11000,12000,13000,14000,15000,16000,17000,18000,19000,20000,21000,22000,23000,24000,25000,26000,27000,28000,29000,30000,31000,32000,33000,34000,35000,36000,37000,38000,39000,40000,41000,42000,43000,44000,45000,46000,47000,48000,49000,50000,51000,52000,53000,54000,55000,56000,57000,58000,59000,60000,61000,62000,63000,64000,65000,66000,67000,68000,69000,70000,71000,72000,73000,74000,75000,76000,77000,78000,79000,80000,81000
FSP34_Mitochondrion,Count,0,0,0,0,0,0,0,0,0,0,0,1,2,2,1,3,0,0,0,0,0,0,0,1,0,0,2,0,0,1,1,0,0,0,0,0,1,0,1,0,1,0,0,1,1,0,0,0,0,0,8,0,0,0,0,3,0,0,3,2,0,1,0,0,0,0,0,0,0,0,0,2,0,2,0,1,0,1,1,0,2,0

	
```


---

 **Details by gene** 

**Here** you can find a tab-separated table.
